# Ruthenium‐Catalyzed C—H Alkenylation of Trypanocidal Naphthoquinones: A Mechanistic Benchmarking Study

**DOI:** 10.1002/open.202500465

**Published:** 2025-11-05

**Authors:** Esther R. S. Paz, Cauê P. Souza, Joyce C. De Oliveira, Renata G. Almeida, Chonny Herrera‐Acevedo, Sulaiman Lakoh, Guilherme A. M. Jardim, Eufrânio N. da Silva Júnior, Felipe Fantuzzi

**Affiliations:** ^1^ Instituto de Ciências Exatas Departamento de Química Universidade Federal de Minas Gerais Belo Horizonte MG 31270–901 Brazil; ^2^ Chemistry and Forensic Science School of Natural Sciences University of Kent Park Wood Road Canterbury CT2 7NH UK; ^3^ Department of Chemical Engineering Universidad ECCI Carrera 19 # 49–20 111311 Bogotá D.C. Colombia; ^4^ Department of Internal Medicine College of Medicine and Allied Health Sciences University of Sierra Leone Freetown Sierra Leone

**Keywords:** C—H activation, density functional calculations, drug discovery, quinones, ruthenium

## Abstract

Quinones are privileged scaffolds in biological redox chemistry and drug discovery, but methods to install versatile click handles onto their cores remain scarce. This work presents a comprehensive computational study of the Ru(II)‐catalyzed C—H alkenylation of menadione with ethenesulfonyl fluoride, a transformation that introduces sulfonyl‐fluoride groups for subsequent SuFEx chemistry. Nine density functionals—from GGAs to double hybrids—are first benchmarked against DLPNO‐CCSD(T) reference energies for all key on‐cycle intermediates and transition states along the cationic [Ru(OAc)(*p*‐cymene)]^+^ pathway. Among them, *ω*B2PLYP best matches the coupled‐cluster reference and is the only method to achieve root‐mean‐square deviations of ≈1 kcal mol^−1^. Given that the computed on‐cycle barriers are modest, the results indirectly support that the overall rate is dictated by off‐cycle formation of the active cationic species via ligand exchange/speciation. Within the catalytic cycle, C—H activation presents the highest global barrier, although migratory insertion can display a higher local barrier (relative to its immediate precursor) for specific ring substitutions. Finally, it is shown that the r^2^SCAN‐3c composite method offers a computationally efficient route for probing analogous catalytic cycles. These results deliver a robust protocol for designing naphthoquinone derivatives as next‐generation therapeutic agents against *Trypanosoma cruzi* and related parasites.

## Graphical abstract

Computational roadmap to click‐ready quinones. Quantum chemical analysis reveals how substituents modulate the reactivity of trypanocidal naphthoquinones in Ru‐catalyzed C—H alkenylation, guiding the design of sulfonyl‐fluoride scaffolds for next‐generation antiparasitic agents.

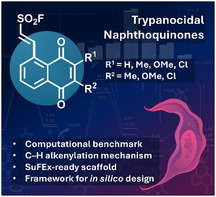

## 1 Introduction

Quinones are privileged scaffolds that play key roles in medicine and biology due to their redox behavior, structural variety, and clinical relevance.^[^ ^1^, ^2^, ^3^, ^4^ ^–^ ^5^ ^]^ They mediate essential bioenergetic processes, most notably electron transport and cellular respiration.^[^ ^6^, ^7^ ^–^ ^8^ ^]^ For instance, ubiquinone (coenzyme Q), plastoquinone, and menaquinone are renowned examples of electron transporters in mitochondria and chloroplasts, contributing to energy production and metabolic balance.^[^ ^9^, ^10^ ^–^ ^11^ ^]^ Their reversible redox cycling and capacity for covalent adduct formation underpin a broad spectrum in pharmacology, including anticancer, antimicrobial, antiparasitic, and anti‐inflammatory activities.^[^ ^12^, ^13^, ^14^, ^15^ ^–^ ^16^ ^]^

Therapeutically, the quinoidal core is especially promising against neglected tropical diseases (NTDs).^[^ ^17^ ^]^ In Brazil, Chagas disease, caused by *Trypanosoma cruzi*, remains a priority given the limitations and side effects of current drugs.^[^ ^18^, ^19^ ^–^ ^20^ ^]^ In Africa, Human African Trypanosomiasis (HAT), or sleeping sickness, persists in several regions despite substantial progress toward elimination. Gambiense HAT, the chronic form caused by *Trypanosoma brucei gambiense*, remains endemic in 24 countries across West and Central Africa, including Angola, the Central African Republic, Chad, Guinea, South Sudan, Cameroon, and the Democratic Republic of the Congo, with the latter alone accounting for over 60% of the total cases in the past 5 years.^[^ ^21^ ^,^ ^22^ ^]^ Although some countries, such as Sierra Leone, no longer report routine cases, continued surveillance is critical due to the focal and re‐emerging nature of the disease.^[^ ^23^ ^]^ Following the successful elimination of HAT as a public health threat, the current objective is to achieve zero transmission of gambiense HAT by 2030.^[^ ^22^ ^]^ The rhodesiense form, sustained by zoonotic transmission, affects East and Southern Africa.^[^ ^23^ ^]^ Both suffer from limited, often toxic therapies with challenging logistics.^[^ ^24^, ^25^ ^–^ ^26^ ^]^ Advances in the selective functionalization of quinoidal scaffolds now enable tuning of electronic and physicochemical properties, facilitating structure–activity relationship (SAR)‐driven discovery of safer, more effective NTD therapeutic candidates.^[^ ^27^, ^28^, ^29^ ^–^ ^30^ ^]^

**Scheme 1:**
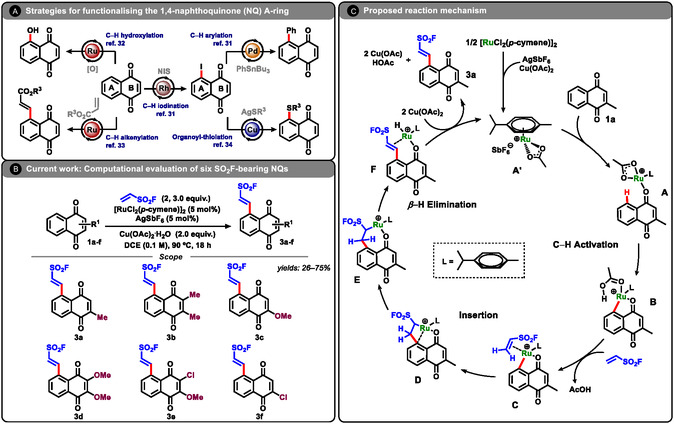
A) Representative synthetic strategies for functionalizing the A‐ring of 1,4‐naphthoquinones. B) Reaction conditions for the Ru(II)‐catalyzed C—H alkenylation with ethenesulfonyl fluoride, yielding SO_2_F‐functionalized naphthoquinones; structures computationally investigated in this study (**3a**–**3f**) are also shown. Isolated yields across **3a**–**3f**: 26–75%. Full experimental procedures and individual yields are reported in Ref. [47]. C) Proposed catalytic cycle for the formation of SO_2_F‐bearing naphthoquinones via C—H functionalization.

Building on this context, the da Silva Júnior group has developed methodologies to functionalize both the redox center (B‐ring) and the benzenoid ring (A‐ring) of naphthoquinones, identifying promising anti‐*T. cruzi* candidates (**Scheme** **1**A).^[^ ^31^ ^–^ ^34^ ^]^ Notably, A‐ring modifications enhance activity against trypomastigotes.^[^ ^32^, ^33^ ^–^ ^35^ ^]^ In parallel, sulfur(VI) fluoride exchange (SuFEx) has emerged as a reliable, chemoselective click platform for installing metabolically robust sulfur(VI) motifs with favorable pharmacokinetic profiles,^[^ ^36^, ^37^, ^38^, ^39^ ^–^ ^40^, ^41^, ^42^, ^43^, ^44^, ^45^ ^]^ while its mild, modular conditions support late‐stage diversification for rapid lead optimization.^[^ ^36^ ^–^ ^46^ ^]^

Recently, we reported a Ru‐catalyzed C—H alkenylation that installs an ethenesulfonyl fluoride at C‐5 of the naphthoquinone A‐ring, furnishing SuFEx‐ready products (Scheme 1B).^[^ ^47^ ^]^ Scheme 1C summarizes the proposed catalytic working cycle. i) AgSbF_6_ abstracts chloride from [RuCl_2_(*p*‐cymene)]_2_ (AgCl formation; *p*‐cymene = *p*‐isopropyltoluene), and acetate supplied by Cu(OAc)_2_ binds at ruthenium to generate the cationic Ru–acetate species **A′** = [Ru(OAc)(*p*‐cymene)]^+^[SbF_6_]^−^. ii) *κ*‐O coordination of menadione (**1a**) to **A′** furnishes the on‐cycle adduct **A**. iii) Acetate‐assisted C—H activation (concerted metalation–deprotonation, CMD) forms the Ru–aryl intermediate **B**. iv) Coordination of ethenesulfonyl fluoride leads to **C**, which is followed by migratory insertion to forge the new C—C bond in **D** and the ruthenacycle **E**. v) *β*‐Hydride elimination from **E** furnishes **F**, a Ru(II)–H species in which the alkenylated quinone remains bound via *κ*‐O coordination to the carbonyl and weak *η* ^2^‐alkene coordination to the newly formed double bond. vi) From **F**, two fast, interchangeable events complete turnover: a) acetate‐assisted ligand exchange weakens displacement of the product from Ru; b) oxidation of the Ru–H by Cu(OAc)_2_ via an acetate bridge converts Ru–H to Ru–OAc, generating Cu^I^OAc and HOAc.

Whether hydride oxidation precedes or follows product dissociation, the outcome is the same: the alkenylated quinone is released and the cationic Ru–acetate **A′** is regenerated for the next cycle.

Density functional theory (DFT) has been widely used to elucidate Ru(II)‐catalyzed C—H activation, especially carboxylate‐assisted pathways.^[^ ^48^ ^–^ ^53^ ^]^ Yet, comparatively few studies systematically benchmark functionals against high‐level references for these transformations. Here, using the C—H alkenylation of **1a** with ethenesulfonyl fluoride along the cationic [Ru(OAc)(*p*‐cymene)]^+^[SbF_6_]^−^ pathway as a case study, we benchmark a broad set of DFT methods (GGAs, hybrids, double hybrids, plus r^2^SCAN‐3c) against DLPNO‐CCSD(T) reference energies for all key on‐cycle intermediates and transition states. Our goals are twofold: i) to identify the most appropriate methodology for accurately describing the central transition states—**TS1** (C—H activation), **TS2** (alkene insertion), and **TS3** (*β*‐hydride elimination), and ii) to probe how substituents on the menadione ring modulate the barriers of these steps. By systematically varying substituent identity and position, we aim to establish structure–reactivity relationships that can guide the design of new bioactive quinone derivatives for SuFEx click chemistry.

## 2 Computational Methods

We analyzed the mechanism for **1a** using nine density functionals benchmarked against DLPNO‐CCSD(T)^[^ ^54^, ^55^, ^56^ ^–^ ^57^ ^]^ reference energies. Following Jacob's ladder,^[^ ^58^ ^]^ we selected functionals from different rungs: the pure GGAs PBE^[^ ^59^ ^]^ and BP86,^[^ ^60^ ^,^ ^61^ ^]^ the hybrids B3LYP^[^ ^61^ ^,^ ^62^ ^]^ and PBE0,^[^ ^63^ ^]^ the meta‐GGA pure functional M06‐L,^[^ ^64^ ^]^ the hybrid meta‐GGA M06,^[^ ^65^ ^]^ the range‐separated hybrid *ω*B97X‐D3,^[^ ^66^ ^,^ ^67^ ^]^ and the double‐hybrids B2PLYP^[^ ^68^ ^]^ and *ω*B2PLYP.^[^ ^69^ ^]^ This set spans diverse approximations to assess performance for the C—H alkenylation mechanism.

Accuracy was assessed by comparing the free energies of key intermediates and transition states with DLPNO‐CCSD(T) references. The root‐mean‐square deviation (RMSD), mean absolute deviation (MAD), and extremal absolute errors (maximum and minimum) relative to the coupled‐cluster results were reported to identify the most reliable DFT options for reaction energetics. All calculations incorporated dispersion corrections using Grimme's D3 method.^[^ ^70^ ^]^ In particular, the Minnesota functionals M06 and M06‐L, *ω*B97X‐D3, and *ω*B2PLYP employed the pure D3 correction, whereas the remaining functionals were used in conjunction with Grimme's D3 correction featuring Becke–Johnson damping, D3(BJ).^[^ ^71^ ^]^

Geometry optimizations were carried out using the PBE0‐D3(BJ) functional with the def2‐SVP basis set for all atoms except for Ru, for which the def2‐TZVP basis set was employed.^[^ ^72^ ^]^ Vibrational frequency analyses were subsequently performed to confirm that each optimized structure corresponds to a true minimum (i.e., no imaginary frequencies) or to a transition state (characterized by a single imaginary frequency). Intrinsic reaction coordinate (IRC)^[^ ^73^ ^]^ calculations were then executed from each transition state to verify that the computed pathways correctly connect the relevant reactants and products.

Single‐point energy calculations were subsequently performed using all nine selected functionals—alongside high‐level DLPNO‐CCSD(T) computations—to generate a detailed energy profile. For these single‐point calculations, the def2‐TZVPP basis set was applied for all atoms to achieve enhanced accuracy.^[^ ^72^ ^]^ The double‐hybrid functionals needed to use the def2‐TZVPP/c auxiliary basis set for the RI‐MP2 calculations.^[^ ^74^ ^]^ Solvent effects were incorporated into both the optimization and single‐point calculations via the conductor‐like polarizable continuum model (CPCM),^[^ ^75^ ^]^ using parameters for 1,2‐dichloroethane (DCE; dielectric constant *ε* = 10.36 and refractive index *η* = 1.44), which reflects the solvent conditions employed in the experimental studies. Finally, a concentration correction of 1.89 kcal mol^−1^ was applied to adjust the free energies from a gas‐phase standard state (1.00 atm) to a solution‐phase standard state (1.00 mol L^−^^1^), ensuring that the computed thermodynamic parameters accurately reflect the experimental environment.^[^ ^76^, ^77^ ^–^ ^78^ ^]^

Using the optimal DFT functional from the benchmark, substituent effects on the B‐ring of **1a** (selected for its synthetic flexibility under the C—H alkenylation conditions) were examined. As shown in Scheme 1B, representative electron‐donating groups were introduced on the B‐ring and their influence on the key activation barriers and intermediate stabilities was quantified, enabling the derivation of structure–reactivity trends pertinent to SuFEx‐enabled derivatization.

Finally, the recently proposed r^2^SCAN‐3c composite method,^[^ ^79^ ^]^ which combines the r^2^SCAN meta‐GGA functional with D4 dispersion^[^ ^80^ ^]^ and counterpoise corrections, was evaluated as a cost‐effective option for geometry optimization of the full on‐cycle mechanism prior to DLPNO‐CCSD(T) single‐point energies. All calculations were performed using ORCA version 5.0.3.^[^ ^81^ ^]^ 3D representations of the optimized structures were generated using CYLview.^[^ ^82^ ^]^

## 3 Results and Discussion

### 3.1 Overview of the Reaction Mechanism

**Figure 1:**
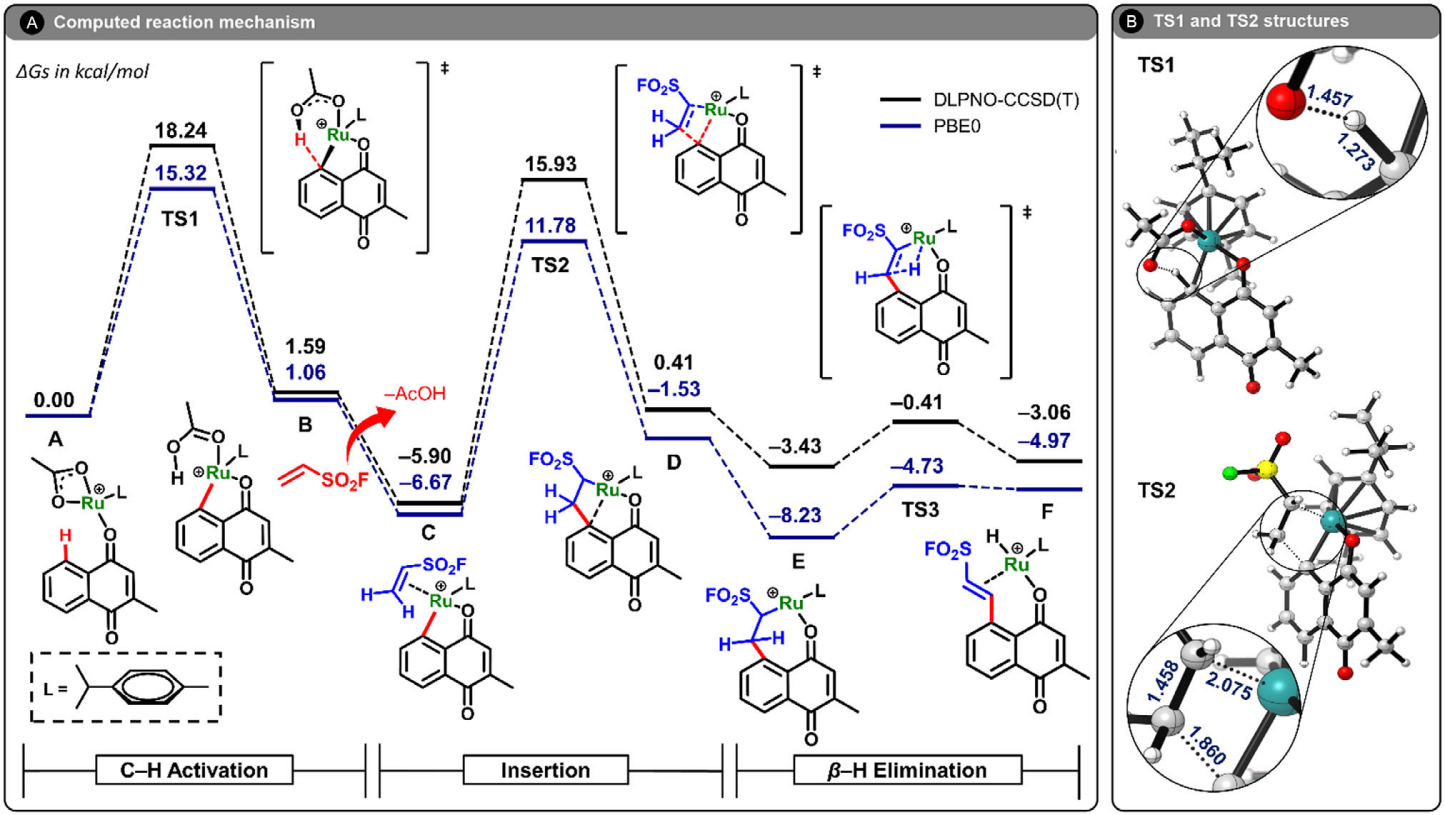
A) Computed Gibbs free energies (in kcal mol^−1^) for the C—H alkenylation of the mono‐methylated 1,4‐naphthoquinone **1a**. Energies are at the PBE0‐D3(BJ)/def2‐TZVPP+CPCM(DCE) and DLPNO‐CCSD(T)/def2‐TZVPP+CPCM(DCE) level of theory, from optimized structures at PBE0‐D3(BJ)/bs1+CPCM(DCE). B) Transition‐state structures associated with the C—H activation (**TS1**) and olefin insertion (**TS2**) steps, with relevant bond lengths given in Å. See text for more details.

In our previous study,^[^ ^47^ ^]^ the Ru(II)‐catalyzed C—H alkenylation of **1a** with ethenesulfonyl fluoride **2** (which installs an SO_2_F handle for SuFEx) was examined using PBE0‐D3(BJ)/bs1+CPCM(DCE) (bs1 = def2‐TZVP for Ru and def2‐SVP for all other atoms) geometries and frequencies, with refined single‐point energies at the PBE0‐D3(BJ)/def2‐TZVPP + CPCM(DCE) level. Here, the cycle is revisited with DLPNO‐CCSD(T) single points as a high‐level reference and directly compared with updated PBE0‐D3(BJ) results. **Figure** **1**A shows the computed energy profiles using both methods, and Figure 1B depicts the on‐cycle transition state structures **TS1** (C—H activation) and **TS2** (migratory insertion). Global free energies (ΔG) are referenced to **A** (set to 0.00 kcal mol^−1^); local changes ($\text{Δ}G_{Y-X}$) denote the free‐energy difference between species ***Y*** and its immediate precursor ***X***, distinguishing global from local barriers.^[^ ^83^ ^]^

The mechanism begins with the coordination of the substrate to [Ru(OAc)(*p*‐cymene)]^+^, forming intermediate **A**. This is followed by C—H activation via a six‐membered transition state (**TS1**), leading to intermediate **B**. The energy barrier for **TS1** is calculated as ΔG = +18.24 kcal mol^−1^ at DLPNO‐CCSD(T), while PBE0 estimates it at ΔG = +15.32 kcal mol^−1^, consistent with the common trend of underestimating barriers at the DFT level. Intermediate **B** is modestly endergonic, with ΔG values of +1.59 kcal mol^−1^ (DLPNO‐CCSD(T)) and +1.06 kcal mol^−1^ (PBE0).

Upon reinvestigating the subsequent steps of the mechanism, we identified a more stable conformer of intermediate **C** than previously reported. IRC calculations from **TS2** (see Figure S2, Supporting Information) confirm that this newly located conformer is indeed the structure directly connected to **TS2**, and not the less stable geometry used in our earlier work.^[^ ^47^ ^]^ This finding alters the thermodynamic profile of this reaction step: while our previous study indicated that the transformation from **B** to **C** was slightly endergonic, the revised mechanism shows that this step is in fact exergonic, with $\text{Δ}G_{C-B}$ = −7.49 kcal mol^−1^ (DLPNO‐CCSD(T)) and −7.73 kcal mol^−1^ (PBE0), while ΔG = −5.90 kcal mol^−1^ (DLPNO‐CCSD(T)) and −6.67 kcal mol^−1^ (PBE0).

The migratory insertion step proceeds via **TS2**, with a calculated local barrier of $\text{Δ}G_{\mathrm{TS}2-C}^{‡}$ = +21.83 kcal mol^−1^ (DLPNO‐CCSD(T)) and +18.45 kcal mol^−1^ (PBE0). This again reveals the underestimation typical of hybrid DFT functionals, yet the qualitative features of the energy surface remain accurate. The resulting intermediate **D** lies at +0.41 kcal mol^−1^ and −1.53 kcal mol^−1^ relative to **A** at the DLPNO‐CCSD(T) and PBE0 levels, respectively.

Formation of the seven‐membered ruthenacycle **E** occurs via conformational reorganization and ligand recoordination. This step is exergonic, yielding **E** at ΔG = −3.43 kcal mol^−1^ (DLPNO‐CCSD(T)) and −8.23 kcal mol^−1^ (PBE0), with $\text{Δ}G_{E-D}$ = −3.84 kcal mol^−1^ (DLPNO‐CCSD(T)) and −6.70 kcal mol^−1^ (PBE0). Finally, *β*‐hydride elimination takes place through **TS3**, with a local energy barrier of $\text{Δ}G_{\mathrm{TS}3-E}^{‡}$ = +3.02 kcal mol^−1^ (DLPNO‐CCSD(T)) and +3.50 kcal mol^−1^ (PBE0), leading to the hydride species **F** at ΔG = −3.06 kcal mol^−1^ and −4.97 kcal mol^−1^, respectively. **F** is a Ru(II)–H complex in which the alkenylated quinone remains bound via *κ*‐O (carbonyl) and weak *η* ^2^‐alkene coordination. Product release (**3a**) in concert with acetate recoordination regenerates the active cationic Ru(II)–acetate species and completes the on‐cycle sequence.

To further analyze the kinetic features of the catalytic cycle, we compare global and local barriers from each method. The C—H activation barrier $\text{Δ}G_{\mathrm{TS}1-A}^{‡}$ = +18.24 kcal mol^−1^ at DLPNO‐CCSD(T) and +15.32 kcal mol^−1^ at PBE0. Migratory insertion is locally higher: $\text{Δ}G_{\mathrm{TS}2-C}^{‡}$ = +21.83 and +18.45 kcal mol^−1^, respectively. *β*‐Hydride elimination remains much lower in both cases ($\text{Δ}G_{\mathrm{TS}3-E}^{‡}=$ +3.02 and + 3.50 kcal mol^−1^).

Across both profiles, **TS1** is the highest global on‐cycle barrier, so C—H activation is the on‐cycle rate‐determining step (RDS); in energetic‐span terms, **TS1** corresponds to the turnover frequency (TOF)‐determining transition state.^[^ ^84^ ^,^ ^85^ ^]^ Notably, for **1a** the local barrier for migratory insertion exceeds that for C—H activation, consistent with **C** being the most stable intermediate within the **A**–**F** manifold at the DLPNO‐CCSD(T) level. This identifies migratory insertion as a sensitive control point: modest changes in substituents or conditions could raise this stepwise barrier enough for migratory insertion to become the highest global on‐cycle barrier. We return to this point in the substituent‐effects section below.

The most stable resting intermediate differs by method: **C** at DLPNO‐CCSD(T) (ΔG = −5.90 kcal mol^−1^) versus **E** at PBE0 (ΔG = −8.23 kcal mol^−1^). Even so, both approaches yield qualitatively consistent mechanisms, with modest on‐cycle barriers consistent with efficient turnover once the active cationic species is formed. However, PBE0 systematically underestimates the activation barriers relative to DLPNO‐CCSD(T), most notably for **TS1** and **TS2**. This compression of the energy landscape may lead to underestimation of kinetic barriers and highlights the need for high‐level validation when using DFT methods in catalytic studies. Nevertheless, the ability of PBE0 to recover all stationary points and reproduce the correct energetic ordering confirms its utility for rapid screening, especially when calibrated against more accurate references.

Beyond the on‐cycle landscape, the modest computed barriers point to a scenario in which the overall rate is dictated by off‐cycle preactivation—namely, halide abstraction and acetate delivery that generate the cationic Ru(II)–acetate manifold, followed by substrate binding and speciation (including possible contact‐ion pairing). In this view, formation of the active cationic species precedes and limits turnover, whereas within the cycle C—H activation remains the highest global barrier. This interpretation is consistent with prior analyses that emphasized the kinetic relevance of Ru pre‐equilibria and ligand exchange,^[^ ^53^ ^]^ and it also aligns with the need for elevated reaction temperature (≈90 °C). Such conditions are more readily rationalized by endergonic ligand‐exchange/ionization steps than by the relatively modest on‐cycle barriers alone, further supporting an off‐cycle bottleneck.

### 3.2 Benchmark of DFT Functionals

**Figure 2:**
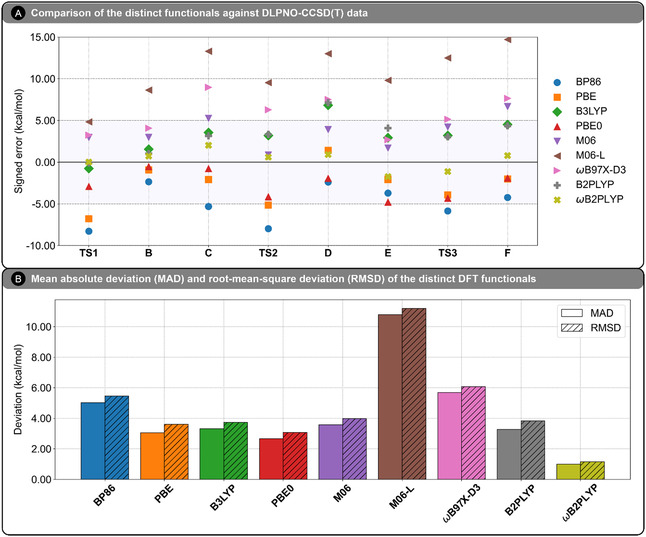
A) Signed errors (in kcal mol^−1^) of the nine density functional methods relative to DLPNO‐CCSD(T) reference energies. B) Mean absolute deviation (MAD) and root‐mean‐square deviation (RMSD) values (in kcal mol^−1^) for the same functionals, computed against DLPNO‐CCSD(T) reference data.

In this section, we evaluate the performance of a broad selection of density functionals in modeling the mechanism of the Ru(II)‐catalyzed C—H alkenylation of naphthoquinones with ethenesulfonyl fluoride. Once more, **1a** was chosen as the representative substrate for this benchmark study. Consistent with the format of Figure 1A, the ΔGs of all species along the catalytic cycle were compared against DLPNO‐CCSD(T) values, with intermediate **A** set to 0.00 kcal mol^−1^. Nine DFT functionals spanning different rungs of Jacob's ladder were then selected for comparison (see Computational Methods for details). Complete reaction energy profiles and minimum energy pathways (MEPs) for each functional are provided in the Supporting Information (Figure S4–S7 and Table S1–S13). **Figure** **2** summarizes the benchmarking results by depicting the signed errors relative to the DLPNO‐CCSD(T) reference (Figure 2A) and the corresponding MAD and RMSD values for each functional (Figure 2B).

To contextualize these benchmarking results, we organize the discussion by functional class. For each group of methods, we assess their ability to reproduce the DLPNO‐CCSD(T) reference energetics—focusing on the stability of key intermediates and the relative heights of transition‐state barriers—while drawing on the visual and quantitative data presented in Figure 2.

We begin with the GGA functionals, BP86 and PBE. As illustrated in Figure 2A, both GGA functionals systematically underestimate the Gibbs free energies of intermediates and transition states relative to the DLPNO‐CCSD(T) reference. The largest discrepancies are observed for the kinetically relevant transition states **TS1** and **TS2**. For BP86, the deviations are −8.28 kcal mol^−1^ (**TS1**) and −7.98 kcal mol^−1^ (**TS2**), while for PBE, the corresponding errors are −6.79 and −5.16 kcal mol^−^^1^. This translates into predicted ΔG values of +9.96 and +11.45 kcal mol^−^^1^ for **TS1** using BP86 and PBE, respectively, and +7.95 and +10.77 kcal mol^−^^1^ for **TS2**—substantially lower than the DLPNO‐CCSD(T) values of +18.24 kcal mol^−^^1^ for **TS1** and +15.93 kcal mol^−^^1^ for **TS2**. Among the intermediates, structure **C** is the most overstabilized in BP86 (signed error: −5.32 kcal mol^−^^1^), while **E** shows the largest stabilization in PBE (signed error: −2.10 kcal mol^−^^1^). All species are systematically more stable than their DLPNO‐CCSD(T) counterparts, with the sole exception of intermediate **D** in PBE, which exhibits a small positive signed error of +1.41 kcal mol^−1^. These tendencies are reflected in the overall performance metrics: PBE shows lower MAD and RMSD values (+3.06 kcal mol^−1^ and +3.61 kcal mol^−1^, respectively) than BP86 (+5.02 and +5.45 kcal mol^−1^). For BP86, the largest deviation (LD) occurs at **TS1** (−8.28 kcal mol^−1^), while the smallest deviation (SD) appears at intermediate **B** (−2.35 kcal mol^−1^); PBE displays the same trend with an LD of −6.79 kcal mol^−1^ and an SD of −0.93 kcal mol^−1^.

Despite quantitative discrepancies, both GGAs preserve the qualitative features of the profile. The resting state is correctly assigned as **C** (ΔG = −11.22 kcal mol^−1^ for BP86 and −7.99 kcal mol^−1^ for PBE versus −5.90 kcal mol^−1^ at DLPNO‐CCSD(T)). The rate‐limiting on‐cycle step is likewise attributed to C—H activation, with the **TS1**–**TS2** gap of +2.01 (BP86) and +0.68 kcal mol^−1^ (PBE) close to the DLPNO‐CCSD(T) value (+2.31 kcal mol^−1^). Both methods also identify migratory insertion as the local maximum: $\text{Δ}G_{\mathrm{TS}2-C}^{‡}$ = +19.17 (BP86) and +18.76 kcal mol^−1^ (PBE), versus +21.83 kcal mol^−1^ at the coupled‐cluster level. Thus, while GGA functionals compress barriers, they still capture the essential mechanistic ordering and key stationary points.

It is important to note that benchmarking studies of this type inevitably involve small systematic errors associated with the reference method (DLPNO‐CCSD(T)) and the free‐energy evaluation protocol. These include basis set incompleteness, local correlation approximations, and harmonic oscillator treatments for entropic contributions. However, these effects are expected to remain within chemical accuracy (<1.00 kcal mol^–1^) and to affect all stationary points similarly, so that relative deviations between DFT and DLPNO‐CCSD(T) provide a meaningful measure of functional performance.

Furthermore, it is unsurprising that DFT errors are generally larger for transition states than for intermediates. Transition structures feature stretched bonds, greater electron delocalization, and flatter potential‐energy surfaces, making them more sensitive to self‐interaction error, basis‐set limitations, and the exact exchange–correlation balance of a functional. Because most functionals are parameterized primarily for equilibrium structures, error cancellation is more effective for minima than for saddle points. Consequently, larger deviations from high‐level reference data are commonly observed for transition states.^[^ ^85^ ^,^ ^86^ ^]^

We next assess the hybrid functionals B3LYP and PBE0. Relative to the GGA methods, both show markedly improved agreement with DLPNO‐CCSD(T), as evidenced by reduced deviations across the reaction profile. As shown in Figure 2A, B3LYP slightly underestimates **TS1** (signed error: −0.76 kcal mol^−1^) but more pronouncedly overestimates **TS2** (signed error: +3.19 kcal mol^−1^), placing these at +17.48 and +19.12 kcal mol^−1^ (relative to **A)**, respectively. As a result, **TS2** appears as the highest point on the B3LYP surface, leading this functional to predict migratory insertion as the on‐cycle RDS for **1a**. In contrast, PBE0 underestimates both barriers more substantially, with **TS1** and **TS2** deviating by −2.92 and −4.15 kcal mol^−1^, respectively, corresponding to free energy values of +15.32 and +11.78 kcal mol^−1^ above **A**. These results reinforce the tendency of PBE0 to compress the potential energy surface. The calculated local barriers for migratory insertion, $\text{Δ}G_{\mathrm{TS}2-C}^{‡}$, are +21.47 kcal mol^−1^ for B3LYP and +18.45 kcal mol^−1^ for PBE0, compared to +21.83 kcal mol^−1^ from DLPNO‐CCSD(T).

With regard to intermediates, B3LYP generally overestimates their relative energies, most notably for **D** (signed error: +6.82 kcal mol^−1^) and **F** (signed error: +4.51 kcal mol^−1^), contributing to a MAD of +3.31 kcal mol^−1^ and RMSD of +3.73 kcal mol^−1^. PBE0, by contrast, yields smaller deviations overall, with its lowest error for intermediate **B** (signed error: −0.53 kcal mol^−1^) and low deviations for **C** (signed error: −0.77 kcal mol^−1^) and **F** (signed error: −1.91 kcal mol^−1^). The largest discrepancy appears for intermediate **E** (signed error: −4.80 kcal mol^−1^), which leads PBE0 to incorrectly identify **E** rather than **C** as the resting state. Nevertheless, this overstabilization is counterbalanced by smaller errors elsewhere, resulting in improved statistical performance (MAD = +2.67 kcal mol^−1^, RMSD = +3.08 kcal mol^−1^). Altogether, both B3LYP and PBE0 provide a qualitatively accurate depiction of the catalytic mechanism, but PBE0 exhibits superior overall fidelity in reproducing DLPNO‐CCSD(T) energetics.

We now turn to the meta‐GGA Minnesota functionals M06 and M06‐L, which display markedly divergent behavior despite originating from the same functional family. As illustrated in Figure 2A, M06 moderately overestimates the Gibbs free energies across the profile, whereas M06‐L yields substantially inflated values and significant deviations across the entire reaction profile. For transition states, M06 overestimates **TS1** and **TS2** by +2.97 and +0.87 kcal mol^−1^, yielding global energy barriers of +21.21 and +16.80 kcal mol^−1^ relative to **A**, respectively—reasonably close to the DLPNO‐CCSD(T) reference values of +18.24 and +15.93 kcal mol^−1^. **TS3** is overestimated by +4.22 kcal mol^−1^ (ΔG = +3.81 kcal mol^−1^ versus ΔG = −0.41 kcal mol^−1^ at the coupled‐cluster reference). In contrast, M06‐L drastically overshoots all barriers, with signed errors of +4.83 kcal mol^−1^ (**TS1**), +9.53 kcal mol^−1^ (**TS2**), and +12.49 kcal mol^−1^ (**TS3**), corresponding to free energy values of +23.07, +25.46, and +12.08 kcal mol^−1^ above **A**, respectively, and clearly outside the typical range for chemically meaningful approximations.

Furthermore, both M06 and M06‐L incorrectly assign the C—H activation step as the local highest barrier, with $\text{Δ}G_{\mathrm{TS}1-A}^{‡}$ values of +21.21 and +23.07 kcal mol^−1^, compared to $\text{Δ}G_{\mathrm{TS}2-C}^{‡}$ values of +17.44 and +18.07 kcal mol^−1^, respectively. Although M06‐L is often recommended for organometallic thermochemistry,^[^ ^65^ ^,^ ^87^ ^]^ its local meta‐GGA form and lack of exact exchange leave self‐interaction error largely unmitigated—without compensating overbinding—which could lead to systematic energy overestimation, as seen here. Consistently, a recent broad benchmark on half‐sandwich [Cp*Rh] systems (Cp* = *η* ^5^‐pentamethylcyclopentadienyl), which are chemically akin to ours, also placed the Minnesota family among the weaker performers.^[^ ^88^ ^]^ Taken together, these points argue against using M06‐L for the present class of Ru(II) C—H alkenylation profiles.

Accordingly, the trend of overestimation also extends to the intermediates. For M06, deviations remain relatively contained, with signed errors of +2.97 kcal mol^−1^ for **B**, +5.26 kcal mol^−1^ for **C**, +3.90 kcal mol^−1^ for **D**, +1.69 kcal mol^−1^ for **E**, and +6.66 kcal mol^−1^ for **F**. These lead to an overall MAD of +3.57 kcal mol^−1^ and RMSD of +3.97 kcal mol^−1^. M06‐L, however, presents extreme overestimations for all intermediates, with signed errors ranging from +8.64 kcal mol^−1^ (**B**) to +14.70 kcal mol^−1^ (**F**), culminating in a MAD of +10.79 kcal mol^−1^ and RMSD of +11.20 kcal mol^−1^—the highest among all functionals considered. Notably, M06‐L erroneously identifies **C** (ΔG = +7.39 kcal mol^−1^) and **F** (ΔG = +11.64 kcal mol^−1^) as among the least stable intermediates, completely inverting their expected thermodynamic order.

In summary, while M06 moderately inflates the energy landscape and misrepresents the highest local energy barrier, it retains a qualitatively reasonable mechanistic profile, albeit less accurate than B3LYP or PBE0. By contrast, M06‐L exhibits pervasive and severe energetic inaccuracies, rendering it entirely unsuitable for mechanistic analysis in this system.

We proceed our functional benchmarking with the range‐separated hybrid *ω*B97X‐D3. Despite its design incorporating long‐range exact exchange, *ω*B97X‐D3 significantly overestimates the Gibbs free energies of all species involved in the catalytic cycle. As shown in Figure 2A, the transition state barriers are notably inflated: **TS1** is predicted at +21.48 kcal mol^−1^ (signed error: +3.24 kcal mol^−1^), and **TS2** at +22.21 kcal mol^−1^ (signed error: +6.28 kcal mol^−1^), relative to the DLPNO‐CCSD(T) reference of +18.24 and +15.93 kcal mol^−1^, respectively. This suggests that *ω*B97X‐D3 shifts the profile upward enough to distort relative barrier heights, leading to a misassignment of migratory insertion (**TS2**) as the global on‐cycle rate‐determining step.

Large discrepancies also extend to the intermediates. Notably, intermediate **C** is predicted to lie at +3.07 kcal mol^−1^ (signed error: +8.97 kcal mol^−1^), completely missing its role as the resting state (–5.90 kcal mol^−1^ at DLPNO‐CCSD(T)). This leads the resulting local barrier from **C** to **TS2** to be reduced to $\text{Δ}G_{\mathrm{TS}2-C}^{‡}$ = +19.14 kcal mol^−1^, now lower than that of the local C—H activation step ($\text{Δ}G_{\mathrm{TS}1-A}^{‡}$ = +21.48 kcal mol^−1^). Other intermediates are similarly destabilized, including **B** (signed error: +4.07 kcal mol^−1^), **D** (signed error: +7.51 kcal mol^−1^), **E** (signed error: +2.69 kcal mol^−1^), and **F** (signed error: +7.65 kcal mol^−1^). **TS3** is also significantly overpredicted at ΔG = +4.73 kcal mol^−1^ (+5.14 kcal mol^−1^ deviation). These accumulated errors lead to a MAD of +5.69 kcal mol^−1^ and RMSD of +6.08 kcal mol^−1^—among the highest observed in the benchmark set and substantially higher than those obtained with B3LYP or PBE0.

Taken together, these results highlight that *ω*B97X‐D3 performs poorly for this Ru(II)‐catalyzed C—H alkenylation system, failing to accurately capture both relative intermediate stabilities and transition‐state energetics. The pronounced destabilization of the expected resting state and distortion of relative barrier heights undermine its reliability for mechanistic modeling in this context. Caution is therefore advised when applying *ω*B97X‐D3 to similar catalytic processes.

Finally, we conclude our benchmark analysis with the double‐hybrid functionals B2PLYP and *ω*B2PLYP. These methods incorporate perturbative second‐order correlation and offer the potential for higher accuracy, particularly in capturing reaction energetics. As shown in Figure 2A, B2PLYP already performs reasonably well, with small deviations for the key transition states: **TS1** is predicted at +18.14 kcal mol^−1^ (signed error: −0.10 kcal mol^−1^) and **TS2** at +19.26 kcal mol^−1^ (signed error: +3.33 kcal mol^−1^), both relative to the DLPNO‐CCSD(T) references of +18.24 and +15.93 kcal mol^−1^. However, several intermediates are significantly overestimated, particularly **D** (signed error: +7.14 kcal mol^−1^), **E** (signed error: +4.08 kcal mol^−1^), and **F** (signed error: +4.35 kcal mol^−1^), resulting in a moderate statistical error (MAD = +3.27 kcal mol^−1^; RMSD = +3.84 kcal mol^−1^).

In contrast, *ω*B2PLYP—its range‐separated analog—offers a marked improvement across the entire profile. The transition states **TS1** and **TS2** are nearly spot‐on at +18.25 kcal mol^−1^ (signed error: +0.01 kcal mol^−1^) and +16.53 kcal mol^−1^ (signed error: +0.60 kcal mol^−1^), respectively. All intermediate energies are brought significantly closer to the reference, with the largest deviation reduced to +2.02 kcal mol^−1^ for intermediate **C** (ΔG = −3.88 kcal mol^−1^ versus −5.90 kcal mol^−1^ at CCSD(T)). Intermediate **E** is even slightly underestimated (signed error: −1.74 kcal mol^−1^), and **TS3** is accurately recovered at ΔG = −1.54 kcal mol^−1^ (signed error: −1.13 kcal mol^−1^). The improvement is also reflected in the statistical metrics, with *ω*B2PLYP achieving the lowest MAD (+0.99 kcal mol^−1^) and RMSD (+1.16 kcal mol^−1^) among all tested functionals. Also, both methods capture that the migratory insertion presents the largest local barrier, whereas C—H activation is the on‐cycle rate‐determining step, in agreement with the coupled‐cluster data.

Overall, the inclusion of range separation in *ω*B2PLYP significantly enhances the accuracy of the double‐hybrid approach, yielding excellent agreement with DLPNO‐CCSD(T) across both transition states and intermediates. For context, PBE0 is the best‐performing hybrid and second‐best overall, but its average RMSD (≈3.08 kcal mol^−1^) is driven mainly by a systematic underestimation of transition states, which compresses the computed barriers. These results establish *ω*B2PLYP as the most reliable method among those benchmarked for modeling the mechanism of Ru(II)‐catalyzed C—H alkenylation of naphthoquinones.

### 3.3 Substituent Effects on Reaction Energetics

**Figure 3:**
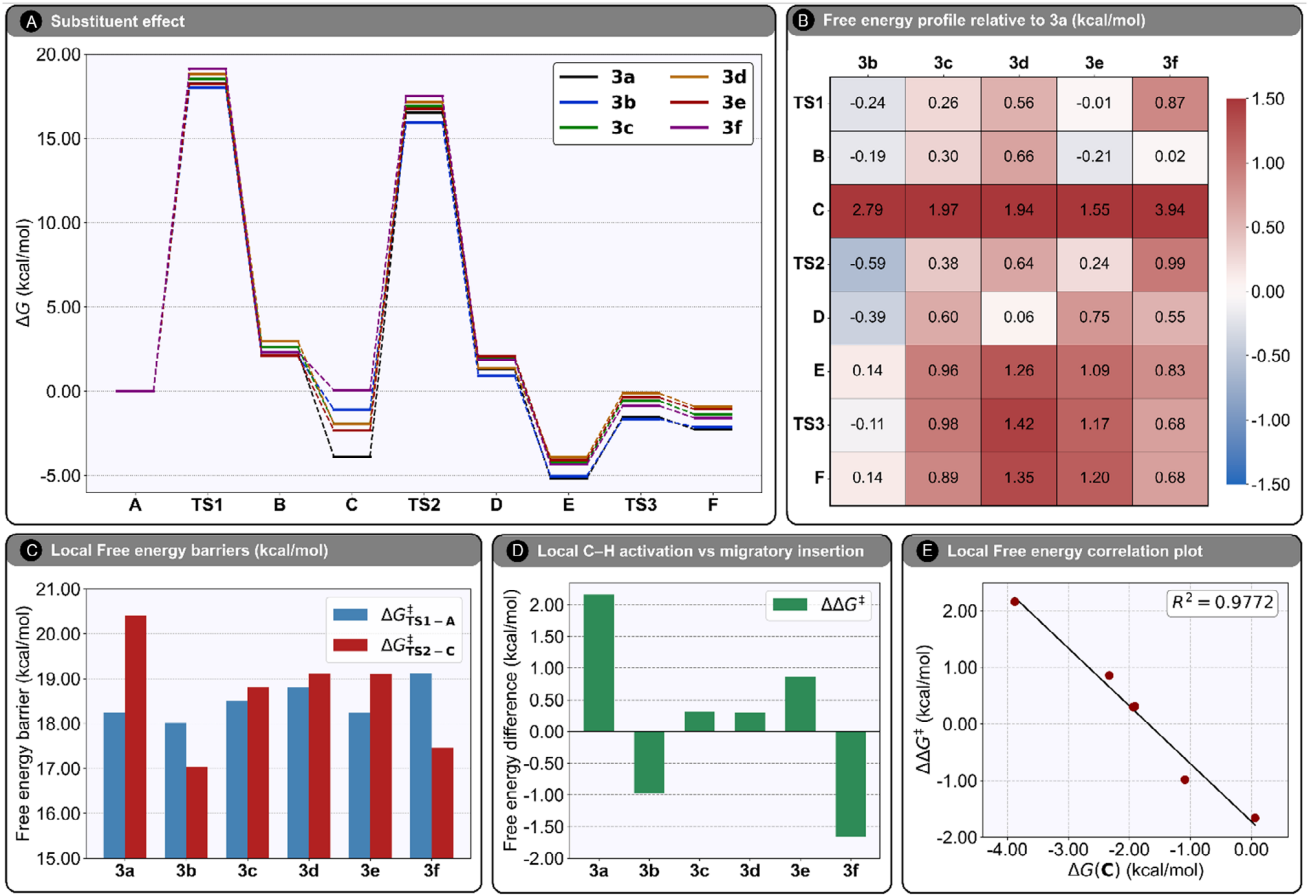
A) Computed Gibbs free energies (in kcal mol^−1^) for the C—H alkenylation according to the variation of the substituent present in the B‐ring of 1,4‐naphthoquinone. Energies are at the *ω*B2PLYP‐D3/def2‐TZVPP+CPCM(DCE) level, from optimized structures at PBE0‐D3(BJ)/bs1+CPCM(DCE). B) Heat map of Gibbs free energy differences (in kcal mol^−1^) for intermediates and transition states involved in the C—H alkenylation mechanism of derivatives **3b**–**3f**, relative to **3a**. C) Local free energy barriers (in kcal mol^−1^) for the C—H activation and olefin insertion steps, as a function of the substituent on the B‐ring of 1,4‐naphthoquinone. D) Local free energy difference $\left(\text{Δ}\text{Δ}G^{‡}=\text{Δ}G_{TS2-C}^{‡}-\text{Δ}G_{TS1-A}^{‡}\right)$ as a function of the B‐ring substituent. E) Correlation between the energy difference described in (D) and the computed Gibbs free energy of intermediate **C**.

In this section, we examine how substitution at the B‐ring of 1,4‐naphthoquinone influences the reaction energetics of the Ru(II)‐catalyzed C—H alkenylation with ethenesulfonyl fluoride (**2**). In addition to the parent compound menadione (**1a**), five experimentally accessible derivatives were considered: 2,3‐dimethyl‐1,4‐naphthoquinone (**1b**), 2‐methoxy‐1,4‐naphthoquinone (**1c**), 2,3‐dimethoxy‐1,4‐naphthoquinone (**1d**), 2‐chloro‐3‐methoxy‐1,4‐naphthoquinone (**1e**), and 2‐chloro‐1,4‐naphthoquinone (**1f**). The substituents comprise primarily electron‐donating groups, which are generally expected to promote C—H activation at the A‐ring. Their selection was guided by synthetic accessibility, and the associated free energy profiles and energetic differences are summarized in **Figure** **3**. All final energies were obtained from single‐point calculations using the *ω*B2PLYP functional, identified as the most reliable density functional from our benchmarking study.

Figure 3A presents full free energy profiles for all six substrates (**A** → **F**), each referenced to its reactant complex **A**. Figure 3B reports per‐stationary‐point energies relative to the parent system (**3a**), enabling a direct view of substituent effects. Figure 3C compiles local (versus immediate precursor) activation free energies for C—H activation and migratory insertion: $\text{Δ}G_{\mathrm{TS}1-A}^{‡}$ and $\text{Δ}G_{\mathrm{TS}2-C}^{‡}$. Figure 3D gives the corresponding energy difference, $\text{Δ}\text{Δ}G^{‡}(\text{local})=\text{Δ}G_{\mathrm{TS}2-C}^{‡}-\text{Δ}G_{\mathrm{TS}1-A}^{‡}$. A positive $\text{Δ}\text{Δ}G^{‡}$ indicates migratory insertion is the local on‐cycle highest energy barrier; a negative value indicates C—H activation is locally higher. Similar values considering global energies are found in Figure S8, Supporting Information. Finally, Figure 3E correlates $\text{Δ}\text{Δ}G^{‡}(\text{local})$ with the relative stability of intermediate **C**. Table S14–S19, Supporting Information, summarize the underlying energy data.

All six derivatives follow the same mechanistic blueprint, but they differ markedly in the stability of intermediate **C** and in both the magnitude and ordering of the local activation barriers. As the heatmap in Figure 3B shows, **C** varies the most across the series and drives the largest deviations from the **3a** reference. Most stationary points in **3b** are stabilized relative to **3a**—most notably **TS2** (–0.59 kcal mol^−1^), **D** (–0.39 kcal mol^−1^), and **TS1** (–0.24 kcal mol^−1^)—yielding an overall more favorable landscape. Locally, **3a** places **TS2** +2.16 kcal mol^−1^ above **TS1** (migratory insertion > C—H activation), whereas **3b** reverses this, with **TS1** +0.98 kcal mol^−1^ above **TS2** (C—H activation > migratory insertion). Across all substrates, however, C—H activation remains the highest global on‐cycle barrier and thus the on‐cycle rate‐determining step.

For the methoxy cases **3c** and **3d**, **TS1** and **TS2** are nearly isoenergetic locally ($\text{Δ}\text{Δ}G^{‡}(\text{local})$ = +0.31 and +0.30 kcal mol^−1^, respectively). Despite methoxy being a stronger donor than methyl, neither substrate shows a decisive local preference for C—H activation or insertion. In contrast, **3e** (2‐chloro‐3‐methoxy) favors insertion as the higher local hurdle ($\text{Δ}\text{Δ}G^{‡}(\text{local})$ = +0.86 kcal mol^−1^), while **3f** (chloro only) shows the opposite trend, with **TS1** higher than **TS2** by +1.67 kcal mol^−1^.

These variations cannot be rationalized solely by generic “donor strength” at the B‐ring. Instead, Figure 3E reveals a simple organizing principle: the local ordering tracks the thermodynamics of **C**. Stabilizing **C** elevates the local insertion barrier relative to C—H activation; destabilizing **C** has the opposite effect. This tuning of **C** reorders local barriers without overturning the global picture, that is, **TS1** remains highest for all substrates. Consistent with this, we find no meaningful correlation between the energy of **C** and $\text{Δ}\text{Δ}G^{‡}(\text{local})$ (Figure S8, Supporting Information). Practically, modulating the stability of **C** offers a handle to bias the local kinetics of insertion vs C—H activation, even if the global on‐cycle RDS does not change within this series.

Although migratory insertion is not the global on‐cycle barrier in our naphthoquinone series, precedents show it can become turnover‐limiting in closely related manifolds. For Ru(II) systems, a decarboxylative C—H alkenylation of aryl carboxylic acids with alkynes identified insertion transition states as decisive for chemoselectivity and, under certain conditions, as the highest barrier.^[^ ^49^, ^50^, ^51^ ^]^ Enantioselective Ru/CICA (chiral imidazolidine carboxylic acid) C—H functionalization likewise implicated migratory insertion as both stereocontrolling and turnover‐limiting, contingent on the ligand environment.^[^ ^89^ ^]^ Outside Ru, migratory insertion‐limited cycles are documented for Rh,^[^ ^90^ ^]^ Pd,^[^ ^91^ ^]^ and Co platforms,^[^ ^92^ ^,^ ^93^ ^]^ and broader comparisons of Ru‐catalyzed allylation/alkenylation/hydroarylation show that the rate‐determining state can shift across closely related conditions.^[^ ^52^ ^]^ Together, these studies underscore that migratory insertion is highly sensitive to ligand, coupling partner, and acid/base environment and can assume global rate control. While our substitutions do not achieve that regime, they do reveal substantial local sensitivity at insertion, suggesting that with further tuning (ligand sphere, additives, or coupling partner), a migratory‐insertion‐limited pathway may still be accessible for naphthoquinones.

### 3.4 Evaluating Low‐Cost Optimization Strategies for Mechanistic Exploration

**Figure 4:**
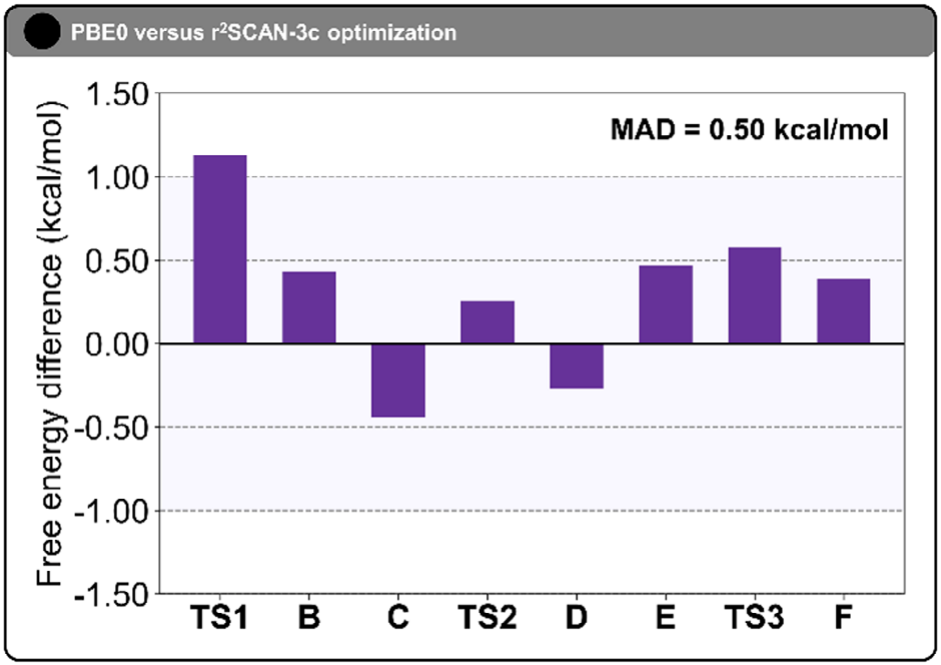
Free energy differences (in kcal mol^−1^) between DLPNO‐CCSD(T)//r^2^SCAN‐3c and DLPNO‐CCSD(T)//PBE0‐D3(BJ) for all species along the reaction pathway. Free energies include thermal corrections and solvent effects and were computed with the same basis sets and solvation model used throughout this study. A positive energy difference indicates that the r^2^SCAN‐3c geometry yields a higher energy than the corresponding PBE0‐optimized structure. The shaded area highlights the sub‐kcal mol^−1^ region (±1.00 kcal mol^−1^). The mean absolute deviation (MAD) across the entire mechanism is +0.50 kcal mol^−1^.

Lastly, we assess whether the use of lower‐cost computational methods for geometry optimization can reliably reproduce the energetic features of the full reaction mechanism. Specifically, we compare geometries optimized with the r^2^SCAN‐3c composite method to those obtained at the PBE0‐D3(BJ) level, using DLPNO‐CCSD(T) single‐point energies in both cases for consistent energetic evaluation. All calculations employ the same basis sets and solvent corrections as described previously, ensuring a consistent comparison across the dataset. The free energy differences (in kcal mol^−1^) between DLPNO‐CCSD(T)//r^2^SCAN‐3c and DLPNO‐CCSD(T)//PBE0‐D3(BJ) for all species are summarized in **Figure** **4** and Table S20, Supporting Information, while the full energy profiles derived from both methods are provided in Figure S9, Supporting Information.

As shown in Figure 4, the largest deviation occurs for **TS1**, where the activation barrier is overestimated by +1.13 kcal mol^−1^ when the r^2^SCAN‐3c geometry is used instead of the PBE0‐optimized structure ($\text{Δ}G_{TS1-A}^{‡}$ = +19.37 kcal mol^−1^ versus +18.24 kcal mol^−1^). This difference is consistent with the more substantial structural distortion at the transition state level: the RMSD between r^2^SCAN‐3c and PBE0 geometries is 0.52 Å for **TS1**, whereas it is only 0.10 Å for **A** and 0.06 Å for **B**. Intermediate **B** also shows a modest energy difference of + 0.43 kcal mol^−1^ between r^2^SCAN‐3c and PBE0. Across the full mechanism, the signed errors range from −0.44 kcal mol^−1^ to +1.13 kcal mol^−1^, with a mean absolute deviation (MAD) of +0.50 kcal mol^−1^.

Despite these variations, the use of r^2^SCAN‐3c offers a considerable computational advantage. Geometry optimizations converge up to seven times faster than with PBE0 under identical settings, enabling efficient exploration of reaction mechanisms.

Importantly, using r^2^SCAN‐3c for geometry optimization does not change the mechanistic picture. The local migratory‐insertion barrier (**TS2** relative to **C**) is +22.53 kcal mol^−1^ at DLPNO‐CCSD(T)//r^2^SCAN‐3c, only +0.70 kcal mol^−1^ higher than the DLPNO‐CCSD(T)//PBE0‐D3(BJ) value of +21.83 kcal mol^−1^. In both cases, migratory insertion is locally higher than C—H activation (**TS1** relative to **A**). At the same time, the global highest on‐cycle barrier remains C—H activation: for the r^2^SCAN‐3c geometries, $\text{Δ}G_{\mathrm{TS}1-A}^{‡}$ = +19.37 kcal mol^−1^ versus $\text{Δ}G_{\mathrm{TS}2-A}^{‡}$ = +16.19 kcal mol^−1^. Thus, C—H activation is still the on‐cycle rate‐determining step. These results support r^2^SCAN‐3c as a low‐cost yet qualitatively reliable option for screening reaction energetics in ruthenium‐catalyzed C—H functionalization pathways.

## 4 Conclusion

In this work, we present a comprehensive computational investigation of the Ru(II)‐catalyzed C—H alkenylation of naphthoquinones with ethenesulfonyl fluoride, combining high‐level DLPNO‐CCSD(T) benchmarks with an extensive assessment of widely used DFT functionals. Our benchmarking study revealed that, among nine tested functionals, the range‐separated double‐hybrid *ω*B2PLYP most accurately reproduces DLPNO‐CCSD(T) free energies for both intermediates and transition states—achieving sub−1.00 kcal mol^−1^ deviations across almost the entire catalytic cycle. In contrast, pure GGA functionals (BP86, PBE) systematically compress the energy landscape, hybrid meta‐GGAs (M06, M06‐L) and *ω*B97X‐D3 show inconsistent errors or large overestimations, and even popular hybrids (B3LYP, PBE0) underpredict key barriers by several kcal mol^−1^. These findings establish *ω*B2PLYP as the most reliable DFT level for studying Ru(II)‐catalyzed C—H alkenylation, providing a validated computational framework for subsequent mechanism‐driven design.

Using *ω*B2PLYP single‐point energies, we then examined how B‐ring substitution modulates each elementary step. All six derivatives (**3a**–**3f**) follow the same pathway, and C—H activation remains the highest global on‐cycle barrier (i.e., the on‐cycle rate‐determining step) for every substrate. However, the local ordering can vary: the stability of intermediate **C** tunes the local migratory‐insertion barrier (**TS2** relative to **C**). Mapping the local energy barrier differences ($\text{Δ}\text{Δ}G^{‡}(\text{local})$) against the free energy of **C** reveals a clear correlation—less stabilization of **C** lowers the local insertion barrier relative to C—H activation. By contrast, no meaningful correlation is observed for the global $\text{Δ}\text{Δ}G^{‡}$. Thus, while the global rate‐determining step does not change within **3a**–**3f**, intermediate‐**C** stabilization offers a practical lever to adjust local barrier heights. Because the computed on‐cycle barriers are modest, these results are also consistent with a scenario in which off‐cycle formation of the active cationic Ru species controls the overall rate, while within the catalytic cycle C—H activation remains the highest global barrier.

Finally, we evaluated the feasibility of replacing full DFT optimizations with the lower‐cost r^2^SCAN‐3c method. The largest deviation, +1.13 kcal mol^−1^ for **TS1**, remains within acceptable limits, with all other errors ≤0.60 kcal mol^−1^ and a mean absolute deviation of +0.50 kcal mol^−1^. Crucially, the relative global and local ordering of key barriers is preserved, and r^2^SCAN‐3c optimizations are significantly faster than PBE0. These results support r^2^SCAN‐3c as a reliable and efficient choice for rapid mechanistic screening in Ru(II)‐catalyzed C—H functionalization.

Collectively, these findings i) establish *ω*B2PLYP as a workhorse for accurate energetics in Ru(II) C—H alkenylation; ii) connect intermediate‐**C** stability to local barrier modulation between C—H activation and migratory insertion—offering a concrete handle for substituent‐guided design; and iii) demonstrate that r^2^SCAN‐3c can markedly reduce computational cost without sacrificing the mechanistic conclusions. We anticipate that this protocol will aid the rational development of sulfonyl‐fluoride–functionalized quinones for SuFEx‐enabled medicinal chemistry, including applications to neglected‐disease therapeutics.

## Conflict of Interest

The authors declare no conflict of interest.

## Supporting information

Supplementary Material

## Data Availability

The data that support the findings of this study are available in the supplementary material of this article.
