# Supplementary material for: Ruthenium‐Catalyzed C—H Alkenylation of Trypanocidal Naphthoquinones: A Mechanistic Benchmarking Study
Source: ChemistryOpen. 2025 Nov 5;15(2):e202500465. doi: 10.1002/open.202500465 (PMC12927953; doi:10.1002/open.202500465)
Supplement: Supplementary file 1 — Supplementary Material [file OPEN-15-e202500465-s001.pdf]

Supplementary Information for

Ruthenium-Catalyzed C–H Alkenylation of Trypanocidal Naphthoquinones: A Mechanistic Benchmarking Study

Esther R. S. Paz,[a] Cauê P. Souza,[b] Joyce C. De Oliveira,[a] Renata G. Almeida,[a] Chonny Herrera-Acevedo,[c] Sulaiman Lakoh,[d] Guilherme A. M. Jardim,*[a] Eufrânio N. da Silva Júnior,*[a] and Felipe Fantuzzi*[b]

[a]Instituto de Ciências Exatas, Departamento de Química, Universidade Federal de Minas Gerais, Belo Horizonte, MG 31270–901, Brazil. [b]Chemistry and Forensic Science, School of Natural Sciences, University of Kent, Park Wood Rd, Canterbury CT2 7NH, United Kingdom. [c]Department of Chemical Engineering, Universidad ECCI, Carrera 19 # 49–20, 111311 Bogotá D.C., Colombia. [d]Department of Internal Medicine, College of Medicine and Allied Health Sciences, University of Sierra Leone, Freetown, Sierra Leone

E-mails:

guilhermeamj@ufmg.br (GAMJ)

eufranio@ufmg.br (ENSJ)

f.fantuzzi@kent.ac.uk (FF)

Table of Contents

DFT mechanistic data for compound 1a S3

DFT mechanistic data for compound 1b S32

DFT mechanistic data for compound 1c S49

DFT mechanistic data for compound 1d S66

DFT mechanistic data for compound 1e S84

DFT mechanistic data for compound 1f S101

Comparison of global and local energy barriers S117

r^2SCAN-3c mechanistic data for compound 1a S118

DFT mechanistic data for compound 1a

Table S1. Computed free energies (G_{tot}) of all species involved in the C–H alkenylation reaction pathway of menadione (**1a**). G_{tot} was obtained as the sum of the thermal correction to Gibbs free energy (G_{corr}) at the PBE0-D3(BJ)/bs1+CPCM(DCE) level; the electronic energy (E) at the PBE0-D3(BJ)/def2-TZVPP+CPCM(DCE) level; and the concentration correction (G_{conc}). Herein, bs1 = def2-TZVP for Ru, def2-SVP for other elements; DCE = 1,2-dichloroethane.

Species	G_{corr} (Eh)	E (Eh)	G_{conc} (Eh)	G_{tot} (Eh)
**AcOH**	0.035039	-228.946441	0.003012	-228.908390
**$C_2H_3SO_2F$**	0.025247	-726.152013	0.003012	-726.123755
**A**	0.377204	-1286.402372	0.003012	-1286.022156
**TS1**	0.373144	-1286.373892	0.003012	-1285.997736
**B**	0.378125	-1286.401600	0.003012	-1286.020463
**C**	0.372984	-1783.624140	0.003012	-1783.248143
**TS2**	0.372895	-1783.594660	0.003012	-1783.218752
**D**	0.374349	-1783.617323	0.003012	-1783.239962
**E**	0.372975	-1783.626618	0.003012	-1783.250632
**TS3**	0.370281	-1783.618356	0.003012	-1783.245064
**F**	0.371283	-1783.619741	0.003012	-1783.245446

Cartesian Coordinates for the Reaction Pathway of Compound 1a

All values are in Ångstrom

AcOH

H	0.535739	-0.008153	-2.653144
O	0.535743	-0.494303	-1.811370
O	0.535743	1.594731	-1.029815
C	0.535751	0.404673	-0.823347
C	0.535744	-0.243430	0.525903
H	1.421457	-0.888024	0.627310
H	0.535741	0.522529	1.308784
H	-0.349969	-0.888024	0.627303

$C_2H_3SO_2F$

C	-1.148281	-1.327974	-1.152267

C	-0.569929	-0.158549	-1.409948
H	0.396424	-0.005222	-1.897992
H	-2.119013	-1.385254	-0.651904
H	-0.652938	-2.259832	-1.437821
S	-1.348911	1.321627	-0.908869
O	-2.601467	1.067464	-0.237289
O	-0.379698	2.251473	-0.379569
F	-1.740750	1.903609	-2.360824

**A**			
C	-1.727683	0.323938	-0.374957
C	-2.620708	1.433818	-0.687745
C	-3.934474	1.432095	-0.372761
C	-4.528433	0.236402	0.300673
C	-3.658401	-0.952250	0.505264
C	-2.294140	-0.925306	0.151909
C	-1.522248	-2.082001	0.288006
C	-2.092049	-3.245090	0.802166
C	-3.433190	-3.259505	1.184498
C	-4.216154	-2.115682	1.030030
C	-4.846304	2.572761	-0.637124
O	-0.515132	0.508965	-0.564516
O	-5.692536	0.244424	0.648984
H	-2.149086	2.303069	-1.153054
H	-0.482422	-2.081824	-0.039231
H	-1.483561	-4.146806	0.899764
H	-3.876180	-4.170835	1.592494
H	-5.272913	-2.112403	1.303932
H	-5.682614	2.245940	-1.275665
H	-5.298501	2.922940	0.304144
H	-4.320378	3.404056	-1.122338
Ru	1.411872	-0.117331	-0.102132
O	1.700668	0.363742	-2.146063

O	1.128978	-1.627096	-1.567333
C	1.369291	-0.804588	-2.505472
C	1.288129	-1.200874	-3.935034
H	0.501535	-1.953023	-4.076103
H	2.250220	-1.650903	-4.227112
H	1.107651	-0.322978	-4.567208
C	1.612617	-2.725575	1.951122
C	1.966384	-1.309956	1.635907
C	1.155320	-0.228578	2.044398
C	1.468798	1.113381	1.671903
C	2.593508	1.410147	0.873551
C	3.420996	0.311619	0.463267
C	3.113111	-1.013351	0.827656
C	2.903860	2.798641	0.372224
C	3.954945	3.429561	1.288521
C	1.674288	3.683571	0.220766
H	1.956273	4.627382	-0.268092
H	0.900562	3.198462	-0.394117
H	1.231804	3.940266	1.195937
H	3.356536	2.667155	-0.625097
H	4.249919	4.415927	0.900572
H	4.858546	2.805346	1.358361
H	3.552727	3.566601	2.304741
H	0.750987	1.900137	1.906687
H	0.218019	-0.431909	2.566517
H	4.232511	0.494430	-0.244958
H	3.694406	-1.834444	0.402880
H	1.757226	-3.371876	1.073904
H	0.577560	-2.811688	2.304519
H	2.281336	-3.091436	2.747433

TS1 (imaginary frequency: -643.22 cm^{-1})

C	-2.133241	0.509333	-0.216073

C	-3.425962	1.161773	-0.251057
C	-4.563236	0.463861	-0.016580
C	-4.507560	-1.014655	0.255588
C	-3.184686	-1.691030	0.180699
C	-2.042736	-0.934767	-0.100177
C	-0.766352	-1.518061	-0.240183
C	-0.679180	-2.910549	-0.074428
C	-1.807036	-3.667365	0.243845
C	-3.062426	-3.066730	0.366855
C	-5.910472	1.084581	0.007350
O	-1.070743	1.167046	-0.248945
O	-5.524058	-1.627228	0.510694
H	-3.440369	2.241131	-0.420489
H	-0.017970	-1.210820	-1.223492
H	0.278663	-3.413813	-0.228047
H	-1.709877	-4.747372	0.376283
H	-3.952459	-3.656445	0.594856
H	-5.859497	2.162181	-0.190834
H	-6.558186	0.603392	-0.743006
H	-6.391793	0.911422	0.982744
Ru	0.697555	0.098941	0.127716
O	1.137329	0.673196	-1.798461
O	0.400306	-1.243771	-2.618960
C	0.946705	-0.134041	-2.768252
C	1.454390	0.297832	-4.109923
H	1.376983	1.386673	-4.219514
H	0.913705	-0.217960	-4.912049
H	2.519840	0.024797	-4.172516
C	-0.169698	-1.371482	3.057599
C	0.802874	-0.591419	2.236397
C	0.748266	0.815335	2.169879
C	1.679443	1.572312	1.392093
C	2.687814	0.928027	0.644173
C	2.710026	-0.502481	0.645349

C	1.788512	-1.235274	1.421073
C	3.657808	1.667558	-0.239413
C	5.036823	1.637133	0.427360
C	3.227792	3.083977	-0.587043
H	3.935730	3.515905	-1.309189
H	2.226812	3.097767	-1.042018
H	3.222499	3.738432	0.298825
H	3.713009	1.085240	-1.174213
H	5.779263	2.112535	-0.230474
H	5.370530	0.608413	0.630261
H	5.021718	2.187347	1.381266
H	1.556235	2.653201	1.328931
H	-0.070392	1.338488	2.669714
H	3.404861	-1.033826	-0.007685
H	1.788183	-2.324630	1.347580
H	-0.352542	-2.364620	2.627650
H	-1.125401	-0.837589	3.148697
H	0.242199	-1.511900	4.069540

**B**			
C	-1.686595	-0.068203	-1.210042
C	-2.778503	-0.479010	-2.071287
C	-4.062684	-0.400660	-1.646844
C	-4.384752	0.112530	-0.268241
C	-3.255439	0.523472	0.605034
C	-1.942002	0.406845	0.126933
C	-0.798156	0.744522	0.877645
C	-1.046059	1.240005	2.171554
C	-2.347811	1.379804	2.658185
C	-3.460063	1.019349	1.887673
C	-5.222549	-0.799149	-2.483961
O	-0.494541	-0.099787	-1.596916
O	-5.541952	0.179459	0.099006

H	-2.527682	-0.842301	-3.070984
H	0.850655	-0.601697	2.283308
H	-0.217303	1.523486	2.827218
H	-2.500730	1.774881	3.665849
H	-4.475772	1.120986	2.274594
H	-4.902653	-1.151404	-3.472368
H	-5.797228	-1.592956	-1.980751
H	-5.913923	0.050090	-2.602761
Ru	0.929953	0.373413	-0.139618
O	0.618511	-1.671796	0.288269
O	0.746066	-1.550621	2.505103
C	0.586617	-2.217168	1.394949
C	0.354504	-3.673226	1.550883
H	0.345979	-4.162890	0.572050
H	-0.612845	-3.821040	2.055393
H	1.133240	-4.103112	2.196667
C	0.796466	3.684944	0.205065
C	1.588895	2.443860	-0.043039
C	1.774572	1.925917	-1.348957
C	2.588012	0.776127	-1.613532
C	3.150203	0.050275	-0.559448
C	2.887973	0.515136	0.777686
C	2.168742	1.700734	1.033573
C	3.935646	-1.223303	-0.749833
C	5.430605	-0.903229	-0.665089
C	3.588338	-1.973496	-2.027438
H	4.112162	-2.940283	-2.044624
H	2.507292	-2.167427	-2.097553
H	3.900567	-1.417370	-2.925167
H	3.679456	-1.869822	0.107080
H	6.021269	-1.829441	-0.726953
H	5.684735	-0.397991	0.279012
H	5.733243	-0.246882	-1.496298
H	2.675127	0.419227	-2.639988

H	1.256750	2.408203	-2.180844
H	3.251415	-0.075802	1.622410
H	1.996751	2.013422	2.065417
H	0.267394	3.635397	1.165950
H	0.065196	3.854348	-0.596781
H	1.476806	4.550946	0.236777

**C**			
C	1.641508	-0.634397	0.746829
C	3.071030	-0.792737	0.897288
C	3.578186	-1.922486	1.449751
C	2.667831	-3.029399	1.920934
C	1.203648	-2.852439	1.742704
C	0.734549	-1.662167	1.165997
C	-0.626106	-1.384332	0.950660
C	-1.535478	-2.376810	1.351174
C	-1.083770	-3.568114	1.919641
C	0.281671	-3.817526	2.123164
C	5.031705	-2.156094	1.632210
O	1.134539	0.401013	0.242138
O	3.141307	-4.026921	2.427150
H	3.716210	0.019167	0.553721
H	-2.610740	-2.226725	1.226920
H	-1.814507	-4.324262	2.217461
H	0.624988	-4.750953	2.573116
H	5.254706	-2.333848	2.696254
H	5.335725	-3.072139	1.100806
H	5.624875	-1.308041	1.269224
R	-0.932058	0.403808	0.043358
C	-4.230534	-0.364833	-0.541747
C	-2.908934	0.129668	-1.026941
C	-1.957755	-0.732221	-1.621839
C	-0.717705	-0.229027	-2.060652

C	-0.435415	1.180180	-2.074727
C	-1.399735	2.050233	-1.541193
C	-2.571736	1.516024	-0.954681
C	0.886161	1.649164	-2.620461
C	1.279442	3.046643	-2.167824
C	0.850875	1.534266	-4.148735
H	1.831793	1.811075	-4.562014
H	0.617232	0.511326	-4.479591
H	0.095702	2.214827	-4.571938
H	1.636105	0.933678	-2.242759
H	2.302953	3.266855	-2.503520
H	1.246161	3.146996	-1.073439
H	0.619496	3.812849	-2.604227
H	0.044949	-0.928164	-2.410692
H	-2.142366	-1.808222	-1.647968
H	-1.221641	3.124199	-1.494670
H	-3.259060	2.198819	-0.452060
H	-4.966761	-0.247789	-1.353109
H	-4.190564	-1.427986	-0.273044
H	-4.588091	0.217531	0.317273
S	-0.801204	3.212471	1.672978
C	-0.824062	1.474169	1.856419
C	-2.045996	0.772597	1.858300
H	-2.973334	1.313816	1.656226
H	-2.143033	-0.109906	2.493475
H	0.029297	1.154030	2.464255
O	0.504237	3.682638	1.269026
O	-2.003086	3.720177	1.046219
F	-0.934156	3.621068	3.229328

TS2 (imaginary frequency: -262.30 cm^{-1})

C	1.714146	-0.945080	0.873205
C	3.022163	-1.530488	1.076391

C	3.160470	-2.686824	1.769827
C	1.961007	-3.363526	2.376553
C	0.636932	-2.699207	2.237251
C	0.555263	-1.497736	1.524890
C	-0.650737	-0.766386	1.400243
C	-1.795867	-1.327124	1.995511
C	-1.726405	-2.545352	2.663563
C	-0.514996	-3.234086	2.804600
C	4.463214	-3.369892	1.963959
O	1.540428	0.036250	0.108432
O	2.090304	-4.413930	2.971984
H	3.876881	-1.036877	0.607657
H	-2.743371	-0.783841	1.959499
H	-2.635316	-2.959212	3.106485
H	-0.459057	-4.174266	3.356054
H	4.674645	-3.480206	3.039556
H	4.418566	-4.391834	1.555278
H	5.281510	-2.819004	1.484554
Ru	-0.463253	0.467696	-0.337285
C	-2.870819	-2.021656	-0.839278
C	-2.050699	-0.873371	-1.312812
C	-0.791463	-1.084200	-1.914147
C	-0.054304	0.009051	-2.439449
C	-0.544383	1.334147	-2.427132
C	-1.764188	1.546374	-1.719770
C	-2.496619	0.472744	-1.159493
C	0.239125	2.434926	-3.114778
C	1.515742	2.795152	-2.355542
C	-0.607687	3.665245	-3.416763
H	-0.034729	4.356483	-4.051916
H	-1.531445	3.401610	-3.954159
H	-0.876259	4.204191	-2.497399
H	0.541480	1.991532	-4.079619
H	2.112863	3.507926	-2.943462

H	2.137889	1.909329	-2.160561
H	1.266632	3.266156	-1.394185
H	0.942917	-0.179125	-2.845239
H	-0.362021	-2.087165	-1.944202
H	-2.155110	2.556359	-1.599923
H	-3.418390	0.680439	-0.613931
H	-3.427679	-2.410846	-1.707853
H	-2.244998	-2.834833	-0.449909
H	-3.603100	-1.718963	-0.081597
S	-1.118802	3.466858	0.814467
C	-0.101349	2.059743	0.942760
C	-0.588997	1.035266	1.857922
H	-1.612583	1.176249	2.217538
H	0.111314	0.803409	2.666120
H	0.944824	2.377870	1.014380
O	-0.598053	4.431354	-0.129154
O	-2.528317	3.131275	0.832345
F	-0.837318	4.117394	2.268535

**D**			
C	1.585150	-0.320262	1.045564
C	2.653043	-1.247802	0.707521
C	2.585773	-2.570310	0.977546
C	1.347275	-3.137898	1.587125
C	0.263637	-2.200441	1.977901
C	0.406693	-0.794668	1.783272
C	-0.604248	0.095112	2.254200
C	-1.780424	-0.478500	2.807491
C	-1.918478	-1.840506	2.957690
C	-0.878399	-2.703173	2.566990
C	3.673069	-3.529568	0.662265
O	1.652639	0.894555	0.688086
O	1.236914	-4.338773	1.750090

H	3.523599	-0.806084	0.216028
H	-2.564333	0.197190	3.153624
H	-2.823775	-2.251007	3.409578
H	-0.956365	-3.781696	2.718256
H	3.973302	-4.070061	1.573595
H	3.309554	-4.294887	-0.041499
H	4.545366	-3.022369	0.232731
Ru	-0.255214	0.375166	-0.157392
C	-2.137359	-2.431931	-0.565531
C	-1.409864	-1.266274	-1.140746
C	-0.066884	-1.377605	-1.587990
C	0.614390	-0.244122	-2.065715
C	-0.044141	1.013992	-2.263237
C	-1.410296	1.083705	-1.898751
C	-2.069015	-0.009615	-1.288527
C	0.701908	2.177638	-2.879933
C	1.924674	2.587888	-2.061786
C	-0.190757	3.375928	-3.169874
H	0.394647	4.153565	-3.680777
H	-1.037398	3.111599	-3.821212
H	-0.590843	3.811198	-2.241022
H	1.063557	1.778305	-3.845622
H	2.501791	3.342487	-2.615126
H	2.590447	1.741261	-1.844653
H	1.613813	3.027775	-1.102629
H	1.684108	-0.322378	-2.276112
H	0.473354	-2.317407	-1.455625
H	-1.939697	2.032286	-1.977635
H	-3.091497	0.108006	-0.925536
H	-2.756846	-2.871085	-1.364843
H	-1.451271	-3.207240	-0.204442
H	-2.813111	-2.123769	0.242986
S	-2.251060	2.791284	0.829815
C	-0.641237	2.126983	0.959903

C	-0.401953	1.591908	2.362509
H	-1.075698	2.007831	3.124366
H	0.622863	1.822297	2.676732
H	0.032111	2.930325	0.632163
O	-2.469956	3.531435	-0.397492
O	-3.266258	1.876521	1.315666
F	-2.146352	3.941445	1.973842

**E**			
C	1.092335	-1.151024	1.115097
C	2.449068	-1.618374	1.366411
C	2.817319	-2.289992	2.477795
C	1.787300	-2.593397	3.507615
C	0.399287	-2.096982	3.283559
C	0.049997	-1.349621	2.135050
C	-1.274157	-0.876289	2.009291
C	-2.213226	-1.200021	2.993451
C	-1.860547	-1.949515	4.110187
C	-0.549270	-2.390415	4.258430
C	4.198599	-2.763928	2.740764
O	0.907157	-0.625799	-0.001737
O	2.078067	-3.225640	4.504083
H	3.168166	-1.395511	0.574551
H	-3.238502	-0.838964	2.884870
H	-2.609781	-2.181108	4.870133
H	-0.238018	-2.970551	5.128743
H	4.575282	-2.323021	3.677638
H	4.203442	-3.854866	2.891744
H	4.875956	-2.504248	1.918097
Ru	-0.636736	0.170665	-1.093632
C	-2.809125	-1.787768	-2.788324
C	-1.768538	-0.717576	-2.793972
C	-0.399732	-1.018405	-2.974193

C	0.567375	0.016474	-2.894474
C	0.224478	1.385182	-2.731818
C	-1.161976	1.675305	-2.566772
C	-2.133713	0.650301	-2.578969
C	1.307486	2.445207	-2.694284
C	2.119829	2.381080	-1.400192
C	0.768632	3.849092	-2.939284
H	1.607743	4.552774	-3.038355
H	0.171279	3.903067	-3.862082
H	0.146973	4.190095	-2.098128
H	1.982997	2.187466	-3.528388
H	2.968262	3.079294	-1.453984
H	2.517362	1.372924	-1.213671
H	1.489978	2.667483	-0.544605
H	1.625467	-0.258170	-2.900620
H	-0.076669	-2.055898	-3.071897
H	-1.485266	2.697346	-2.368412
H	-3.173868	0.901799	-2.365953
H	-3.236589	-1.869111	-3.800836
H	-2.380041	-2.761165	-2.517725
H	-3.628218	-1.542439	-2.098921
S	-1.826424	2.719983	0.503609
C	-0.938477	1.236900	0.630902
C	-1.739115	0.005978	0.892614
H	-1.754193	-0.740735	-0.058650
H	-2.820184	0.192150	0.950607
H	-0.062930	1.416376	1.267287
O	-0.954305	3.817381	0.146751
O	-3.099438	2.536002	-0.162706
F	-2.203929	2.976464	2.057756

**TS3 (imaginary frequency: -448.38 cm^{-1})**			
C	1.133551	-1.136534	1.138262

C	2.497317	-1.547923	1.435161
C	2.840377	-2.229485	2.549460
C	1.777029	-2.606151	3.521653
C	0.384591	-2.148069	3.253001
C	0.070226	-1.364388	2.120126
C	-1.249835	-0.893702	1.952201
C	-2.231646	-1.286968	2.870301
C	-1.918256	-2.087884	3.962400
C	-0.604499	-2.505178	4.163528
C	4.226707	-2.652779	2.867401
O	0.948627	-0.638130	0.006703
O	2.047590	-3.262232	4.508622
H	3.240966	-1.284316	0.679248
H	-3.255627	-0.932692	2.731447
H	-2.699933	-2.373904	4.669371
H	-0.326223	-3.115505	5.024386
H	4.541604	-2.215430	3.828517
H	4.270469	-3.745333	2.998544
H	4.929802	-2.348509	2.082432
Ru	-0.657515	0.093960	-0.994330
C	-2.902197	-1.744217	-2.778918
C	-1.846756	-0.690406	-2.734278
C	-0.473966	-1.009815	-2.926193
C	0.512999	0.006038	-2.913944
C	0.195570	1.368984	-2.722229
C	-1.173285	1.663610	-2.455844
C	-2.179458	0.667613	-2.476852
C	1.285197	2.420845	-2.736774
C	2.109747	2.393522	-1.448043
C	0.752816	3.819372	-3.025586
H	1.595946	4.509984	-3.171734
H	0.134660	3.841893	-3.935891
H	0.154933	4.201331	-2.185056
H	1.951419	2.129597	-3.566863

H	2.956013	3.091886	-1.528666
H	2.510676	1.390721	-1.240602
H	1.488133	2.702770	-0.594517
H	1.562652	-0.284858	-3.006656
H	-0.172775	-2.050722	-3.052875
H	-1.470662	2.689215	-2.236483
H	-3.211392	0.945259	-2.258565
H	-3.287844	-1.806511	-3.809250
H	-2.497704	-2.727033	-2.504813
H	-3.742443	-1.494021	-2.118242
S	-1.696020	2.780732	0.538499
C	-0.861606	1.268602	0.710786
C	-1.654546	0.077425	0.905463
H	-1.527202	-1.054953	-0.340599
H	-2.740136	0.195977	0.823619
H	0.053681	1.426086	1.294128
O	-0.796958	3.828671	0.108001
O	-2.999108	2.620700	-0.071961
F	-1.995707	3.102853	2.092507

	**F**		
C	1.155214	-1.065253	1.147297
C	2.532877	-1.400387	1.473799
C	2.882546	-2.087692	2.582328
C	1.814552	-2.546876	3.514439
C	0.407379	-2.155602	3.218350
C	0.083727	-1.355532	2.099012
C	-1.251985	-0.936039	1.907691
C	-2.236841	-1.410445	2.785748
C	-1.914742	-2.235272	3.856600
C	-0.587002	-2.592216	4.086553
C	4.280878	-2.443592	2.928872
O	0.968789	-0.579336	0.009173

O	2.094476	-3.214337	4.491154
H	3.280241	-1.081270	0.743366
H	-3.272183	-1.098413	2.630842
H	-2.700982	-2.585317	4.528952
H	-0.303516	-3.215925	4.935976
H	4.542865	-2.022825	3.912876
H	4.382336	-3.536015	3.024830
H	4.987852	-2.074328	2.175871
Ru	-0.683706	0.036232	-0.980833
C	-3.061570	-1.606928	-2.740432
C	-1.951024	-0.610846	-2.702376
C	-0.594589	-0.998679	-2.960224
C	0.442260	-0.047963	-2.960759
C	0.203191	1.326874	-2.706487
C	-1.141814	1.693718	-2.436540
C	-2.203945	0.756639	-2.444974
C	1.345888	2.320991	-2.714355
C	2.146775	2.263715	-1.412168
C	0.889276	3.742761	-3.021269
H	1.768484	4.388062	-3.161783
H	0.283748	3.789027	-3.939095
H	0.301439	4.163199	-2.191651
H	2.009502	1.989995	-3.531348
H	3.028052	2.918280	-1.484260
H	2.491065	1.243753	-1.188717
H	1.528155	2.612028	-0.571091
H	1.470400	-0.390557	-3.104002
H	-0.358435	-2.051554	-3.123061
H	-1.374387	2.733805	-2.206555
H	-3.214908	1.091296	-2.210212
H	-3.464645	-1.642370	-3.765207
H	-2.706944	-2.611695	-2.476776
H	-3.875983	-1.316068	-2.064391
S	-1.742326	2.760272	0.521122

C	-0.900823	1.248589	0.701244
C	-1.670482	0.053895	0.892341
H	-1.284206	-1.342749	-0.547963
H	-2.755865	0.137442	0.779993
H	0.023951	1.422836	1.264674
O	-0.850801	3.807076	0.071930
O	-3.052859	2.594267	-0.070216
F	-2.016218	3.085065	2.077263

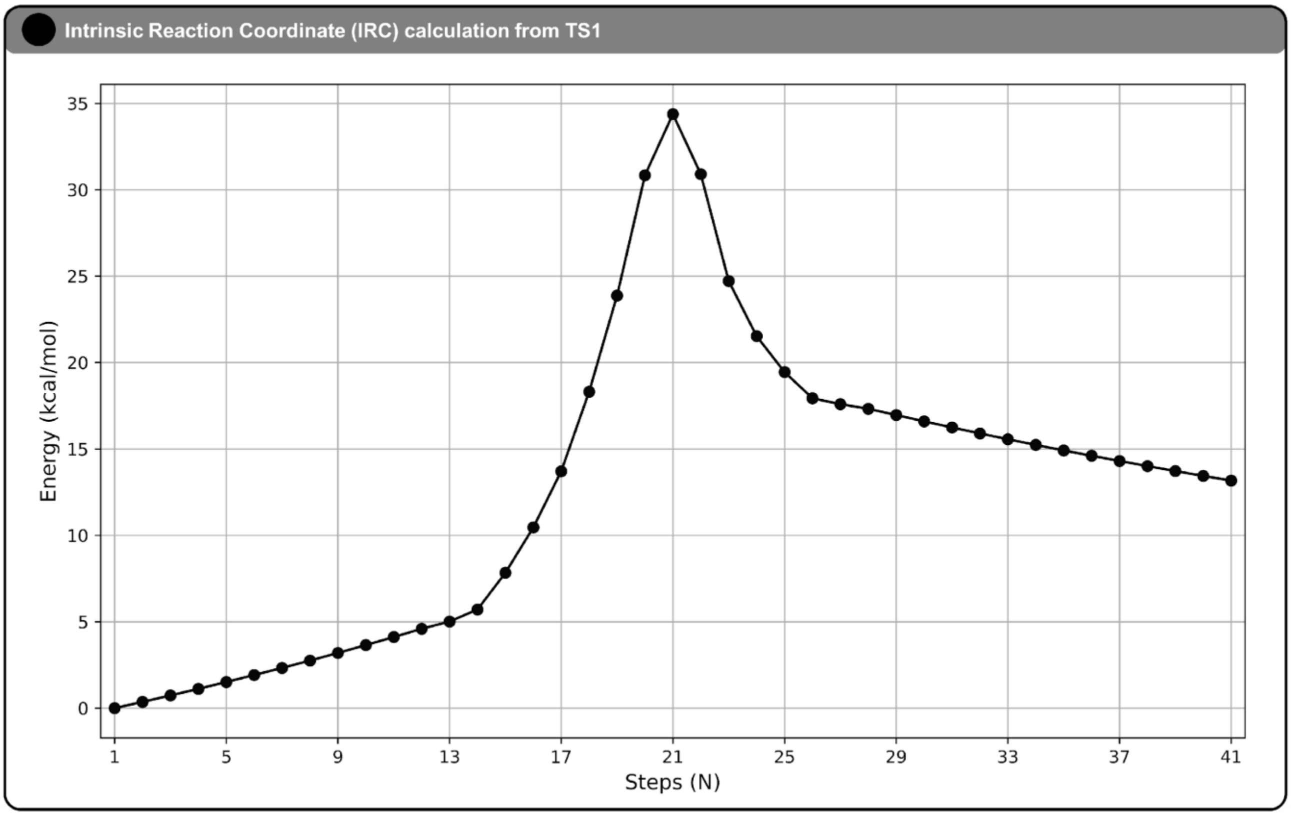

Figure S1. Computed intrinsic reaction coordinate (IRC) pathway from the transition state (**TS1**) of the C–H activation step for the C–H alkenylation of menadione (**1a**).

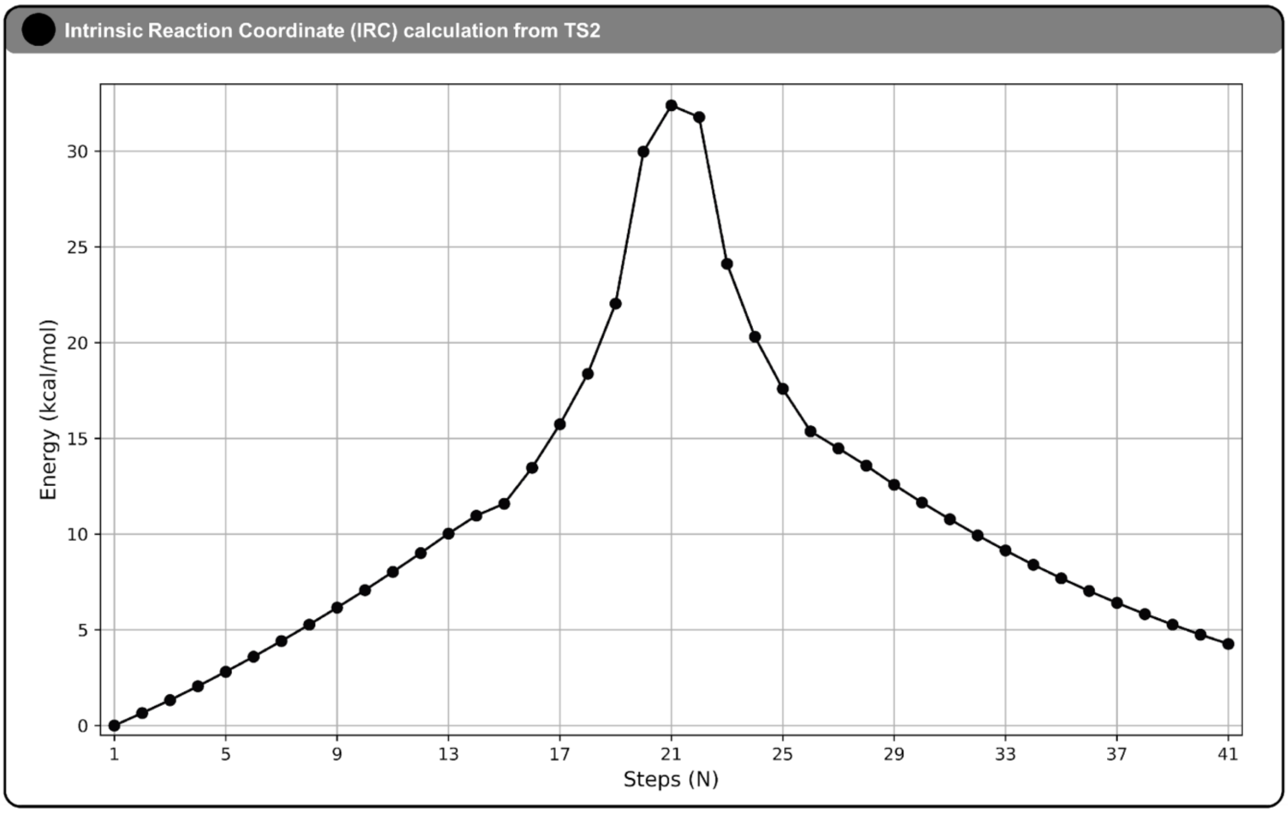

Figure S2. Computed intrinsic reaction coordinate (IRC) pathway from the transition state (**TS2**) of the olefin insertion step for the C–H alkenylation of menadione (**1a**).

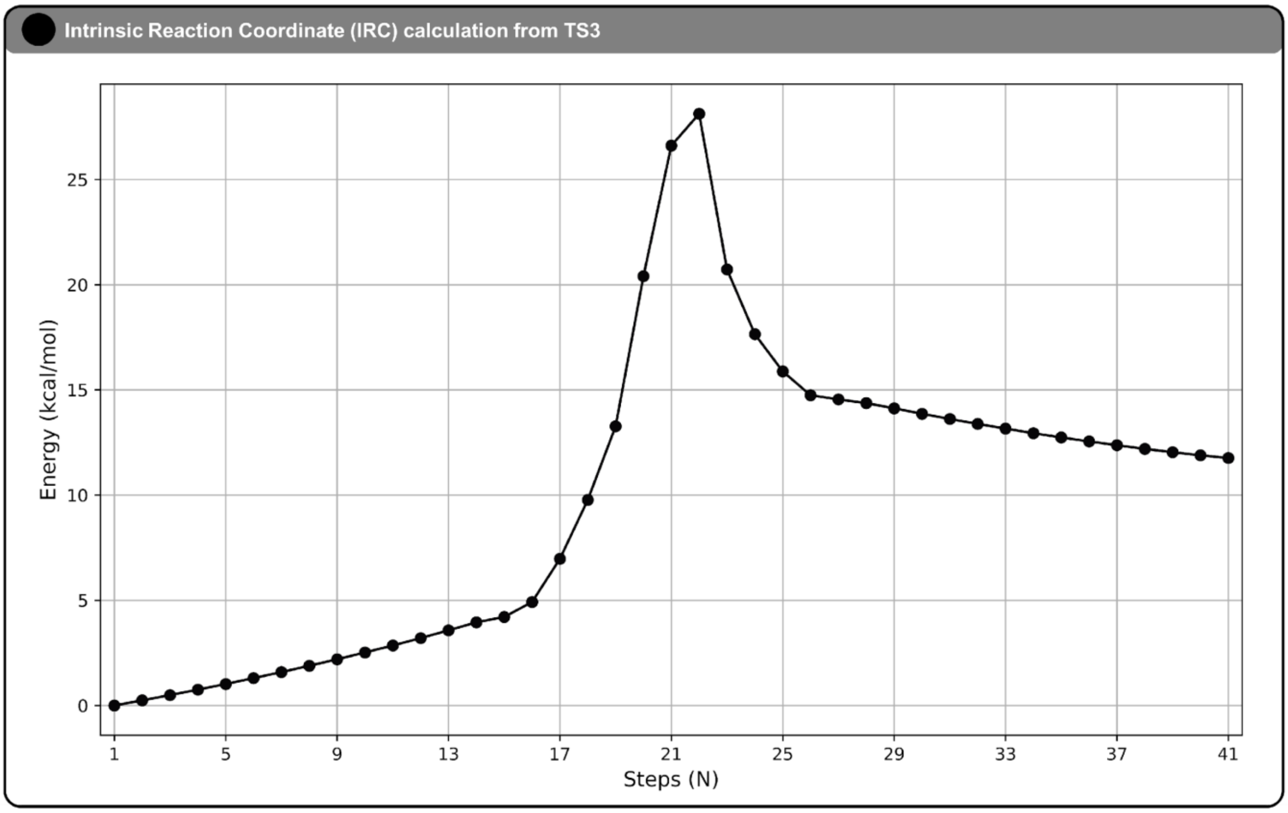

Figure S3. Computed intrinsic reaction coordinate (IRC) pathway from the transition state (**TS3**) of the *β*-elimination step for the C–H alkenylation of menadione (**1a**).

Table S2. Computed free energies (G_{tot}) of all species involved in the C–H alkenylation reaction pathway of menadione (**1a**). G_{tot} was obtained as the sum of the thermal correction to Gibbs free energy (G_{corr}) at the PBE0-D3(BJ)/bs1+CPCM(DCE) level; the electronic energy (E) at the CCSD(T)/def2-TZVPP+CPCM(DCE) level; and the concentration correction (G_{conc}). Herein, bs1 = def2-TZVP for Ru, def2-SVP for other elements; DCE = 1,2-dichloroethane.

Species	G_{corr} (Eh)	E (Eh)	G_{conc} (Eh)	G_{tot} (Eh)
**AcOH**	0.035039	-228.766290	0.003012	-228.728239
**$C_2H_3SO_2F$**	0.025247	-725.574365	0.003012	-725.546107
**A**	0.377204	-1285.047810	0.003012	-1284.667594
**TS1**	0.373144	-1285.014681	0.003012	-1284.638524
**B**	0.378125	-1285.046190	0.003012	-1284.665053
**C**	0.372984	-1781.870860	0.003012	-1781.494864
**TS2**	0.372895	-1781.835983	0.003012	-1781.460076
**D**	0.374349	-1781.862176	0.003012	-1781.484815
**E**	0.372975	-1781.866913	0.003012	-1781.490926
**TS3**	0.370281	-1781.859411	0.003012	-1781.486118
**F**	0.371283	-1781.864632	0.003012	-1781.490337

Table S3. Computed free energies (G_{tot}) of all species involved in the C–H alkenylation reaction pathway of menadione (**1a**). G_{tot} was obtained as the sum of the thermal correction to Gibbs free energy (G_{corr}) at the PBE0-D3(BJ)/bs1+CPCM(DCE) level; the electronic energy (E) at the ωB97X-D3/def2-TZVPP+CPCM(DCE) level; and the concentration correction (G_{conc}). Herein, bs1 = def2-TZVP for Ru, def2-SVP for other elements; DCE = 1,2-dichloroethane.

Species	G_{corr} (Eh)	E (Eh)	G_{conc} (Eh)	G_{tot} (Eh)
**AcOH**	0.035039	-229.133916	0.003012	-229.095865
**$C_2H_3SO_2F$**	0.025247	-726.534766	0.003012	-726.506508
**A**	0.377204	-1287.418396	0.003012	-1287.038180
**TS1**	0.373144	-1287.380112	0.003012	-1287.003955
**B**	0.378125	-1287.410294	0.003012	-1287.029157
**C**	0.372984	-1784.819926	0.003012	-1784.443929
**TS2**	0.372895	-1784.789338	0.003012	-1784.413431

**D**	0.374349	-1784.813555	0.003012	-1784.436194
**E**	0.372975	-1784.825992	0.003012	-1784.450005
**TS3**	0.370281	-1784.814574	0.003012	-1784.441281
**F**	0.371283	-1784.815809	0.003012	-1784.441515

Table S4. Computed free energies (G_{tot}) of all species involved in the C–H alkenylation reaction pathway of menadione (**1a**). G_{tot} was obtained as the sum of the thermal correction to Gibbs free energy (G_{corr}) at the PBE0-D3(BJ)/bs1+CPCM(DCE) level; the electronic energy (E) at the PBE/def2-TZVPP+CPCM(DCE) level; and the concentration correction (G_{conc}). Herein, bs1 = def2-TZVP for Ru, def2-SVP for other elements; DCE = 1,2-dichloroethane.

Species	G_{corr} (Eh)	E (Eh)	G_{conc} (Eh)	G_{tot} (Eh)
**AcOH**	0.035039	-228.942864	0.003012	-228.904813
**$C_2H_3SO_2F$**	0.025247	-726.130618	0.003012	-726.102360
**A**	0.377204	-1286.375881	0.003012	-1285.995665
**TS1**	0.373144	-1286.353567	0.003012	-1285.977411
**B**	0.378125	-1286.375751	0.003012	-1285.994614
**C**	0.372984	-1783.581938	0.003012	-1783.205942
**TS2**	0.372895	-1783.551957	0.003012	-1783.176049
**D**	0.374349	-1783.567670	0.003012	-1783.190309
**E**	0.372975	-1783.578015	0.003012	-1783.202028
**TS3**	0.370281	-1783.573450	0.003012	-1783.200158
**F**	0.371283	-1783.575581	0.003012	-1783.201286

Table S5. Computed free energies (G_{tot}) of all species involved in the C–H alkenylation reaction pathway of menadione (**1a**). G_{tot} was obtained as the sum of the thermal correction to Gibbs free energy (G_{corr}) at the PBE0-D3(BJ)/bs1+CPCM(DCE) level; the electronic energy (E) at the M06/def2-TZVPP+CPCM(DCE) level; and the concentration correction (G_{conc}). Herein, bs1 = def2-TZVP for Ru, def2-SVP for other elements; DCE = 1,2-dichloroethane.

Species	G_{corr} (Eh)	E (Eh)	G_{conc} (Eh)	G_{tot} (Eh)
**AcOH**	0.035039	-229.068806	0.003012	-229.030754
**$C_2H_3SO_2F$**	0.025247	-726.447059	0.003012	-726.418800
**A**	0.377204	-1286.888261	0.003012	-1286.508045
**TS1**	0.373144	-1286.850397	0.003012	-1286.474241

**B**	0.378125	-1286.881908	0.003012	-1286.500771
**C**	0.372984	-1784.273106	0.003012	-1783.897110
**TS2**	0.372895	-1784.245234	0.003012	-1783.869327
**D**	0.374349	-1784.266591	0.003012	-1783.889229
**E**	0.372975	-1784.274843	0.003012	-1783.898856
**TS3**	0.370281	-1784.263313	0.003012	-1783.890020
**F**	0.371283	-1784.264656	0.003012	-1783.890361

Table S6. Computed free energies (G_{tot}) of all species involved in the C–H alkenylation reaction pathway of menadione (**1a**). G_{tot} was obtained as the sum of the thermal correction to Gibbs free energy (G_{corr}) at the PBE0-D3(BJ)/bs1+CPCM(DCE) level; the electronic energy (E) at the M06-L/def2-TZVPP+CPCM(DCE) level; and the concentration correction (G_{conc}). Herein, bs1 = def2-TZVP for Ru, def2-SVP for other elements; DCE = 1,2-dichloroethane.

Species	G_{corr} (Eh)	E (Eh)	G_{conc} (Eh)	G_{tot} (Eh)
**AcOH**	0.035039	-229.162620	0.003012	-229.124569
**$C_2H_3SO_2F$**	0.025247	-726.576373	0.003012	-726.548115
**A**	0.377204	-1287.653940	0.003012	-1287.273724
**TS1**	0.373144	-1287.613110	0.003012	-1287.236953
**B**	0.378125	-1287.638551	0.003012	-1287.257414
**C**	0.372984	-1785.061496	0.003012	-1784.685500
**TS2**	0.372895	-1785.032608	0.003012	-1784.656701
**D**	0.374349	-1785.053260	0.003012	-1784.675899
**E**	0.372975	-1785.063089	0.003012	-1784.687103
**TS3**	0.370281	-1785.051320	0.003012	-1784.678027
**F**	0.371283	-1785.053016	0.003012	-1784.678721

Table S7. Computed free energies (G_{tot}) of all species involved in the C–H alkenylation reaction pathway of menadione (**1a**). G_{tot} was obtained as the sum of the thermal correction to Gibbs free energy (G_{corr}) at the PBE0-D3(BJ)/bs1+CPCM(DCE) level; the electronic energy (E) at the ωB2PLYP/def2-TZVPP+CPCM(DCE) level; and the concentration correction (G_{conc}). Herein, bs1 = def2-TZVP for Ru, def2-SVP for other elements; DCE = 1,2-dichloroethane.

Species	G_{corr} (Eh)	E (Eh)	G_{conc} (Eh)	G_{tot} (Eh)
**AcOH**	0.035039	-228.813171	0.003012	-228.775120

**$C_2H_3SO_2F$**	0.025247	-725.905016	0.003012	-725.876757
**A**	0.377204	-1285.293266	0.003012	-1284.913050
**TS1**	0.373144	-1285.260117	0.003012	-1284.883961
**B**	0.378125	-1285.290506	0.003012	-1284.909368
**C**	0.372984	-1782.396874	0.003012	-1782.020878
**TS2**	0.372895	-1782.364253	0.003012	-1781.988346
**D**	0.374349	-1782.389953	0.003012	-1782.012592
**E**	0.372975	-1782.398917	0.003012	-1782.022931
**TS3**	0.370281	-1782.390437	0.003012	-1782.017145
**F**	0.371283	-1782.392599	0.003012	-1782.018304

Table S8. Computed free energies (G_{tot}) of all species involved in the C–H alkenylation reaction pathway of menadione (**1a**). G_{tot} was obtained as the sum of the thermal correction to Gibbs free energy (G_{corr}) at the PBE0-D3(BJ)/bs1+CPCM(DCE) level; the electronic energy (E) at the B2PLYP/def2-TZVPP+CPCM(DCE) level; and the concentration correction (G_{conc}). Herein, bs1 = def2-TZVP for Ru, def2-SVP for other elements; DCE = 1,2-dichloroethane.

Species	G_{corr} (Eh)	E (Eh)	G_{conc} (Eh)	G_{tot} (Eh)
**AcOH**	0.035039	-229.030366	0.003012	-228.992315
**$C_2H_3SO_2F$**	0.025247	-726.288656	0.003012	-726.260398
**A**	0.377204	-1286.668565	0.003012	-1286.288349
**TS1**	0.373144	-1286.635594	0.003012	-1286.259438
**B**	0.378125	-1286.665338	0.003012	-1286.284201
**C**	0.372984	-1783.936811	0.003012	-1783.560815
**TS2**	0.372895	-1783.901645	0.003012	-1783.525738
**D**	0.374349	-1783.921769	0.003012	-1783.544407
**E**	0.372975	-1783.931385	0.003012	-1783.555398
**TS3**	0.370281	-1783.925556	0.003012	-1783.552263
**F**	0.371283	-1783.928673	0.003012	-1783.554378

Table S9. Computed free energies (G_{tot}) of all species involved in the C–H alkenylation reaction pathway of menadione (**1a**). G_{tot} was obtained as the sum of the thermal correction to Gibbs free energy (G_{corr}) at the PBE0-D3(BJ)/bs1+CPCM(DCE) level; the electronic energy (E) at the BP86/def2-TZVPP+CPCM(DCE) level; and the concentration correction (G_{conc}). Herein, bs1 = def2-TZVP for Ru, def2-SVP for other elements; DCE = 1,2-dichloroethane.

Species	G_{corr} (Eh)	E (Eh)	G_{conc} (Eh)	G_{tot} (Eh)
**AcOH**	0.035039	-229.210625	0.003012	-229.172574
**$C_2H_3SO_2F$**	0.025247	-726.699324	0.003012	-726.671065
**A**	0.377204	-1287.980855	0.003012	-1287.600640
**TS1**	0.373144	-1287.960926	0.003012	-1287.584770
**B**	0.378125	-1287.982982	0.003012	-1287.601845
**C**	0.372984	-1785.493001	0.003012	-1785.117004
**TS2**	0.372895	-1785.462374	0.003012	-1785.086467
**D**	0.374349	-1785.479645	0.003012	-1785.102284
**E**	0.372975	-1785.486499	0.003012	-1785.110513
**TS3**	0.370281	-1785.482394	0.003012	-1785.109101
**F**	0.371283	-1785.485061	0.003012	-1785.110767

Table S10. Computed free energies (G_{tot}) of all species involved in the C–H alkenylation reaction pathway of menadione (**1a**). G_{tot} was obtained as the sum of the thermal correction to Gibbs free energy (G_{corr}) at the PBE0-D3(BJ)/bs1+CPCM(DCE) level; the electronic energy (E) at the B3LYP/def2-TZVPP+CPCM(DCE) level; and the concentration correction (G_{conc}). Herein, bs1 = def2-TZVP for Ru, def2-SVP for other elements; DCE = 1,2-dichloroethane.

Species	G_{corr} (Eh)	E (Eh)	G_{conc} (Eh)	G_{tot} (Eh)
**AcOH**	0.035039	-229.089676	0.003012	-229.051624
**$C_2H_3SO_2F$**	0.025247	-726.445719	0.003012	-726.417461
**A**	0.377204	-1287.111788	0.003012	-1286.731572
**TS1**	0.373144	-1287.079874	0.003012	-1286.703718
**B**	0.378125	-1287.107700	0.003012	-1286.726563
**C**	0.372984	-1784.477155	0.003012	-1784.101158
**TS2**	0.372895	-1784.442840	0.003012	-1784.066932
**D**	0.374349	-1784.463255	0.003012	-1784.085894
**E**	0.372975	-1784.474195	0.003012	-1784.098208
**TS3**	0.370281	-1784.466275	0.003012	-1784.092982
**F**	0.371283	-1784.469390	0.003012	-1784.095095

Table S11. Computed Gibbs free energies (in kcal/mol) obtained from the benchmark of the C–H alkenylation reaction pathway of menadione (**1a**).

Funcional	Reaction Energies							
	**TS1**	**B**	**C**	**TS2**	**D**	**E**	**TS3**	**F**
BP86	9.96	-0.76	-11.22	7.95	-1.98	-7.14	-6.26	-7.30
PBE	11.45	0.66	-7.99	10.77	1.82	-5.53	-4.36	-5.07
B3LYP	17.48	3.14	-2.35	19.12	7.23	-0.50	2.78	1.45
PBE0	15.32	1.06	-6.67	11.78	-1.53	-8.23	-4.73	-4.97
M06	21.21	4.56	-0.64	16.80	4.31	-1.74	3.81	3.60
M06-L	23.07	10.23	7.39	25.46	13.41	6.38	12.08	11.64
ωB97X-D3	21.48	5.66	3.07	22.21	7.92	-0.74	4.73	4.59
B2PLYP	18.14	2.60	-2.75	19.26	7.55	0.65	2.62	1.29
ωB2PLYP	18.25	2.31	-3.88	16.53	1.32	-5.17	-1.54	-2.27
CCSD(T)	18.24	1.59	-5.90	15.93	0.41	-3.43	-0.41	-3.06

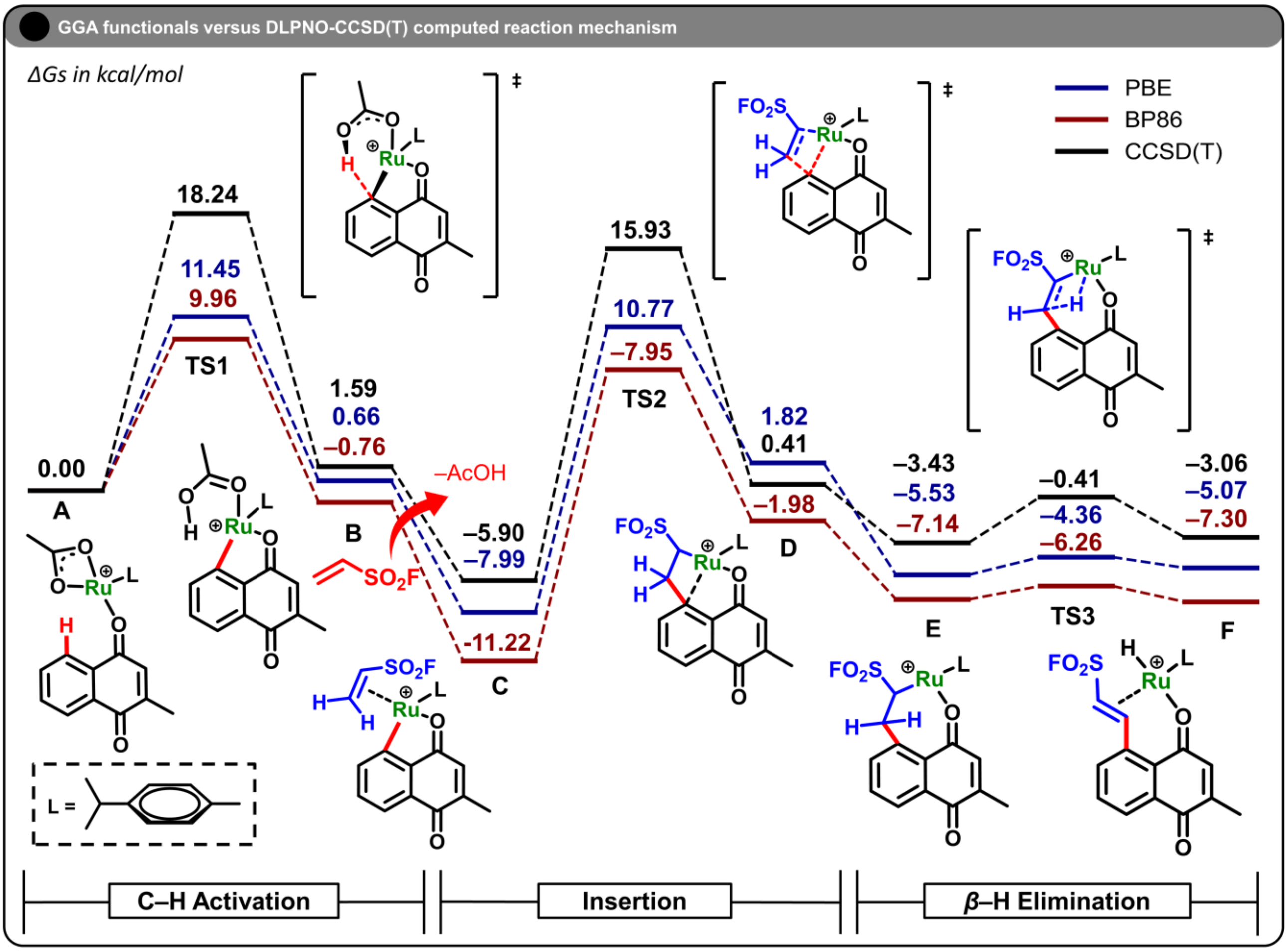

Figure S4. Computed Gibbs free energies (in kcal/mol) for the C–H alkenylation of the menadione (**1a**). Energies were obtained by the PBE (blue line) and BP86 (red line) GGA functionals and compared with the reference values obtained with the CCSD(T) method (black line), from optimized structures at PBE0-D3(BJ)/bs1+CPCM(DCE). Herein, bs1 = def2-TZVP for Ru, def2-SVP for other elements; DCE = 1,2-dichloroethane.

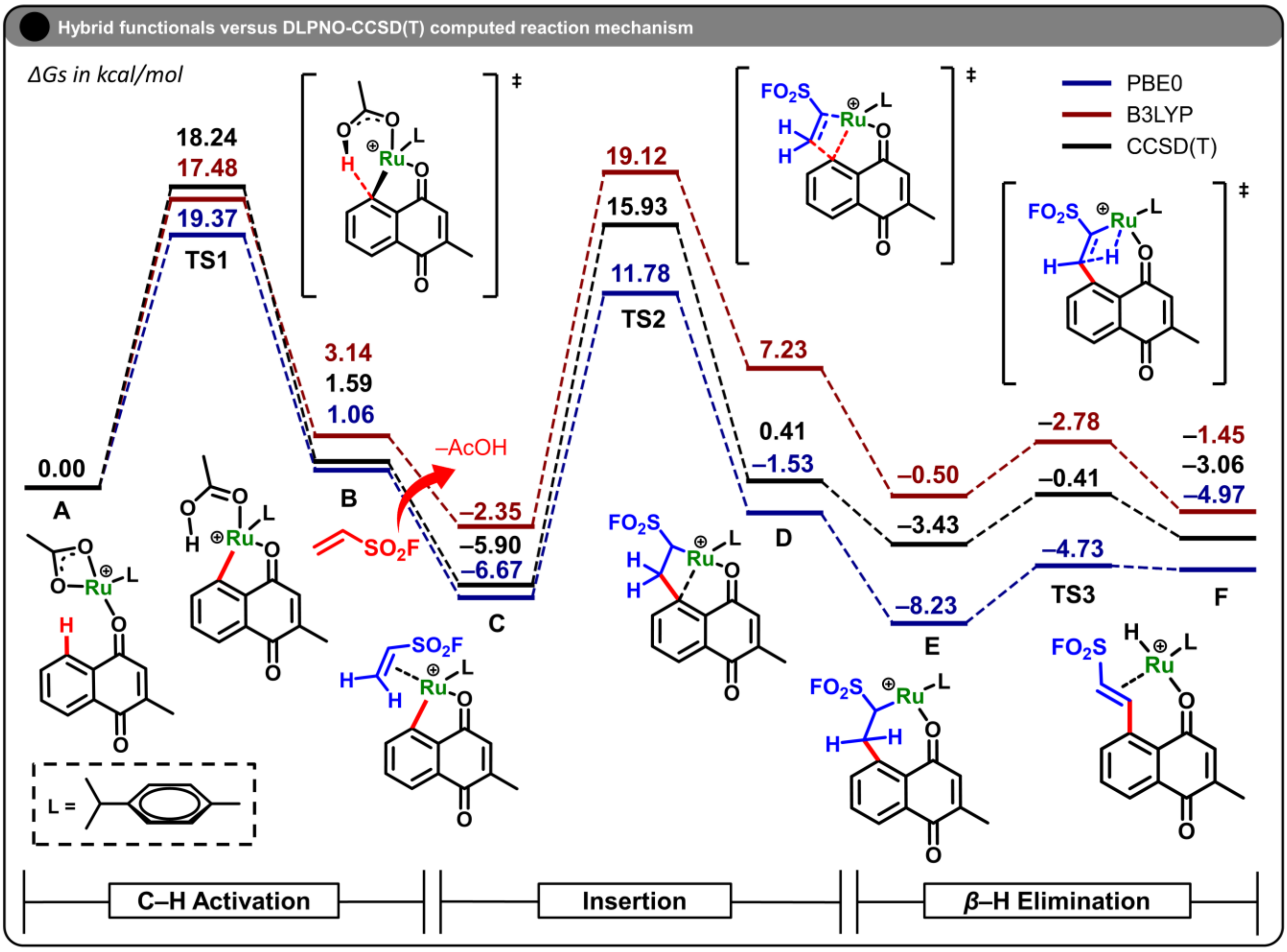

Figure S5. Computed Gibbs free energies (in kcal/mol) for the C–H alkenylation of the menadione (**1a**). Energies were obtained by the PBE0 (blue line) and B3LYP (red line) hybrids functionals and compared with the reference values obtained with the CCSD(T) method (black line), from optimized structures at PBE0-D3(BJ)/bs1+CPCM(DCE). Herein, bs1 = def2-TZVP for Ru, def2-SVP for other elements; DCE = 1,2-dichloroethane.

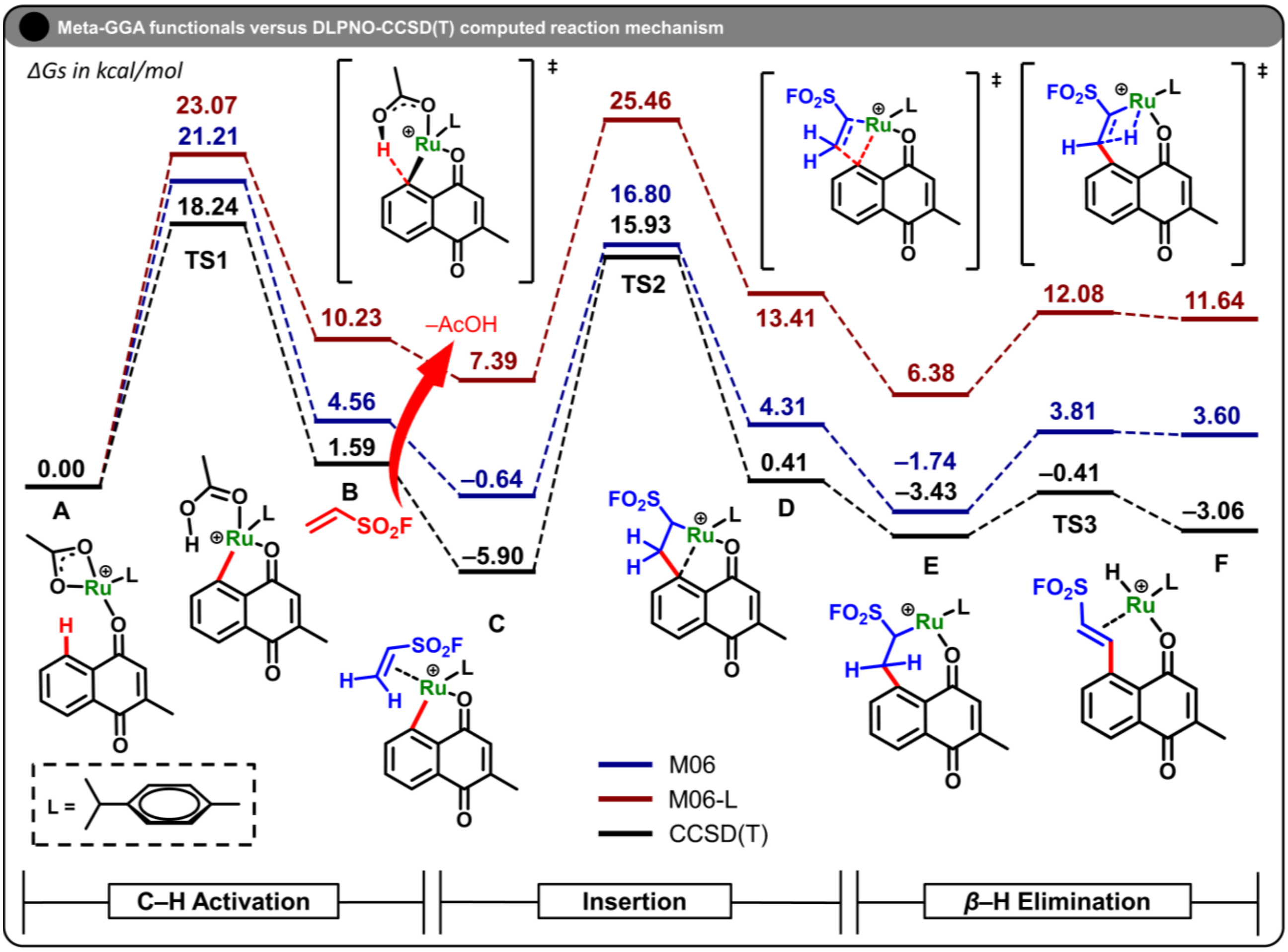

Figure S6. Computed Gibbs free energies (in kcal/mol) for the C–H alkenylation of the menadione (**1a**). Energies were obtained by the M06 (blue line) and M06-L (red line) Minnesota functionals and compared with the reference values obtained with the CCSD(T) method (black line), from optimized structures at PBE0-D3(BJ)/bs1+CPCM(DCE). Herein, bs1 = def2-TZVP for Ru, def2-SVP for other elements; DCE = 1,2-dichloroethane.

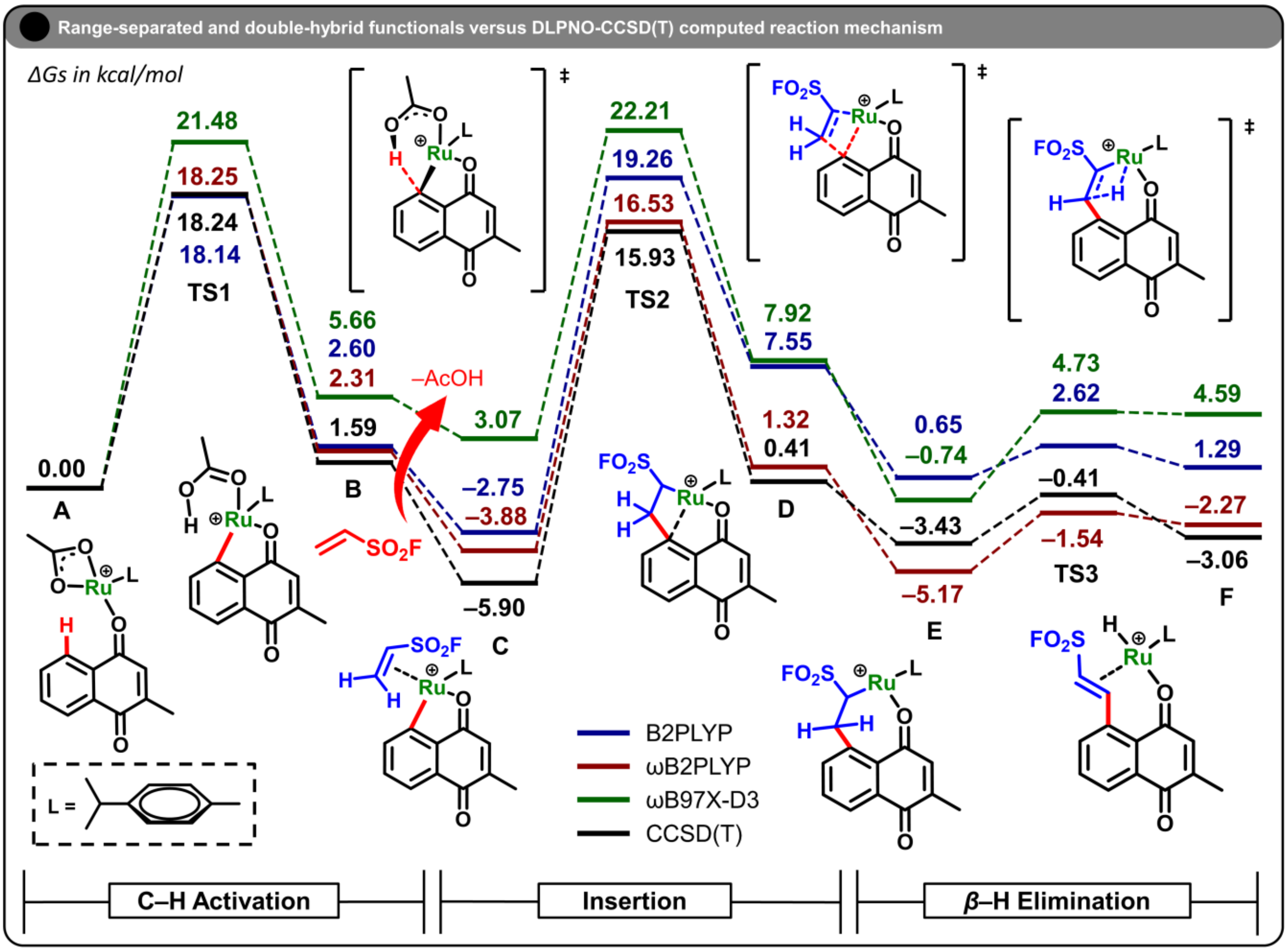

Figure S7. Computed Gibbs free energies in (kcal/mol) for the C–H alkenylation of the menadione (**1a**). Energies were obtained by the B2PLYP (blue line) and ωB2PLYP (red line) double-hybrid functionals and ωB97X-D3 (green line) hybrid functional, and compared with the reference values obtained with the CCSD(T) method (black line), from optimized structures at PBE0-D3(BJ)/bs1+CPCM(DCE). Herein, bs1 = def2-TZVP for Ru, def2-SVP for other elements; DCE = 1,2-dichloroethane.

Table S12. Signed error obtained from the benchmark of the C–H alkenylation reaction pathway of menadione (**1a**) against high-level DLPNO-CCSD(T) reference energies.

Funcional	Signed Error							
	**TS1**	**B**	**C**	**TS2**	**D**	**E**	**TS3**	**F**
BP86	-8.28	-2.35	-5.32	-7.98	-2.39	-3.71	-5.85	-4.24
PBE	-6.79	-0.93	-2.09	-5.16	1.41	-2.1	-3.95	-2.01
B3LYP	-0.76	1.55	3.55	3.19	6.82	2.93	3.19	4.51
PBE0	-2.92	-0.53	-0.77	-4.15	-1.94	-4.8	-4.32	-1.91
M06	2.97	2.97	5.26	0.87	3.90	1.69	4.22	6.66
M06-L	4.83	8.64	13.29	9.53	13.0	9.81	12.49	14.7

ωB97X-D3	3.24	4.07	8.97	6.28	7.51	2.69	5.14	7.65
B2PLYP	-0.10	1.01	3.15	3.33	7.14	4.08	3.03	4.35
ωB2PLYP	0.01	0.72	2.02	0.6	0.91	-1.74	-1.13	0.79

Table S13. Root-mean-square deviation (RMSD) and mean deviation (MAD) obtained from the benchmark of the C–H alkenylation reaction pathway of menadione (**1a**) against high-level DLPNO-CCSD(T) reference energies.

Funcional	BP86	PBE	B3LYP	PBE0	M06	M06-L	ωB97X-D3	B2PLYP	ωB2PLYP
**RMSD**	5.02	3.06	3.31	2.67	3.57	10.79	5.69	3.27	0.99
**MAD**	5.45	3.61	3.73	3.08	3.98	11.20	6.08	3.84	1.16

DFT mechanistic data for compound 1b

Table S14. Computed free energies (G_{tot}) of all species involved in the C–H alkenylation reaction pathway of 2,3-dimethyl-1,4-naphthoquinone (**1b**). G_{tot} was obtained as the sum of the thermal correction to Gibbs free energy (G_{corr}) at the PBE0-D3(BJ)/bs1+CPCM(DCE) level; the electronic energy (E) at the ωB2PLYP/def2-TZVPP+CPCM(DCE) level; and the concentration correction (G_{conc}). Herein, bs1 = def2-TZVP for Ru, def2-SVP for other elements; DCE = 1,2-dichloroethane.

Species	G_{corr} (Eh)	E (Eh)	G_{conc} (Eh)	G_{tot} (Eh)
**AcOH**	0.035039	-228.813171	0.003012	-228.775120
**$C_2H_3SO_2F$**	0.025247	-725.905016	0.003012	-725.876757
**A**	0.403429	-1324.539429	0.003012	-1324.132988
**TS1**	0.398803	-1324.506101	0.003012	-1324.104286
**B**	0.403440	-1324.536061	0.003012	-1324.129609
**C**	0.398792	-1821.638164	0.003012	-1821.236360
**TS2**	0.398682	-1821.610922	0.003012	-1821.209228
**D**	0.400575	-1821.636727	0.003012	-1821.233140
**E**	0.398820	-1821.644476	0.003012	-1821.242645
**TS3**	0.396012	-1821.636278	0.003012	-1821.237254
**F**	0.397108	-1821.638140	0.003012	-1821.238020

Cartesian Coordinates for the Reaction Pathway of Compound 1b

All values are in Ångstrom

**AcOH**			
H	0.535739	-0.008153	-2.653144
O	0.535743	-0.494303	-1.811370
O	0.535743	1.594731	-1.029815
C	0.535751	0.404673	-0.823347
C	0.535744	-0.243430	0.525903
H	1.421457	-0.888024	0.627310
H	0.535741	0.522529	1.308784
H	-0.349969	-0.888024	0.627303

**$C_2H_3SO_2F$**			
C	-1.148281	-1.327974	-1.152267

C	-0.569929	-0.158549	-1.409948
H	0.396424	-0.005222	-1.897992
H	-2.119013	-1.385254	-0.651904
H	-0.652938	-2.259832	-1.437821
S	-1.348911	1.321627	-0.908869
O	-2.601467	1.067464	-0.237289
O	-0.379698	2.251473	-0.379569
F	-1.740750	1.903609	-2.360824

**A**			
C	-1.762700	0.125448	-0.461993
C	-2.702976	1.235019	-0.710075
C	-3.963371	1.176579	-0.199005
C	-4.440739	-0.042766	0.516640
C	-3.555005	-1.232439	0.560727
C	-2.248196	-1.161238	0.049920
C	-1.452280	-2.309419	0.020517
C	-1.946856	-3.509657	0.527238
C	-3.229189	-3.567550	1.072667
C	-4.035252	-2.429659	1.084798
O	-0.565231	0.348178	-0.697330
O	-5.547979	-0.061939	1.021086
H	-0.459797	-2.273871	-0.429858
H	-1.325180	-4.406926	0.492579
H	-3.609469	-4.508085	1.477628
H	-5.049141	-2.456942	1.488316
Ru	1.386430	-0.224536	-0.271625
O	1.648243	0.417710	-2.276409
O	1.151642	-1.629570	-1.840671
C	1.342050	-0.729086	-2.716865
C	1.165560	-0.999925	-4.166476
H	1.729546	-0.275473	-4.766158
H	0.094983	-0.899459	-4.406422

H	1.473268	-2.026527	-4.402559
C	1.700738	-2.972702	1.579314
C	1.988794	-1.523232	1.372956
C	1.126082	-0.515008	1.856207
C	1.381711	0.864272	1.592529
C	2.499159	1.271283	0.834023
C	3.373684	0.246233	0.341773
C	3.124012	-1.115403	0.598849
C	2.763472	2.710232	0.467348
C	3.741648	3.300721	1.485845
C	1.501048	3.550112	0.335393
H	1.757557	4.545746	-0.054356
H	0.781952	3.086261	-0.356172
H	1.002607	3.697397	1.306219
H	3.265669	2.688520	-0.514410
H	4.006126	4.330226	1.202194
H	4.669594	2.712161	1.543881
H	3.288448	3.326370	2.489440
H	0.628859	1.598955	1.880332
H	0.197373	-0.801088	2.353997
H	4.180254	0.520616	-0.341689
H	3.744303	-1.874836	0.118607
H	1.877186	-3.546402	0.658509
H	0.669797	-3.133900	1.917359
H	2.382299	-3.363729	2.351600
C	-2.137195	2.414704	-1.432563
C	-4.957088	2.281794	-0.269469
H	-4.570161	3.171122	-0.776478
H	-5.272055	2.559929	0.748487
H	-5.868587	1.938629	-0.784193
H	-1.390282	2.092939	-2.169850
H	-1.620676	3.088589	-0.728889
H	-2.918280	2.991647	-1.940382

TS1 (imaginary frequency: -659.33 cm^{-1})			
C	-2.119923	0.455528	-0.230941
C	-3.414542	1.127828	-0.274758
C	-4.536018	0.408816	0.019619
C	-4.471853	-1.054817	0.333690
C	-3.154691	-1.737077	0.239194
C	-2.024101	-0.984355	-0.080454
C	-0.749078	-1.567007	-0.234190
C	-0.655061	-2.956245	-0.049574
C	-1.774200	-3.710221	0.302952
C	-3.026970	-3.108709	0.445271
C	-5.879384	1.045514	0.061302
O	-1.052863	1.104264	-0.293482
O	-5.475438	-1.669935	0.637491
C	-3.453715	2.589549	-0.570976
H	-0.025474	-1.263697	-1.235917
H	0.301529	-3.458522	-0.213409
H	-1.672651	-4.787872	0.449973
H	-3.910820	-3.694810	0.703808
H	-5.890661	1.884498	0.775670
H	-6.134258	1.469726	-0.923813
H	-6.646425	0.317325	0.347929
Ru	0.710585	0.052772	0.115446
O	1.174016	0.597194	-1.815045
O	0.363123	-1.291025	-2.633909
C	0.943303	-0.198742	-2.785208
C	1.439029	0.229579	-4.132812
H	1.261152	1.303141	-4.278022
H	0.961506	-0.355741	-4.926710
H	2.527036	0.063034	-4.167850
C	-0.168271	-1.416713	3.042042
C	0.801383	-0.625727	2.227778
C	0.722028	0.779155	2.154101
C	1.649718	1.549109	1.384570

C	2.676359	0.920003	0.649954
C	2.725645	-0.510497	0.660156
C	1.808351	-1.256658	1.427727
C	3.639491	1.674595	-0.228721
C	5.009411	1.687369	0.456245
C	3.178014	3.075741	-0.597883
H	3.882925	3.517561	-1.316964
H	2.182072	3.059660	-1.063913
H	3.146023	3.739494	0.280358
H	3.721826	1.084085	-1.156210
H	5.748252	2.175120	-0.196612
H	5.366330	0.669683	0.674431
H	4.967231	2.246990	1.403890
H	1.506388	2.627019	1.312795
H	-0.111857	1.289925	2.641140
H	3.437210	-1.031434	0.016970
H	1.826782	-2.346053	1.358771
H	-0.312167	-2.423706	2.630549
H	-1.140940	-0.909117	3.098306
H	0.222286	-1.521911	4.066566
H	-2.453548	2.977854	-0.795369
H	-3.864174	3.149318	0.284950
H	-4.115137	2.789501	-1.428286

**B**			
C	-2.159880	0.546530	0.084555
C	-3.424262	1.242898	-0.142518
C	-4.544337	0.505145	-0.390347
C	-4.508497	-0.993376	-0.436568
C	-3.206083	-1.670525	-0.215329
C	-2.067737	-0.890359	0.020638
C	-0.780895	-1.424501	0.230106
C	-0.693560	-2.828299	0.201319

C	-1.821433	-3.621855	-0.018308
C	-3.082725	-3.054357	-0.232750
C	-5.865064	1.151625	-0.621075
O	-1.108970	1.173892	0.355457
O	-5.524016	-1.630245	-0.650066
C	-3.443488	2.734286	-0.084145
H	1.259571	-1.803086	-1.272635
H	0.267619	-3.329164	0.349065
H	-1.714956	-4.709694	-0.026482
H	-3.962635	-3.675204	-0.410538
H	-6.159104	1.756130	0.252573
H	-5.814626	1.843536	-1.477321
H	-6.638003	0.398427	-0.811486
Ru	0.627218	0.021006	0.501011
O	0.565274	0.342321	-1.585284
O	1.270809	-1.662125	-2.242314
C	0.859799	-0.447970	-2.486277
C	0.757882	-0.085376	-3.920433
H	0.533881	0.980643	-4.027202
H	-0.044876	-0.685951	-4.375606
H	1.694173	-0.342925	-4.435049
C	0.283703	-1.393903	3.490813
C	1.111033	-0.612835	2.522713
C	1.037227	0.801361	2.450747
C	1.866413	1.575817	1.576380
C	2.709649	0.937641	0.661114
C	2.727967	-0.501486	0.660897
C	1.983180	-1.256772	1.590461
C	3.531963	1.674499	-0.366147
C	4.974727	1.780169	0.135363
C	2.964142	3.032950	-0.750845
H	3.552076	3.460918	-1.575885
H	1.917721	2.951602	-1.081796
H	3.008806	3.745861	0.087162

H	3.536246	1.037813	-1.267767
H	5.610190	2.244218	-0.633685
H	5.394457	0.791325	0.374136
H	5.024761	2.401668	1.043342
H	1.750832	2.659629	1.562278
H	0.298227	1.316407	3.068620
H	3.329743	-1.022873	-0.087620
H	2.021595	-2.346963	1.547756
H	0.011966	-2.375382	3.079805
H	-0.633858	-0.849822	3.752433
H	0.859199	-1.559344	4.415509
H	-2.449831	3.134839	0.148422
H	-4.154911	3.085081	0.680187
H	-3.777656	3.156127	-1.045609

**C**			
C	1.607972	-0.669931	0.791924
C	3.044937	-0.763204	1.038424
C	3.509474	-1.875885	1.679102
C	2.584368	-2.963140	2.153095
C	1.127402	-2.808628	1.921188
C	0.685296	-1.667629	1.241547
C	-0.665537	-1.401122	0.964853
C	-1.591999	-2.350333	1.425077
C	-1.164599	-3.497358	2.094051
C	0.192971	-3.740228	2.347560
C	4.945164	-2.136436	1.966019
O	1.113704	0.310308	0.179202
O	3.037343	-3.940412	2.718162
C	3.889862	0.366753	0.547357
H	-2.662921	-2.201570	1.272979
H	-1.909717	-4.220931	2.433954
H	0.519609	-4.637413	2.876338

H	5.086433	-2.338522	3.038813
H	5.264814	-3.051441	1.441411
H	5.597133	-1.312058	1.660873
Ru	-0.941148	0.306051	-0.087653
C	-4.183664	-0.703233	-0.555729
C	-2.903160	-0.157064	-1.094642
C	-1.913505	-0.986075	-1.676012
C	-0.725242	-0.431108	-2.182283
C	-0.548458	0.990513	-2.325961
C	-1.545171	1.815694	-1.786476
C	-2.651338	1.246577	-1.108564
C	0.625199	1.541711	-3.102339
C	1.972708	0.925513	-2.732582
C	0.701953	3.062561	-3.085732
H	1.559624	3.391693	-3.689247
H	-0.198197	3.531032	-3.509595
H	0.842551	3.441179	-2.061146
H	0.392592	1.223712	-4.137833
H	2.722704	1.229488	-3.476350
H	1.953955	-0.173067	-2.710741
H	2.300729	1.280584	-1.747505
H	0.055741	-1.109347	-2.529316
H	-2.025535	-2.071745	-1.641001
H	-1.458706	2.900188	-1.828937
H	-3.368438	1.916348	-0.631001
H	-4.967052	-0.575886	-1.319696
H	-4.105122	-1.774100	-0.331392
H	-4.506295	-0.162190	0.343575
S	-0.823478	3.279652	1.168148
C	-0.801425	1.580227	1.584801
C	-1.995689	0.847959	1.725326
H	-2.952162	1.329169	1.507726
H	-2.028972	0.043731	2.462561
H	0.088807	1.355457	2.181293

O	0.414647	3.683609	0.540579
O	-2.101731	3.698703	0.633836
F	-0.758291	3.898310	2.656269
H	3.337769	1.313810	0.615555
H	4.149833	0.219537	-0.513839
H	4.823866	0.457945	1.113121

**TS2 (imaginary frequency: -260.43 cm^{-1})**			
C	1.684578	-0.959758	0.895548
C	3.022328	-1.514884	1.094280
C	3.135627	-2.686938	1.784707
C	1.931210	-3.371890	2.365353
C	0.603702	-2.717042	2.239125
C	0.523801	-1.513848	1.537072
C	-0.681196	-0.782444	1.412974
C	-1.825296	-1.344179	2.007376
C	-1.756796	-2.566504	2.668218
C	-0.546397	-3.257866	2.802251
C	4.412050	-3.413316	2.020212
O	1.505647	0.022185	0.133109
O	2.054597	-4.435570	2.941297
C	4.136573	-0.756978	0.453213
H	-2.772076	-0.799667	1.976236
H	-2.665662	-2.981562	3.110099
H	-0.489387	-4.202696	3.345361
H	4.569638	-3.549760	3.101653
H	4.348033	-4.426771	1.593895
H	5.280519	-2.901807	1.594377
Ru	-0.491786	0.450714	-0.322823
C	-2.943009	-1.994436	-0.820700
C	-2.095364	-0.867494	-1.297641
C	-0.835567	-1.111469	-1.887375
C	-0.071407	-0.039734	-2.414977

C	-0.532527	1.296651	-2.417760
C	-1.757500	1.540087	-1.730371
C	-2.518440	0.487675	-1.167130
C	0.288239	2.374856	-3.098124
C	1.543253	2.730719	-2.301746
C	-0.532172	3.610512	-3.447069
H	0.070394	4.285495	-4.072223
H	-1.440263	3.349136	-4.011539
H	-0.825679	4.166023	-2.545321
H	0.616694	1.912211	-4.045025
H	2.172390	3.422144	-2.881680
H	2.142897	1.839112	-2.066866
H	1.268167	3.224038	-1.358791
H	0.926645	-0.251292	-2.807076
H	-0.426924	-2.123373	-1.903098
H	-2.128382	2.559488	-1.626874
H	-3.442493	0.719893	-0.635877
H	-3.503426	-2.376777	-1.690010
H	-2.337203	-2.818563	-0.423188
H	-3.672886	-1.671291	-0.069154
S	-1.176544	3.441121	0.810933
C	-0.141133	2.049397	0.954809
C	-0.615553	1.021718	1.872461
H	-1.636421	1.157064	2.241253
H	0.094002	0.791242	2.672819
H	0.902045	2.377776	1.020141
O	-0.684048	4.387031	-0.166102
O	-2.580830	3.087346	0.858902
F	-0.880010	4.136075	2.241032
H	4.129190	0.288694	0.797237
H	3.993115	-0.727419	-0.638588
H	5.118685	-1.190334	0.665146

D			
C	1.565258	-0.320083	1.119933
C	2.690967	-1.218701	0.820051
C	2.575852	-2.554461	1.059304
C	1.317166	-3.123054	1.614903
C	0.229215	-2.200091	2.017851
C	0.371476	-0.797258	1.827497
C	-0.647743	0.089034	2.289599
C	-1.817979	-0.490119	2.847902
C	-1.949548	-1.852012	3.001965
C	-0.909002	-2.710710	2.604537
C	3.624353	-3.564246	0.753092
O	1.605977	0.889189	0.745154
O	1.190200	-4.328721	1.738319
C	3.890587	-0.558377	0.222759
H	-2.603735	0.181980	3.195926
H	-2.850497	-2.265303	3.459762
H	-0.982141	-3.790213	2.750279
H	3.809225	-4.194891	1.635684
H	3.260836	-4.244010	-0.034657
H	4.566054	-3.116465	0.421098
Ru	-0.282179	0.350981	-0.126111
C	-2.215577	-2.407295	-0.503700
C	-1.452574	-1.274970	-1.099016
C	-0.106023	-1.428393	-1.527084
C	0.609132	-0.323550	-2.017611
C	-0.009448	0.952548	-2.232167
C	-1.380043	1.060515	-1.896405
C	-2.078565	-0.008454	-1.285152
C	0.776428	2.095612	-2.838639
C	2.049024	2.415325	-2.057222
C	-0.058434	3.349902	-3.055553
H	0.556071	4.118416	-3.545469
H	-0.931386	3.161219	-3.697816

H	-0.416419	3.763471	-2.099798
H	1.078690	1.710557	-3.830578
H	2.641487	3.159281	-2.608678
H	2.682576	1.532041	-1.898984
H	1.799304	2.833159	-1.071117
H	1.676255	-0.437434	-2.219907
H	0.407532	-2.379425	-1.371945
H	-1.884651	2.020355	-1.993526
H	-3.102699	0.141664	-0.939968
H	-2.839875	-2.850615	-1.296603
H	-1.553129	-3.189774	-0.115260
H	-2.888936	-2.060470	0.291393
S	-2.338465	2.712693	0.793459
C	-0.710505	2.105085	0.974425
C	-0.468646	1.589730	2.382711
H	-1.150785	2.005187	3.137363
H	0.551952	1.839916	2.696404
H	-0.057068	2.923907	0.644224
O	-2.525360	3.458117	-0.436491
O	-3.342480	1.756826	1.220250
F	-2.344703	3.849687	1.953455
H	4.022272	0.451101	0.633465
H	3.766811	-0.445976	-0.867315
H	4.803571	-1.138027	0.397550

**E**			
C	1.079418	-1.155949	1.175306
C	2.456206	-1.643950	1.394194
C	2.784594	-2.311514	2.532957
C	1.749155	-2.568748	3.566326
C	0.368696	-2.058594	3.350643
C	0.033256	-1.328782	2.192808
C	-1.286163	-0.844695	2.052629

C	-2.227545	-1.136322	3.044251
C	-1.884219	-1.865139	4.177447
C	-0.579807	-2.321473	4.333422
C	4.130478	-2.854999	2.861942
O	0.880995	-0.626344	0.065858
O	2.030336	-3.186684	4.576635
C	3.402024	-1.355683	0.274841
H	-3.248914	-0.767126	2.927258
H	-2.636534	-2.070394	4.941850
H	-0.274634	-2.890016	5.213215
H	4.454247	-2.476572	3.843625
H	4.074278	-3.950625	2.962963
H	4.889902	-2.606892	2.114621
Ru	-0.636367	0.173201	-1.058390
C	-2.729560	-1.845945	-2.782616
C	-1.712292	-0.754023	-2.773327
C	-0.334430	-1.024936	-2.922739
C	0.607932	0.032343	-2.830603
C	0.231796	1.394093	-2.689962
C	-1.163843	1.654440	-2.553058
C	-2.111789	0.607410	-2.574235
C	1.289517	2.479620	-2.649530
C	2.066453	2.468316	-1.333145
C	0.727026	3.863839	-2.948044
H	1.553675	4.581811	-3.049854
H	0.150080	3.877581	-3.885222
H	0.079406	4.216260	-2.131612
H	1.994384	2.216587	-3.457250
H	2.894041	3.191667	-1.378249
H	2.490243	1.477118	-1.117915
H	1.404502	2.749592	-0.500725
H	1.671583	-0.218967	-2.813029
H	0.014036	-2.055435	-3.004328
H	-1.512470	2.671286	-2.371170

H	-3.161369	0.837173	-2.384505
H	-3.142890	-1.934359	-3.800345
H	-2.283764	-2.810468	-2.507770
H	-3.562023	-1.618906	-2.103119
S	-1.906505	2.712967	0.481058
C	-0.976252	1.257347	0.647826
C	-1.754259	0.013764	0.918709
H	-1.753012	-0.742017	-0.025295
H	-2.838574	0.182526	0.966045
H	-0.111808	1.466859	1.289641
O	-1.074817	3.824823	0.074077
O	-3.182460	2.468316	-0.161291
F	-2.273576	3.015443	2.028477
H	3.461806	-0.271716	0.094048
H	3.034322	-1.807515	-0.659193
H	4.408687	-1.733733	0.474718

**TS3 (imaginary frequency: -435.87 cm^{-1})**			
C	1.124836	-1.137770	1.199115
C	2.509177	-1.562723	1.477372
C	2.792198	-2.293749	2.589669
C	1.704446	-2.668455	3.531764
C	0.328186	-2.163920	3.282231
C	0.048257	-1.352235	2.166202
C	-1.257392	-0.845097	1.991515
C	-2.254874	-1.222494	2.898919
C	-1.972552	-2.047515	3.981423
C	-0.674235	-2.509165	4.181791
C	4.137355	-2.804718	2.969642
O	0.938155	-0.626709	0.076206
O	1.943580	-3.368356	4.498568
C	3.506665	-1.158782	0.441801
H	-3.268465	-0.840838	2.757071

H	-2.767710	-2.321780	4.677843
H	-0.419088	-3.145512	5.030598
H	4.397608	-2.451329	3.979519
H	4.114213	-3.904168	3.029679
H	4.924775	-2.503509	2.272446
Ru	-0.648835	0.103763	-0.958628
C	-2.821205	-1.834381	-2.744117
C	-1.795445	-0.751830	-2.699329
C	-0.413111	-1.035608	-2.862097
C	0.546447	0.008900	-2.864153
C	0.188685	1.364743	-2.711572
C	-1.189812	1.625202	-2.452935
C	-2.167558	0.601095	-2.463736
C	1.246115	2.447940	-2.758743
C	2.082206	2.474727	-1.478163
C	0.670543	3.822665	-3.076275
H	1.491491	4.533358	-3.250101
H	0.042436	3.804132	-3.979868
H	0.070700	4.208316	-2.238855
H	1.913843	2.156739	-3.587733
H	2.907952	3.194031	-1.583222
H	2.513361	1.488449	-1.254114
H	1.460651	2.785405	-0.625216
H	1.604084	-0.255304	-2.943365
H	-0.080949	-2.069946	-2.961433
H	-1.519064	2.645483	-2.255944
H	-3.208915	0.853523	-2.260517
H	-3.196565	-1.913572	-3.777124
H	-2.392410	-2.804031	-2.460754
H	-3.673371	-1.602890	-2.092119
S	-1.713992	2.811844	0.527041
C	-0.853341	1.315419	0.723373
C	-1.645338	0.126692	0.938969
H	-1.533842	-1.021312	-0.289811

H	-2.729931	0.250561	0.853711
H	0.062204	1.491034	1.300178
O	-0.841364	3.877005	0.083971
O	-3.012860	2.614686	-0.082775
F	-2.025942	3.149490	2.075676
H	3.493043	-0.066810	0.305323
H	3.242540	-1.598001	-0.532648
H	4.523535	-1.467512	0.701238

**F**			
C	1.146782	-1.115424	1.197163
C	2.536654	-1.499632	1.504263
C	2.810130	-2.247363	2.607750
C	1.707255	-2.679343	3.507787
C	0.326511	-2.198676	3.237879
C	0.057936	-1.359259	2.139235
C	-1.250054	-0.861504	1.947416
C	-2.262789	-1.289909	2.816760
C	-1.993942	-2.149631	3.874734
C	-0.691951	-2.593000	4.097519
C	4.158992	-2.727151	3.014320
O	0.967147	-0.615029	0.066506
O	1.939359	-3.403690	4.458239
C	3.548535	-1.043527	0.505079
H	-3.278402	-0.918187	2.663607
H	-2.802165	-2.463513	4.538710
H	-0.447995	-3.252077	4.932116
H	4.377725	-2.394163	4.040821
H	4.169351	-3.828059	3.043777
H	4.956587	-2.382095	2.349739
Ru	-0.650973	0.051295	-0.949523
C	-2.893902	-1.792228	-2.750352
C	-1.848585	-0.729306	-2.692985

C	-0.466232	-1.038298	-2.885689
C	0.511705	-0.019816	-2.915708
C	0.182710	1.338967	-2.721991
C	-1.184476	1.621216	-2.435441
C	-2.189286	0.623242	-2.447902
C	1.256566	2.404366	-2.785398
C	2.109496	2.416916	-1.515651
C	0.700738	3.788513	-3.097373
H	1.531849	4.484400	-3.282226
H	0.061199	3.779895	-3.993069
H	0.118542	4.185839	-2.253073
H	1.908428	2.100395	-3.622317
H	2.947657	3.119714	-1.632870
H	2.523285	1.422551	-1.295174
H	1.505685	2.742316	-0.655401
H	1.559997	-0.302409	-3.041741
H	-0.160641	-2.078745	-3.005773
H	-1.487977	2.646744	-2.225006
H	-3.222767	0.899324	-2.235707
H	-3.267004	-1.853078	-3.785406
H	-2.483981	-2.772773	-2.476715
H	-3.742669	-1.551434	-2.097557
S	-1.657313	2.822884	0.515630
C	-0.818284	1.310705	0.710312
C	-1.622275	0.142420	0.927866
H	-1.351318	-1.263138	-0.480678
H	-2.704150	0.266113	0.818783
H	0.112129	1.479264	1.265031
O	-0.772779	3.876217	0.068288
O	-2.964009	2.647485	-0.083247
F	-1.947693	3.155795	2.068184
H	3.506821	0.050656	0.393917
H	3.322529	-1.466755	-0.485786
H	4.566883	-1.329359	0.784287

DFT mechanistic data for compound 1c

Table S15. Computed free energies (G_{tot}) of all species involved in the C–H alkenylation reaction pathway of 2-methoxy-1,4-naphthoquinone (**1c**). G_{tot} was obtained as the sum of the thermal correction to Gibbs free energy (G_{corr}) at the PBE0-D3(BJ)/bs1+CPCM(DCE) level; the electronic energy (E) at the ωB2PLYP/def2-TZVPP+CPCM(DCE) level; and the concentration correction (G_{conc}). Herein, bs1 = def2-TZVP for Ru, def2-SVP for other elements; DCE = 1,2-dichloroethane.

Species	G_{corr} (Eh)	E (Eh)	G_{conc} (Eh)	G_{tot} (Eh)
**AcOH**	0.035039	-228.813171	0.003012	-228.775120
**$C_2H_3SO_2F$**	0.025247	-725.905016	0.003012	-725.876757
**A**	0.380893	-1360.435847	0.003012	-1360.051943
**TS1**	0.378011	-1360.403471	0.003012	-1360.022448
**B**	0.382783	-1360.433572	0.003012	-1360.047777
**C**	0.377339	-1857.536972	0.003012	-1857.156621
**TS2**	0.378028	-1857.507669	0.003012	-1857.126629
**D**	0.379167	-1857.532692	0.003012	-1857.150513
**E**	0.377735	-1857.541042	0.003012	-1857.160295
**TS3**	0.375052	-1857.532530	0.003012	-1857.154466
**F**	0.376038	-1857.534829	0.003012	-1857.155779

Cartesian Coordinates for the Reaction Pathway of Compound 1c

All values are in Ångstrom

AcOH

H	0.535739	-0.008153	-2.653144
O	0.535743	-0.494303	-1.811370
O	0.535743	1.594731	-1.029815
C	0.535751	0.404673	-0.823347
C	0.535744	-0.243430	0.525903
H	1.421457	-0.888024	0.627310
H	0.535741	0.522529	1.308784
H	-0.349969	-0.888024	0.627303

$C_2H_3SO_2F$

C	-1.148281	-1.327974	-1.152267

C	-0.569929	-0.158549	-1.409948
H	0.396424	-0.005222	-1.897992
H	-2.119013	-1.385254	-0.651904
H	-0.652938	-2.259832	-1.437821
S	-1.348911	1.321627	-0.908869
O	-2.601467	1.067464	-0.237289
O	-0.379698	2.251473	-0.379569
F	-1.740750	1.903609	-2.360824

**A**			
C	-1.703675	0.148967	-0.508229
C	-2.577228	1.286462	-0.611705
C	-3.838375	1.259977	-0.094620
C	-4.386715	0.024802	0.572944
C	-3.521894	-1.177318	0.557538
C	-2.228413	-1.133127	0.002102
C	-1.479025	-2.307011	-0.085941
C	-1.992921	-3.503173	0.414317
C	-3.255416	-3.535086	1.004650
C	-4.023141	-2.373277	1.067984
O	-4.690583	2.252203	-0.079388
O	-0.515171	0.295618	-0.857860
O	-5.489743	0.042197	1.070479
H	-0.502612	-2.293130	-0.572171
H	-1.400449	-4.417510	0.337838
H	-3.651537	-4.471895	1.402687
H	-5.025057	-2.379885	1.501501
Ru	1.413128	-0.288930	-0.327336
O	1.777249	0.246651	-2.347338
O	1.236150	-1.770548	-1.831795
C	1.475511	-0.917215	-2.742581
C	1.357959	-1.260576	-4.183023
H	1.929944	-0.554842	-4.797086

H	0.294956	-1.196524	-4.465030
H	1.693900	-2.290897	-4.357012
C	1.714550	-2.989158	1.569562
C	1.976408	-1.534909	1.361296
C	1.061012	-0.546790	1.789962
C	1.285796	0.834073	1.511854
C	2.420330	1.266128	0.793507
C	3.350005	0.261228	0.364926
C	3.136985	-1.104344	0.639582
C	2.642196	2.707325	0.406263
C	3.513565	3.374976	1.472320
C	1.350878	3.477065	0.161426
H	1.584659	4.474879	-0.237211
H	0.705650	2.959155	-0.564528
H	0.778568	3.623040	1.090782
H	3.212516	2.686557	-0.537559
H	3.748147	4.408517	1.176980
H	4.462144	2.835806	1.614964
H	2.987507	3.405117	2.439675
H	0.496047	1.547956	1.749980
H	0.120486	-0.853345	2.252064
H	4.176363	0.549482	-0.288661
H	3.804828	-1.850494	0.204874
H	1.977907	-3.570200	0.674714
H	0.664482	-3.174549	1.827779
H	2.343184	-3.349054	2.399742
C	-4.324101	3.491750	-0.667394
H	-5.188219	4.153775	-0.548540
H	-4.096574	3.357760	-1.735958
H	-3.449970	3.917772	-0.151281
H	-2.147099	2.185394	-1.053549

TS1 (imaginary frequency: -681.22 cm^{-1})

C	-2.115273	0.502217	-0.209886
C	-3.383442	1.162825	-0.243129
C	-4.520862	0.441150	-0.012314
C	-4.487734	-1.049039	0.261791
C	-3.165061	-1.712476	0.185460
C	-2.027445	-0.949978	-0.096593
C	-0.754111	-1.534888	-0.239811
C	-0.666012	-2.928007	-0.074534
C	-1.789931	-3.689406	0.243214
C	-3.043811	-3.089287	0.367685
O	-5.738223	0.913588	0.030211
O	-1.039250	1.147054	-0.250413
O	-5.511747	-1.640095	0.513948
H	-3.390523	2.242606	-0.390477
H	0.009728	-1.242548	-1.218801
H	0.293504	-3.427694	-0.229220
H	-1.690910	-4.769288	0.373673
H	-3.934252	-3.678862	0.594114
Ru	0.713077	0.076309	0.129166
O	1.149691	0.642330	-1.803922
O	0.453050	-1.299555	-2.599588
C	0.979184	-0.181214	-2.762601
C	1.476816	0.246457	-4.109641
H	1.256568	1.309058	-4.275513
H	1.036905	-0.371297	-4.900721
H	2.571238	0.124872	-4.125870
C	-0.133409	-1.428155	3.047952
C	0.828398	-0.630966	2.230465
C	0.760633	0.775357	2.178269
C	1.682233	1.547834	1.404105
C	2.692598	0.920175	0.645513
C	2.732414	-0.510309	0.637663
C	1.818538	-1.258800	1.406876
C	3.647683	1.679526	-0.238083

C	5.023571	1.690004	0.434600
C	3.181611	3.081937	-0.596803
H	3.878311	3.526889	-1.321977
H	2.180644	3.065829	-1.051794
H	3.158628	3.742660	0.283996
H	3.722217	1.093855	-1.169289
H	5.756321	2.181786	-0.222130
H	5.383075	0.671469	0.644459
H	4.989185	2.244756	1.385429
H	1.547668	2.627899	1.349797
H	-0.060677	1.286277	2.685956
H	3.431111	-1.028284	-0.021826
H	1.827282	-2.347276	1.322980
H	-0.330010	-2.408591	2.595517
H	-1.084189	-0.892331	3.171354
H	0.296645	-1.598134	4.047733
C	-5.959179	2.296358	-0.202164
H	-7.041504	2.448581	-0.135876
H	-5.599316	2.579491	-1.203037
H	-5.447276	2.900083	0.562911

**B**			
C	-2.146701	0.584027	0.111508
C	-3.386223	1.273881	-0.093567
C	-4.513483	0.551718	-0.364400
C	-4.504385	-0.962518	-0.456254
C	-3.202411	-1.633584	-0.249842
C	-2.062401	-0.859614	0.009665
C	-0.780022	-1.404420	0.203528
C	-0.697266	-2.807827	0.136189
C	-1.824932	-3.594891	-0.106711
C	-3.082989	-3.018480	-0.306341
O	-5.711774	1.040203	-0.568096

O	-1.087081	1.198004	0.397393
O	-5.533711	-1.556655	-0.687741
H	-3.387724	2.360822	-0.014424
H	1.277351	-1.747828	-1.300272
H	0.263271	-3.314139	0.269805
H	-1.719975	-4.682040	-0.146328
H	-3.964545	-3.631236	-0.503574
Ru	0.639427	0.026338	0.511116
O	0.573728	0.397707	-1.569408
O	1.286628	-1.590009	-2.267498
C	0.871742	-0.371978	-2.486513
C	0.772220	0.020877	-3.913017
H	0.531836	1.085299	-3.997569
H	-0.016513	-0.582745	-4.387976
H	1.716924	-0.208640	-4.425790
C	0.292987	-1.469512	3.460892
C	1.122652	-0.666294	2.512987
C	1.056184	0.749282	2.481163
C	1.885802	1.543238	1.624918
C	2.724144	0.926572	0.690249
C	2.737174	-0.511884	0.651222
C	1.989685	-1.288464	1.560971
C	3.547320	1.688403	-0.318173
C	4.989902	1.780668	0.186436
C	2.980188	3.056484	-0.668299
H	3.568481	3.505506	-1.481837
H	1.933849	2.983662	-1.001489
H	3.024453	3.747864	0.187613
H	3.551293	1.075082	-1.235822
H	5.626500	2.262647	-0.570560
H	5.408457	0.785943	0.401645
H	5.039520	2.380007	1.109211
H	1.774122	2.627439	1.639522
H	0.321250	1.250706	3.114845

H	3.336445	-1.015282	-0.111476
H	2.021862	-2.377286	1.487930
H	0.018674	-2.439837	3.025712
H	-0.623323	-0.929660	3.735381
H	0.867184	-1.659757	4.381642
C	-5.905872	2.444734	-0.516364
H	-6.968708	2.615213	-0.718142
H	-5.292748	2.946316	-1.280767
H	-5.646035	2.832254	0.480708

**C**			
C	1.593951	-0.451183	0.707672
C	3.009376	-0.496164	0.887613
C	3.554189	-1.576744	1.526313
C	2.710264	-2.731811	2.043117
C	1.249151	-2.634554	1.832736
C	0.734124	-1.507077	1.176929
C	-0.632439	-1.316007	0.927421
C	-1.496617	-2.327362	1.378903
C	-0.998291	-3.458297	2.026074
C	0.372853	-3.623870	2.259830
O	4.823770	-1.762990	1.771885
O	1.024454	0.515109	0.127648
O	3.246444	-3.666095	2.593206
H	3.606075	0.334907	0.512803
H	-2.575936	-2.239310	1.232015
H	-1.695572	-4.230202	2.360642
H	0.758333	-4.508673	2.769755
C	5.765562	-0.780578	1.364383
Ru	-1.025594	0.385219	-0.103506
C	-4.322378	-0.493860	-0.608003
C	-3.009097	-0.024149	-1.138209
C	-2.037953	-0.919690	-1.635022

C	-0.808193	-0.442847	-2.140025
C	-0.557284	0.957920	-2.322020
C	-1.530293	1.855403	-1.842473
C	-2.690066	1.365992	-1.198012
C	0.701498	1.456714	-2.996330
C	1.755281	0.377968	-3.217831
C	1.304271	2.686199	-2.318858
H	2.157726	3.043543	-2.912342
H	0.592757	3.518278	-2.234079
H	1.661801	2.434759	-1.310951
H	0.334158	1.772046	-3.992758
H	2.617334	0.816015	-3.740422
H	1.384872	-0.451271	-3.837493
H	2.115243	-0.031836	-2.262018
H	-0.051485	-1.173069	-2.423636
H	-2.199087	-1.996523	-1.552285
H	-1.381787	2.932704	-1.914149
H	-3.384589	2.085761	-0.762130
H	-5.052740	-0.490171	-1.432966
H	-4.259445	-1.518392	-0.219635
H	-4.704855	0.172612	0.175899
S	-1.076030	3.323315	1.286530
C	-0.985301	1.607420	1.613772
C	-2.154524	0.824186	1.683174
H	-3.118557	1.280057	1.445272
H	-2.187731	-0.005499	2.391923
H	-0.107391	1.395206	2.233006
O	0.183389	3.836347	0.799237
O	-2.326898	3.702972	0.664163
F	-1.184721	3.857542	2.807166
H	5.741273	-0.656723	0.271043
H	6.747409	-1.149924	1.678283
H	5.550416	0.181384	1.853934

TS2 (imaginary frequency: -261.13 cm^{-1})			
C	1.714607	-0.943921	0.868115
C	3.007058	-1.524826	1.056020
C	3.114996	-2.694188	1.756781
C	1.910158	-3.387929	2.363189
C	0.604182	-2.703341	2.221203
C	0.543517	-1.496258	1.518313
C	-0.651386	-0.751005	1.406164
C	-1.802877	-1.298795	2.000714
C	-1.753704	-2.522645	2.659973
C	-0.553353	-3.228477	2.788990
O	4.216768	-3.362308	1.974074
O	1.536247	0.055861	0.121041
O	2.047411	-4.441640	2.940278
H	3.860043	-1.040958	0.580422
H	-2.741705	-0.740209	1.969529
H	-2.667240	-2.924978	3.103581
H	-0.506133	-4.173425	3.332992
C	5.447872	-2.859212	1.474600
Ru	-0.464543	0.480648	-0.333022
C	-2.849880	-2.029039	-0.829754
C	-2.038362	-0.875415	-1.306256
C	-0.776977	-1.078381	-1.905050
C	-0.047479	0.019370	-2.431804
C	-0.547991	1.340399	-2.423826
C	-1.770585	1.545492	-1.718217
C	-2.496194	0.467413	-1.157593
C	0.229223	2.444732	-3.113067
C	1.489954	2.832271	-2.340878
C	-0.632466	3.658178	-3.440275
H	-0.062328	4.351646	-4.075552
H	-1.544426	3.372939	-3.986641
H	-0.922489	4.202256	-2.530524
H	0.551445	1.995548	-4.068617

H	2.087422	3.543211	-2.930724
H	2.119111	1.956738	-2.123102
H	1.220855	3.314305	-1.390567
H	0.952077	-0.161263	-2.835123
H	-0.340111	-2.078187	-1.931770
H	-2.168205	2.553274	-1.601653
H	-3.420408	0.669044	-0.614110
H	-3.408330	-2.421986	-1.695519
H	-2.217686	-2.838196	-0.442289
H	-3.580387	-1.730761	-0.068618
S	-1.160809	3.462754	0.815656
C	-0.110028	2.081251	0.940937
C	-0.564750	1.050978	1.865552
H	-1.577824	1.185526	2.255456
H	0.160027	0.820331	2.652055
H	0.931025	2.418567	0.992579
O	-0.691945	4.419813	-0.162246
O	-2.561054	3.094733	0.879823
F	-0.854836	4.155597	2.245634
H	5.416988	-2.794362	0.376337
H	6.218210	-3.572086	1.786439
H	5.656940	-1.866617	1.901474

	**D**		
C	1.597987	-0.289950	1.093326
C	2.729108	-1.131844	0.843739
C	2.670883	-2.471055	1.102398
C	1.418795	-3.115164	1.636866
C	0.288643	-2.218172	1.962997
C	0.393586	-0.811483	1.765515
C	-0.647894	0.047670	2.226934
C	-1.817388	-0.565490	2.752347
C	-1.925242	-1.931914	2.889062

C	-0.852095	-2.760624	2.521656
O	3.622285	-3.340623	0.891236
O	1.567743	0.904494	0.673435
O	1.370754	-4.316007	1.787007
H	3.595398	-0.656963	0.383113
H	-2.624752	0.085877	3.091045
H	-2.828567	-2.368998	3.318761
H	-0.897515	-3.840847	2.674830
C	4.870416	-2.897444	0.376253
Ru	-0.349773	0.404227	-0.154714
C	-2.332817	-2.309951	-0.536563
C	-1.546313	-1.190966	-1.127415
C	-0.198302	-1.376425	-1.547983
C	0.542280	-0.290777	-2.037250
C	-0.048558	1.001143	-2.249966
C	-1.417806	1.141268	-1.919312
C	-2.147664	0.083547	-1.323348
C	0.764295	2.129383	-2.849438
C	2.042240	2.419219	-2.065554
C	-0.042195	3.403149	-3.061540
H	0.589641	4.161762	-3.544909
H	-0.917482	3.237459	-3.706899
H	-0.393271	3.818534	-2.103765
H	1.058865	1.742854	-3.843219
H	2.653655	3.148431	-2.616199
H	2.653895	1.520778	-1.903574
H	1.798663	2.843277	-1.080609
H	1.609949	-0.425719	-2.226028
H	0.294132	-2.336343	-1.379383
H	-1.899596	2.113199	-2.011174
H	-3.171414	0.253875	-0.986906
H	-2.905352	-2.788752	-1.347824
H	-1.685532	-3.072762	-0.087557
H	-3.051528	-1.946164	0.208978

S	-2.377989	2.734823	0.841728
C	-0.752719	2.123803	0.993887
C	-0.493998	1.550058	2.376036
H	-1.177320	1.920389	3.153199
H	0.524414	1.803124	2.695026
H	-0.098209	2.949876	0.684243
O	-2.577312	3.494566	-0.377609
O	-3.379068	1.774997	1.268450
F	-2.374986	3.857836	2.017206
H	4.735170	-2.447490	-0.619126
H	5.503044	-3.788010	0.301735
H	5.327904	-2.165268	1.058633

**E**			
C	1.091947	-1.143318	1.128181
C	2.435779	-1.605375	1.360961
C	2.789314	-2.272120	2.492611
C	1.773858	-2.559000	3.555106
C	0.393876	-2.061396	3.319931
C	0.047212	-1.336025	2.157511
C	-1.276022	-0.867772	2.026057
C	-2.212175	-1.168896	3.021783
C	-1.860240	-1.893307	4.153836
C	-0.550172	-2.333757	4.305618
O	3.977950	-2.734720	2.788563
O	0.886414	-0.614398	0.013485
O	2.087410	-3.168049	4.553096
H	-3.237510	-0.810183	2.906714
H	-2.607978	-2.107130	4.920165
H	-0.236697	-2.896907	5.186159
C	5.043863	-2.538146	1.871915
Ru	-0.653975	0.175594	-1.090594
C	-2.804124	-1.779379	-2.806904

C	-1.764851	-0.708092	-2.800751
C	-0.393660	-1.008105	-2.970210
C	0.570229	0.027679	-2.877221
C	0.225012	1.395330	-2.709074
C	-1.163440	1.684139	-2.559032
C	-2.134767	0.658428	-2.589114
C	1.308866	2.454392	-2.652421
C	2.097309	2.390345	-1.344096
C	0.776577	3.858716	-2.908739
H	1.618397	4.560824	-2.995993
H	0.194312	3.911186	-3.841227
H	0.141292	4.202455	-2.078878
H	1.999673	2.196063	-3.473762
H	2.947447	3.087691	-1.381774
H	2.489622	1.381527	-1.150371
H	1.451993	2.676594	-0.500008
H	1.628522	-0.245762	-2.872866
H	-0.068939	-2.044802	-3.069036
H	-1.487406	2.705752	-2.359317
H	-3.177503	0.908369	-2.388039
H	-3.223578	-1.858670	-3.822898
H	-2.376650	-2.753107	-2.535155
H	-3.628804	-1.536789	-2.123310
S	-1.922370	2.694724	0.484559
C	-0.987059	1.240623	0.628391
C	-1.756170	-0.010064	0.895540
H	-1.752058	-0.759732	-0.049713
H	-2.841018	0.152028	0.952631
H	-0.118471	1.447964	1.265340
O	-1.095309	3.812774	0.084592
O	-3.202345	2.455507	-0.151836
F	-2.280309	2.980695	2.037607
H	3.146207	-1.401958	0.560155
H	4.819020	-3.025718	0.910864

H	5.927448	-2.998411	2.326499
H	5.220066	-1.463431	1.711741

**TS3 (imaginary frequency: -447.17 cm^{-1})**			
C	1.143067	-1.124102	1.150034
C	2.492085	-1.538615	1.430050
C	2.806548	-2.249825	2.546935
C	1.743995	-2.640678	3.529650
C	0.366113	-2.159215	3.256676
C	0.069611	-1.361570	2.129752
C	-1.242999	-0.877343	1.962317
C	-2.232165	-1.265574	2.876500
C	-1.935228	-2.077319	3.963800
C	-0.628259	-2.512736	4.163336
O	3.993810	-2.685793	2.886687
O	0.948174	-0.600930	0.028425
O	2.022592	-3.313601	4.496600
H	-3.252084	-0.901296	2.734122
H	-2.723298	-2.360189	4.664604
H	-0.359410	-3.134552	5.018839
C	5.102131	-2.396805	2.048362
Ru	-0.663641	0.111224	-0.987214
C	-2.904547	-1.738550	-2.760648
C	-1.848993	-0.684353	-2.720517
C	-0.475665	-1.006146	-2.909876
C	0.510724	0.009620	-2.904199
C	0.193884	1.373495	-2.719191
C	-1.175149	1.670570	-2.456490
C	-2.182485	0.675116	-2.475181
C	1.283972	2.425164	-2.740633
C	2.097952	2.417880	-1.445440
C	0.752974	3.818932	-3.053701
H	1.596299	4.508228	-3.205024

H	0.140246	3.827390	-3.967977
H	0.148971	4.212991	-2.222893
H	1.957087	2.123059	-3.561214
H	2.947196	3.112153	-1.530749
H	2.492984	1.417061	-1.218015
H	1.470601	2.742795	-0.601736
H	1.560217	-0.281953	-2.994770
H	-0.175173	-2.047866	-3.030372
H	-1.470018	2.697994	-2.241991
H	-3.214543	0.954529	-2.260632
H	-3.291725	-1.804164	-3.790199
H	-2.499924	-2.720646	-2.484374
H	-3.743904	-1.486686	-2.099603
S	-1.764778	2.781163	0.525930
C	-0.883297	1.295967	0.709826
C	-1.652278	0.089622	0.912163
H	-1.517046	-1.045282	-0.327400
H	-2.739210	0.193812	0.831291
H	0.031272	1.480155	1.285427
O	-0.908825	3.857522	0.076651
O	-3.066511	2.570172	-0.072996
F	-2.066944	3.113201	2.077873
H	3.234216	-1.280488	0.674980
H	4.956649	-2.840897	1.051567
H	5.976897	-2.844286	2.531878
H	5.239898	-1.308714	1.954226

**F**			
C	1.163995	-1.054556	1.143501
C	2.526590	-1.392447	1.458050
C	2.844834	-2.115588	2.566255
C	1.773956	-2.600123	3.498319
C	0.381120	-2.185716	3.194521

C	0.078910	-1.362504	2.087063
C	-1.249648	-0.928489	1.896403
C	-2.245452	-1.406538	2.761827
C	-1.943305	-2.252229	3.820791
C	-0.621968	-2.627622	4.050720
O	4.043366	-2.491219	2.936401
O	0.971649	-0.537406	0.016692
O	2.059692	-3.290326	4.450924
H	-3.276604	-1.083455	2.602090
H	-2.738518	-2.605787	4.480305
H	-0.349494	-3.269374	4.890155
C	5.162580	-2.108534	2.151697
Ru	-0.688912	0.050416	-0.987568
C	-3.088752	-1.585579	-2.706167
C	-1.970486	-0.597078	-2.689360
C	-0.620193	-0.997919	-2.963591
C	0.424419	-0.058311	-2.975104
C	0.199152	1.321210	-2.725287
C	-1.140229	1.703617	-2.455002
C	-2.210199	0.774886	-2.445597
C	1.352293	2.302815	-2.754690
C	2.207661	2.199292	-1.491177
C	0.903427	3.737873	-3.002152
H	1.785058	4.377422	-3.152844
H	0.268118	3.819270	-3.897181
H	0.347714	4.138439	-2.140933
H	1.976004	1.986833	-3.608665
H	3.094674	2.843320	-1.583952
H	2.545303	1.169195	-1.308340
H	1.630978	2.531606	-0.614438
H	1.447992	-0.410897	-3.125316
H	-0.394887	-2.053630	-3.123375
H	-1.362408	2.747033	-2.229795
H	-3.216337	1.119279	-2.205380

H	-3.502311	-1.632067	-3.726270
H	-2.738301	-2.589296	-2.433088
H	-3.894634	-1.281191	-2.026015
S	-1.793375	2.762400	0.511431
C	-0.915558	1.269818	0.687409
C	-1.667885	0.064433	0.882960
H	-1.251077	-1.342857	-0.550116
H	-2.754039	0.139173	0.772139
H	0.010669	1.462568	1.241384
O	-0.934050	3.837709	0.066731
O	-3.100477	2.563997	-0.078168
F	-2.076353	3.075121	2.069312
H	3.275957	-1.074955	0.733399
H	5.084128	-2.529823	1.137632
H	6.044433	-2.515977	2.657199
H	5.235145	-1.011622	2.094272

DFT mechanistic data for compound 1d

Table S16. Computed free energies (G_{tot}) of all species involved in the C–H alkenylation reaction pathway of 2,3-dimethoxy-1,4-naphthoquinone (**1d**). G_{tot} was obtained as the sum of the thermal correction to Gibbs free energy (G_{corr}) at the PBE0-D3(BJ)/bs1+CPCM(DCE) level; the electronic energy (E) at the ωB2PLYP/def2-TZVPP+CPCM(DCE) level; and the concentration correction (G_{conc}). Herein, bs1 = def2-TZVP for Ru, def2-SVP for other elements; DCE = 1,2-dichloroethane.

Species	G_{corr} (Eh)	E (Eh)	G_{conc} (Eh)	G_{tot} (Eh)
**AcOH**	0.035039	-228.813171	0.003012	-228.775120
**$C_2H_3SO_2F$**	0.025247	-725.905016	0.003012	-725.876757
**A**	0.410899	-1474.806387	0.003012	-1474.392477
**TS1**	0.407156	-1474.772670	0.003012	-1474.362502
**B**	0.411730	-1474.802489	0.003012	-1474.387747
**C**	0.406619	-1971.906829	0.003012	-1971.497198
**TS2**	0.407363	-1971.877133	0.003012	-1971.466758
**D**	0.407850	-1971.902780	0.003012	-1971.491918
**E**	0.407277	-1971.910635	0.003012	-1971.500346
**TS3**	0.404644	-1971.901957	0.003012	-1971.494302
**F**	0.405504	-1971.904097	0.003012	-1971.495581

Cartesian Coordinates for the Reaction Pathway of Compound 1d

All values are in Ångstrom

**AcOH**			
H	0.535739	-0.008153	-2.653144
O	0.535743	-0.494303	-1.811370
O	0.535743	1.594731	-1.029815
C	0.535751	0.404673	-0.823347
C	0.535744	-0.243430	0.525903
H	1.421457	-0.888024	0.627310
H	0.535741	0.522529	1.308784
H	-0.349969	-0.888024	0.627303

**$C_2H_3SO_2F$**			
C	-1.148281	-1.327974	-1.152267

C	-0.569929	-0.158549	-1.409948
H	0.396424	-0.005222	-1.897992
H	-2.119013	-1.385254	-0.651904
H	-0.652938	-2.259832	-1.437821
S	-1.348911	1.321627	-0.908869
O	-2.601467	1.067464	-0.237289
O	-0.379698	2.251473	-0.379569
F	-1.740750	1.903609	-2.360824

**A**			
C	-1.657775	0.117579	-0.479549
C	-2.510819	1.265422	-0.755185
C	-3.795841	1.303475	-0.271243
C	-4.357191	0.143866	0.509501
C	-3.523660	-1.073865	0.637346
C	-2.220762	-1.105394	0.112902
C	-1.485480	-2.290560	0.159871
C	-2.031118	-3.423774	0.762392
C	-3.308198	-3.378080	1.319779
C	-4.058376	-2.204362	1.249885
O	-4.668295	2.276564	-0.357584
O	-0.453754	0.240439	-0.761007
O	-5.470688	0.222013	0.978549
H	-0.496191	-2.334966	-0.296874
H	-1.452246	-4.349494	0.793092
H	-3.729269	-4.264995	1.798317
H	-5.069235	-2.152697	1.658580
Ru	1.471912	-0.376164	-0.303099
O	1.736099	0.155339	-2.343583
O	1.174936	-1.849855	-1.799163
C	1.379611	-0.999798	-2.720681
C	1.159139	-1.329944	-4.152020
H	1.764295	-0.680025	-4.795646

H	0.095233	-1.158091	-4.381707
H	1.384501	-2.387027	-4.340870
C	2.103108	-3.148768	1.360533
C	2.242319	-1.667285	1.252516
C	1.275752	-0.793091	1.807773
C	1.369099	0.614215	1.612577
C	2.412356	1.193284	0.857964
C	3.391947	0.301595	0.310078
C	3.320676	-1.093240	0.507201
C	2.483747	2.668747	0.548904
C	3.453586	3.335092	1.526935
C	1.123827	3.354305	0.541185
H	1.232432	4.387271	0.179705
H	0.406606	2.835526	-0.112857
H	0.691411	3.407685	1.552800
H	2.913542	2.746521	-0.464187
H	3.573638	4.399473	1.275692
H	4.447067	2.862691	1.497511
H	3.072249	3.267078	2.558121
H	0.536540	1.236828	1.943545
H	0.397942	-1.207868	2.306143
H	4.145814	0.700722	-0.372762
H	4.026214	-1.746635	-0.009385
H	2.428178	-3.644464	0.435715
H	1.068801	-3.438331	1.586256
H	2.746714	-3.507466	2.179852
C	-4.476479	3.443375	-1.151847
H	-5.417513	3.999851	-1.075360
H	-4.291312	3.170775	-2.200331
H	-3.645485	4.047768	-0.769460
O	-1.938443	2.340760	-1.333375
C	-1.527664	2.203062	-2.689624
H	-1.163686	3.189378	-3.002279

H	-2.382056	1.912825	-3.322167
H	-0.717257	1.467262	-2.790290

**TS1 (imaginary frequency: -643.59 cm^{-1})**			
C	-2.048158	0.479486	-0.115104
C	-3.317366	1.169564	-0.122538
C	-4.460026	0.448404	0.144878
C	-4.409656	-1.045638	0.397387
C	-3.102088	-1.729367	0.259537
C	-1.970830	-0.970939	-0.041701
C	-0.704147	-1.554471	-0.237384
C	-0.621019	-2.952921	-0.118438
C	-1.740514	-3.714962	0.212730
C	-2.986007	-3.110458	0.399523
O	-5.685575	0.890290	0.257944
O	-0.973355	1.123066	-0.147905
O	-5.427654	-1.636895	0.674090
O	-3.309184	2.512931	-0.232739
H	0.007104	-1.203891	-1.234618
H	0.330068	-3.453723	-0.315980
H	-1.646299	-4.798971	0.307718
H	-3.871771	-3.700683	0.641860
Ru	0.778217	0.033350	0.192792
O	1.221777	0.677222	-1.714208
O	0.374218	-1.151055	-2.626712
C	0.965810	-0.057786	-2.723875
C	1.445604	0.439350	-4.053508
H	1.296351	1.524312	-4.129041
H	0.935421	-0.082625	-4.871034
H	2.527218	0.244119	-4.123136
C	-0.113969	-1.551029	3.048655
C	0.868272	-0.741833	2.267570
C	0.815170	0.666606	2.259516

C	1.750349	1.453699	1.517639
C	2.760436	0.839650	0.746742
C	2.787408	-0.589952	0.695564
C	1.860123	-1.353159	1.434401
C	3.727395	1.617771	-0.107077
C	5.098505	1.598119	0.574968
C	3.273212	3.033609	-0.426600
H	3.977552	3.495128	-1.133832
H	2.275088	3.037043	-0.888054
H	3.248797	3.668305	0.473120
H	3.804212	1.060096	-1.055130
H	5.839521	2.103628	-0.061786
H	5.449027	0.571195	0.757358
H	5.061245	2.124941	1.541427
H	1.624659	2.535940	1.493844
H	-0.007467	1.169433	2.773254
H	3.486382	-1.094190	0.025856
H	1.859188	-2.438446	1.316245
H	-0.271525	-2.538801	2.597354
H	-1.079561	-1.032199	3.122785
H	0.271909	-1.702789	4.069099
C	-6.071069	2.235886	-0.009968
H	-7.157747	2.255506	0.129313
H	-5.823804	2.509060	-1.045332
H	-5.584859	2.927978	0.687636
C	-2.924650	3.034702	-1.500531
H	-2.912378	4.126341	-1.397272
H	-3.657033	2.746904	-2.272171
H	-1.923192	2.684876	-1.791358

**B**			
C	-2.103295	0.524463	0.149112
C	-3.336854	1.245729	-0.079763

C	-4.482906	0.528592	-0.333491
C	-4.467987	-0.987305	-0.407482
C	-3.173513	-1.677745	-0.213545
C	-2.028565	-0.914362	0.040272
C	-0.749138	-1.465131	0.239755
C	-0.676086	-2.869235	0.169689
C	-1.809604	-3.647242	-0.073587
C	-3.065543	-3.062347	-0.269677
O	-5.698455	0.981911	-0.522384
O	-1.052938	1.134527	0.467114
O	-5.501273	-1.578912	-0.628975
O	-3.316365	2.590594	0.049671
H	1.288225	-1.787252	-1.281770
H	0.280908	-3.382163	0.303148
H	-1.712538	-4.735096	-0.114026
H	-3.952137	-3.669271	-0.461935
Ru	0.672032	-0.039470	0.561583
O	0.590751	0.365956	-1.511238
O	1.291884	-1.612703	-2.246260
C	0.879386	-0.390218	-2.442731
C	0.770767	0.026824	-3.861613
H	0.523682	1.091145	-3.926036
H	-0.015837	-0.573313	-4.344289
H	1.715092	-0.186916	-4.381980
C	0.368111	-1.612115	3.477357
C	1.180535	-0.777619	2.541687
C	1.097401	0.637369	2.545897
C	1.910036	1.461804	1.702113
C	2.746779	0.879495	0.744509
C	2.774173	-0.557506	0.668916
C	2.045557	-1.365969	1.566469
C	3.548329	1.677255	-0.253577
C	4.992342	1.788271	0.242733
C	2.950617	3.040410	-0.571381

H	3.520738	3.516795	-1.382326
H	1.902152	2.953069	-0.894529
H	2.990413	3.716339	0.297010
H	3.557079	1.082980	-1.183567
H	5.614034	2.297256	-0.508954
H	5.431675	0.798011	0.435950
H	5.036588	2.370561	1.176634
H	1.784613	2.543838	1.744484
H	0.362650	1.114492	3.198237
H	3.369360	-1.035055	-0.113350
H	2.090848	-2.452166	1.466105
H	0.105277	-2.577647	3.024941
H	-0.554378	-1.090948	3.766802
H	0.950272	-1.811747	4.391068
C	-6.035410	2.364668	-0.515376
H	-7.119598	2.397347	-0.672382
H	-5.528030	2.893912	-1.334101
H	-5.776777	2.826310	0.445297
C	-2.665505	3.299229	-0.997879
H	-2.651230	4.355374	-0.702032
H	-3.224621	3.191441	-1.942199
H	-1.633091	2.946980	-1.141010

**C**			
C	1.563037	-0.653526	0.575845
C	2.996103	-0.766018	0.665687
C	3.520262	-1.874142	1.295930
C	2.626216	-2.963758	1.871293
C	1.161015	-2.790367	1.755474
C	0.674877	-1.645272	1.114535
C	-0.690007	-1.381296	0.925867
C	-1.583589	-2.336878	1.437868
C	-1.114196	-3.483538	2.078849

C	0.257036	-3.723720	2.243075
O	4.778617	-2.179481	1.475352
O	1.026194	0.325507	-0.009331
O	3.135157	-3.922973	2.404465
O	3.752746	0.176405	0.064683
H	-2.662650	-2.193310	1.341324
H	-1.834012	-4.211122	2.461462
H	0.619107	-4.623967	2.742890
C	5.861555	-1.334830	1.095121
Ru	-1.034043	0.320932	-0.120419
C	-4.390060	-0.379011	-0.458056
C	-3.081377	0.016618	-1.055570
C	-2.187322	-0.933785	-1.594551
C	-0.957789	-0.527981	-2.157134
C	-0.636916	0.856622	-2.359367
C	-1.537216	1.808841	-1.844472
C	-2.689615	1.387475	-1.142157
C	0.614856	1.279969	-3.095728
C	1.650334	0.174203	-3.271677
C	1.257271	2.539923	-2.519142
H	2.127621	2.816167	-3.131033
H	0.576893	3.402030	-2.513449
H	1.598338	2.362745	-1.489949
H	0.225248	1.532077	-4.102080
H	2.477797	0.555668	-3.886397
H	1.247203	-0.710672	-3.783936
H	2.070412	-0.132523	-2.302895
H	-0.253247	-1.299033	-2.465811
H	-2.403301	-1.999786	-1.497492
H	-1.332631	2.875616	-1.927036
H	-3.322175	2.146871	-0.679384
H	-5.160679	-0.333951	-1.244263
H	-4.363842	-1.405927	-0.071762
H	-4.694119	0.305984	0.344127

S	-0.739967	3.241882	1.242924
C	-0.815672	1.530781	1.588826
C	-2.053270	0.866731	1.702770
H	-2.975703	1.415071	1.498142
H	-2.140906	0.043508	2.414613
H	0.056352	1.235847	2.182282
O	0.553007	3.615449	0.716463
O	-1.960822	3.741707	0.646811
F	-0.750262	3.801088	2.758498
H	5.859943	-1.161162	0.012438
H	6.766080	-1.876684	1.393059
H	5.809880	-0.376635	1.630665
C	3.760350	1.463886	0.674867
H	4.358072	2.112507	0.023171
H	4.228749	1.413306	1.671385
H	2.744480	1.875240	0.763870

TS2 (imaginary frequency: -265.30 cm^{-1})

C	1.637050	-0.957032	0.733334
C	2.977154	-1.476809	0.867795
C	3.156006	-2.654066	1.560806
C	1.984896	-3.389965	2.183655
C	0.647851	-2.753828	2.113617
C	0.518541	-1.543426	1.431421
C	-0.702147	-0.835925	1.365889
C	-1.808348	-1.424149	2.005515
C	-1.693118	-2.653059	2.647048
C	-0.467103	-3.324332	2.718093
O	4.267574	-3.317388	1.750191
O	1.398355	0.017842	-0.025189
O	2.173738	-4.449579	2.735704
O	3.969272	-0.864832	0.190586
H	-2.764060	-0.895842	2.023567

H	-2.574469	-3.088196	3.123659
H	-0.369557	-4.274822	3.245310
C	5.553270	-2.826129	1.381295
Ru	-0.619754	0.426654	-0.369744
C	-3.307118	-1.767336	-0.700491
C	-2.368631	-0.746848	-1.244017
C	-1.164257	-1.137933	-1.880499
C	-0.316213	-0.164820	-2.454180
C	-0.637355	1.217598	-2.468891
C	-1.822003	1.596827	-1.779956
C	-2.665304	0.641919	-1.162032
C	0.279216	2.201173	-3.171661
C	1.532860	2.505367	-2.352386
C	-0.440191	3.477710	-3.590960
H	0.223965	4.081558	-4.226389
H	-1.353226	3.260560	-4.166107
H	-0.712254	4.090398	-2.719185
H	0.599260	1.677886	-4.089094
H	2.228371	3.119472	-2.943550
H	2.055014	1.586905	-2.048801
H	1.266310	3.065325	-1.444116
H	0.646588	-0.481653	-2.863544
H	-0.862581	-2.186709	-1.880266
H	-2.083877	2.652045	-1.703748
H	-3.546953	0.979387	-0.616096
H	-3.960989	-2.086411	-1.529069
H	-2.777695	-2.654126	-0.330667
H	-3.947107	-1.356742	0.089660
S	-1.225715	3.404913	0.781425
C	-0.204240	2.004113	0.913116
C	-0.651428	0.974007	1.838824
H	-1.663403	1.100331	2.234636
H	0.079431	0.737862	2.617705
H	0.844910	2.318471	0.935531

O	-0.770842	4.309724	-0.252325
O	-2.630119	3.073772	0.909875
F	-0.844764	4.144465	2.168263
H	5.633773	-2.717053	0.293230
H	6.262470	-3.579750	1.741475
H	5.753399	-1.863438	1.872233
C	4.340592	0.430198	0.651283
H	5.111453	0.796597	-0.037272
H	4.756654	0.371369	1.670245
H	3.484514	1.120734	0.639601

**D**			
C	1.502562	-0.296148	1.033835
C	2.605508	-1.173738	0.681006
C	2.545344	-2.513348	0.982046
C	1.310975	-3.105063	1.601023
C	0.205443	-2.192396	1.969110
C	0.324291	-0.791530	1.758402
C	-0.700721	0.086253	2.220809
C	-1.864306	-0.505961	2.780747
C	-1.980512	-1.868600	2.943822
C	-0.925883	-2.715259	2.561256
O	3.435606	-3.439377	0.727935
O	1.505990	0.907108	0.637252
O	1.247177	-4.301585	1.786213
O	3.596559	-0.649077	-0.065363
H	-2.659419	0.158623	3.122426
H	-2.877706	-2.290448	3.400833
H	-0.981271	-3.793458	2.723933
C	4.759127	-3.157291	0.283043
Ru	-0.399416	0.375523	-0.192656
C	-2.380741	-2.341677	-0.526161
C	-1.596461	-1.234733	-1.142081

C	-0.250302	-1.423948	-1.562496
C	0.490914	-0.343261	-2.064458
C	-0.101025	0.943735	-2.297276
C	-1.470712	1.086735	-1.970197
C	-2.197523	0.039270	-1.352295
C	0.715333	2.063074	-2.907960
C	1.990119	2.361254	-2.121179
C	-0.089111	3.333987	-3.141737
H	0.545899	4.085294	-3.632307
H	-0.961118	3.159962	-3.789358
H	-0.444751	3.763030	-2.191700
H	1.014652	1.661511	-3.894319
H	2.609960	3.073969	-2.683803
H	2.594227	1.463318	-1.932223
H	1.742184	2.809303	-1.148053
H	1.559243	-0.479180	-2.248513
H	0.242519	-2.382020	-1.385836
H	-1.953620	2.056662	-2.076819
H	-3.220478	0.214405	-1.015703
H	-2.994482	-2.806075	-1.315223
H	-1.731297	-3.117311	-0.103049
H	-3.063365	-1.965833	0.247331
S	-2.431462	2.725580	0.759374
C	-0.802693	2.121486	0.919637
C	-0.538713	1.591206	2.318833
H	-1.216506	1.990166	3.086409
H	0.482731	1.850032	2.622227
H	-0.151385	2.940680	0.586225
O	-2.637870	3.456717	-0.476099
O	-3.427728	1.772863	1.211794
F	-2.424721	3.874729	1.908661
H	4.752784	-2.738944	-0.730385
H	5.277439	-4.122736	0.291989
H	5.258215	-2.463342	0.974233

C	4.433221	0.324810	0.552186
H	5.132771	0.665553	-0.220211
H	4.995443	-0.128579	1.384410
H	3.848547	1.179939	0.918951

**E**			
C	1.054533	-1.034292	1.140496
C	2.441483	-1.432825	1.359611
C	2.827623	-2.055501	2.517259
C	1.803052	-2.404128	3.553415
C	0.392878	-2.006359	3.310759
C	0.019129	-1.286347	2.157903
C	-1.324662	-0.883071	2.015718
C	-2.254328	-1.256748	2.992741
C	-1.876097	-1.984616	4.114370
C	-0.544284	-2.351091	4.278942
O	4.027846	-2.426332	2.888993
O	0.833012	-0.497374	0.037738
O	2.138929	-2.997838	4.554067
H	-3.296318	-0.951977	2.872446
H	-2.620588	-2.255484	4.865658
H	-0.209303	-2.907922	5.155419
C	5.213560	-2.108530	2.166909
Ru	-0.715092	0.257917	-1.071177
C	-2.827713	-1.664403	-2.857912
C	-1.794701	-0.587859	-2.815828
C	-0.418879	-0.877849	-2.975856
C	0.536517	0.159553	-2.843362
C	0.182120	1.520965	-2.640151
C	-1.209592	1.800507	-2.508547
C	-2.175747	0.771001	-2.580306
C	1.262811	2.578450	-2.525616
C	2.027944	2.464631	-1.206530

C	0.731459	3.990557	-2.735691
H	1.571937	4.698381	-2.777464
H	0.167299	4.078825	-3.676554
H	0.077944	4.298532	-1.905721
H	1.968157	2.353040	-3.344456
H	2.881289	3.159024	-1.203996
H	2.410746	1.448294	-1.035083
H	1.367246	2.725841	-0.365953
H	1.596321	-0.107408	-2.828486
H	-0.087460	-1.910038	-3.097208
H	-1.540848	2.815296	-2.286667
H	-3.222965	1.010838	-2.391309
H	-3.234866	-1.723653	-3.880238
H	-2.397782	-2.641453	-2.602389
H	-3.661207	-1.440987	-2.178466
S	-2.099276	2.686831	0.544230
C	-1.111592	1.266043	0.668049
C	-1.832735	-0.020250	0.900475
H	-1.792737	-0.742965	-0.063474
H	-2.923517	0.097305	0.954729
H	-0.257705	1.494100	1.317795
O	-1.310175	3.842358	0.175120
O	-3.363654	2.413724	-0.109269
F	-2.480826	2.927212	2.099190
O	3.316004	-1.117029	0.380175
H	5.253952	-2.659107	1.217268
H	6.037627	-2.431281	2.813223
H	5.278843	-1.029656	1.979023
C	3.305410	-1.976111	-0.753909
H	3.636069	-2.989996	-0.475023
H	4.009642	-1.548542	-1.478105
H	2.304528	-2.029270	-1.206918

TS3 (imaginary frequency: -416.66 cm^{-1})			
C	1.094660	-1.016339	1.154496
C	2.482836	-1.378143	1.411456
C	2.833517	-2.050961	2.553115
C	1.775581	-2.475110	3.528559
C	0.368676	-2.092834	3.249035
C	0.036679	-1.306925	2.128138
C	-1.298069	-0.889845	1.951419
C	-2.271584	-1.344887	2.851528
C	-1.938467	-2.151440	3.932426
C	-0.609786	-2.511521	4.143809
O	4.025560	-2.418082	2.954445
O	0.881814	-0.480910	0.046726
O	2.085196	-3.113758	4.509919
H	-3.308983	-1.036344	2.703963
H	-2.715966	-2.486499	4.621925
H	-0.312781	-3.121726	4.998205
C	5.231586	-2.037423	2.299764
Ru	-0.740639	0.182837	-0.973238
C	-2.958689	-1.619168	-2.808076
C	-1.902345	-0.567427	-2.735983
C	-0.526034	-0.888871	-2.920965
C	0.460625	0.121771	-2.871841
C	0.144636	1.483428	-2.652480
C	-1.227850	1.780221	-2.418073
C	-2.237280	0.787071	-2.473311
C	1.242752	2.526571	-2.614731
C	2.038431	2.457462	-1.309853
C	0.726193	3.936593	-2.872889
H	1.575890	4.626642	-2.977623
H	0.129079	3.990757	-3.795867
H	0.109585	4.295174	-2.035043
H	1.924740	2.254380	-3.438621
H	2.899667	3.140428	-1.357525

H	2.412702	1.443739	-1.106767
H	1.403747	2.763532	-0.464180
H	1.511286	-0.169946	-2.947717
H	-0.226749	-1.928224	-3.063295
H	-1.523200	2.803524	-2.184940
H	-3.272279	1.064192	-2.271059
H	-3.333642	-1.666599	-3.843095
H	-2.558604	-2.606433	-2.543722
H	-3.805147	-1.377347	-2.152340
S	-1.968290	2.772129	0.574585
C	-1.032826	1.318934	0.745469
C	-1.747110	0.075903	0.916338
H	-1.547835	-1.031650	-0.359490
H	-2.837018	0.128173	0.827053
H	-0.130395	1.530571	1.330793
O	-1.147484	3.885341	0.150120
O	-3.254964	2.522315	-0.041034
F	-2.295740	3.070062	2.127937
O	3.384382	-1.008383	0.476219
H	5.326266	-2.541753	1.328579
H	6.036358	-2.369704	2.965305
H	5.275752	-0.949602	2.164285
C	3.439089	-1.830726	-0.684174
H	3.758086	-2.852301	-0.420187
H	4.180616	-1.378340	-1.354038
H	2.464438	-1.870227	-1.192146

**F**			
C	1.108502	-0.983814	1.149585
C	2.502922	-1.302181	1.427814
C	2.851963	-1.986413	2.563078
C	1.788399	-2.466401	3.507052
C	0.375779	-2.126271	3.203941

C	0.042624	-1.320721	2.096767
C	-1.300143	-0.933800	1.902246
C	-2.277298	-1.450915	2.766501
C	-1.943204	-2.283060	3.826723
C	-0.607810	-2.604541	4.061703
O	4.046943	-2.324510	2.980172
O	0.899939	-0.446093	0.040448
O	2.099701	-3.114637	4.481540
H	-3.320057	-1.167880	2.605980
H	-2.725089	-2.666106	4.485701
H	-0.310790	-3.229687	4.905181
C	5.252845	-1.880554	2.366297
Ru	-0.760775	0.118216	-0.969221
C	-3.120937	-1.490457	-2.759563
C	-2.008562	-0.496805	-2.703319
C	-0.651740	-0.884121	-2.966711
C	0.387625	0.059691	-2.930699
C	0.149942	1.433403	-2.653073
C	-1.196119	1.805136	-2.408260
C	-2.260069	0.868604	-2.434405
C	1.299823	2.419316	-2.627920
C	2.080678	2.341163	-1.315087
C	0.855371	3.847837	-2.919699
H	1.739087	4.491604	-3.036551
H	0.263104	3.911324	-3.845149
H	0.256321	4.257538	-2.092197
H	1.973524	2.096380	-3.439957
H	2.971785	2.984030	-1.370196
H	2.405320	1.316822	-1.084572
H	1.454423	2.695391	-0.481627
H	1.416919	-0.283171	-3.063201
H	-0.417560	-1.934588	-3.147199
H	-1.426765	2.842608	-2.164034
H	-3.272648	1.201463	-2.205617

H	-3.519756	-1.515025	-3.786286
H	-2.768924	-2.498530	-2.505122
H	-3.938091	-1.206675	-2.083889
S	-1.978846	2.752803	0.565902
C	-1.049561	1.290713	0.729154
C	-1.753950	0.052684	0.897035
H	-1.293344	-1.299561	-0.570212
H	-2.841492	0.083879	0.780547
H	-0.134990	1.509029	1.293018
O	-1.156214	3.861153	0.132458
O	-3.277146	2.515084	-0.028169
F	-2.273992	3.041893	2.125772
O	3.410123	-0.892016	0.515101
H	5.393411	-2.357828	1.387078
H	6.053171	-2.195186	3.045656
H	5.255546	-0.789000	2.256014
C	3.515094	-1.699381	-0.652351
H	3.839513	-2.719645	-0.389835
H	4.272298	-1.228705	-1.291338
H	2.558357	-1.745601	-1.193043

DFT mechanistic data for compound 1e

Table S17. Computed free energies (G_{tot}) of all species involved in the C–H alkenylation reaction pathway of 2-chloro-3-methoxy-1,4-naphthoquinone (**1e**). G_{tot} was obtained as the sum of the thermal correction to Gibbs free energy (G_{corr}) at the PBE0-D3(BJ)/bs1+CPCM(DCE) level; the electronic energy (E) at the ωB2PLYP/def2-TZVPP+CPCM(DCE) level; and the concentration correction (G_{conc}). Herein, bs1 = def2-TZVP for Ru, def2-SVP for other elements; DCE = 1,2-dichloroethane.

Species	G_{corr} (Eh)	E (Eh)	G_{conc} (Eh)	G_{tot} (Eh)
**AcOH**	0.035039	-228.813171	0.003012	-228.775120
**$C_2H_3SO_2F$**	0.025247	-725.905016	0.003012	-725.876757
**A**	0.369896	-1819.803854	0.003012	-1819.430947
**TS1**	0.366246	-1819.771134	0.003012	-1819.401876
**B**	0.371194	-1819.801803	0.003012	-1819.427596
**C**	0.365919	-2316.905236	0.003012	-2316.536305
**TS2**	0.366566	-2316.875432	0.003012	-2316.505854
**D**	0.368146	-2316.900437	0.003012	-2316.529279
**E**	0.366365	-2316.908465	0.003012	-2316.539088
**TS3**	0.363865	-2316.900051	0.003012	-2316.533175
**F**	0.364604	-2316.901908	0.003012	-2316.534292

Cartesian coordinates for the Reaction Pathway of Compound 1e

All values are in Ångstrom

**AcOH**			
H	0.535739	-0.008153	-2.653144
O	0.535743	-0.494303	-1.811370
O	0.535743	1.594731	-1.029815
C	0.535751	0.404673	-0.823347
C	0.535744	-0.243430	0.525903
H	1.421457	-0.888024	0.627310
H	0.535741	0.522529	1.308784
H	-0.349969	-0.888024	0.627303

**$C_2H_3SO_2F$**			
C	-1.148281	-1.327974	-1.152267

C	-0.569929	-0.158549	-1.409948
H	0.396424	-0.005222	-1.897992
H	-2.119013	-1.385254	-0.651904
H	-0.652938	-2.259832	-1.437821
S	-1.348911	1.321627	-0.908869
O	-2.601467	1.067464	-0.237289
O	-0.379698	2.251473	-0.379569
F	-1.740750	1.903609	-2.360824

**A**			
C	-1.719307	0.121915	-0.488980
C	-2.624398	1.246164	-0.695270
C	-3.916007	1.218503	-0.234295
C	-4.435938	-0.026425	0.465717
C	-3.558813	-1.212388	0.519977
C	-2.247429	-1.153165	0.021806
C	-1.466984	-2.310176	-0.003208
C	-1.979284	-3.505159	0.500646
C	-3.266461	-3.550982	1.034392
C	-4.059909	-2.404889	1.037430
O	-4.852290	2.123152	-0.264444
O	-0.522564	0.285812	-0.765165
O	-5.551573	-0.013412	0.929961
H	-0.470073	-2.285398	-0.444625
H	-1.365641	-4.408144	0.471779
H	-3.660672	-4.487147	1.435626
H	-5.079205	-2.421996	1.427519
Ru	1.425515	-0.273839	-0.284566
O	1.737911	0.307153	-2.299295
O	1.219528	-1.723402	-1.813966
C	1.433918	-0.850044	-2.712198
C	1.287140	-1.164423	-4.156304
H	1.837435	-0.439245	-4.767515

H	0.217462	-1.106631	-4.413226
H	1.630756	-2.186875	-4.359373
C	1.721389	-2.988298	1.606341
C	2.004158	-1.539358	1.389308
C	1.121901	-0.532745	1.840435
C	1.369997	0.845074	1.562995
C	2.497856	1.254258	0.822311
C	3.389969	0.230159	0.359873
C	3.151708	-1.130913	0.634479
C	2.757761	2.689195	0.436418
C	3.826880	3.260213	1.371410
C	1.507045	3.555589	0.411964
H	1.751833	4.545808	0.001226
H	0.715739	3.113256	-0.211375
H	1.102159	3.712782	1.424116
H	3.177987	2.658802	-0.583331
H	4.082079	4.286948	1.069896
H	4.748271	2.658940	1.350682
H	3.459294	3.288540	2.409315
H	0.603528	1.575087	1.825099
H	0.185703	-0.819881	2.323435
H	4.205835	0.501943	-0.313691
H	3.788762	-1.890407	0.176939
H	1.941178	-3.573887	0.702645
H	0.678384	-3.153816	1.903431
H	2.372893	-3.361030	2.412878
C	-4.766789	3.420212	-0.851300
H	-5.761010	3.857889	-0.709033
H	-4.538437	3.347875	-1.921847
H	-4.012641	4.029088	-0.337536
Cl	-1.891455	2.610563	-1.461010

TS1 (imaginary frequency: -656.45 cm^{-1})

C	-2.076700	0.519182	-0.201515
C	-3.347699	1.203406	-0.225711
C	-4.504943	0.502024	0.018265
C	-4.448381	-0.993562	0.327576
C	-3.142187	-1.677454	0.229383
C	-2.006920	-0.928835	-0.079442
C	-0.740743	-1.523221	-0.247846
C	-0.662063	-2.916759	-0.083551
C	-1.785793	-3.666145	0.262307
C	-3.031301	-3.054154	0.415403
O	-5.740450	0.904896	0.067156
O	-1.000302	1.151119	-0.265709
O	-5.469439	-1.566524	0.621385
H	-0.013643	-1.212578	-1.240040
H	0.289280	-3.425021	-0.259033
H	-1.694528	-4.746660	0.392718
H	-3.921544	-3.634616	0.664396
Ru	0.748496	0.070968	0.120179
O	1.207140	0.638680	-1.803980
O	0.373568	-1.229356	-2.646299
C	0.963704	-0.141007	-2.784464
C	1.459177	0.301259	-4.127710
H	1.324047	1.384244	-4.245067
H	0.948866	-0.245388	-4.928912
H	2.538678	0.089751	-4.181494
C	-0.142175	-1.415305	3.034337
C	0.837410	-0.631631	2.224771
C	0.782189	0.775061	2.167112
C	1.717217	1.536697	1.398385
C	2.730037	0.898144	0.652015
C	2.756188	-0.532641	0.648193
C	1.828882	-1.270674	1.412027
C	3.698393	1.646602	-0.226135
C	5.070190	1.642504	0.455028

C	3.249047	3.053610	-0.588190
H	3.955072	3.491103	-1.308843
H	2.251011	3.047341	-1.049832
H	3.227158	3.714831	0.292260
H	3.771864	1.059526	-1.156505
H	5.812193	2.125075	-0.198095
H	5.417041	0.620320	0.668449
H	5.036322	2.199001	1.404837
H	1.590409	2.617315	1.337093
H	-0.040300	1.295027	2.663727
H	3.455420	-1.059041	-0.004014
H	1.828126	-2.359404	1.330767
H	-0.306353	-2.414481	2.611355
H	-1.105177	-0.890951	3.102172
H	0.251611	-1.540787	4.055328
C	-6.216637	2.208281	-0.265996
H	-7.308341	2.126855	-0.225768
H	-5.897021	2.488465	-1.277540
H	-5.868492	2.946468	0.466784
Cl	-3.242282	2.909371	-0.466067

**B**			
C	-2.115770	0.602384	0.118208
C	-3.357216	1.314180	-0.092131
C	-4.507601	0.613955	-0.361256
C	-4.480571	-0.913314	-0.449343
C	-3.191094	-1.600599	-0.244635
C	-2.047918	-0.837546	0.015272
C	-0.767753	-1.389729	0.209466
C	-0.696666	-2.793764	0.140345
C	-1.830327	-3.571529	-0.103205
C	-3.084625	-2.985743	-0.302639
O	-5.724417	1.032820	-0.571470

O	-1.057935	1.207575	0.405420
O	-5.512941	-1.498083	-0.681227
H	1.294819	-1.746652	-1.304084
H	0.260076	-3.307152	0.273291
H	-1.733893	-4.659379	-0.143492
H	-3.970919	-3.591168	-0.500404
Ru	0.661988	0.028672	0.516061
O	0.592279	0.402471	-1.562018
O	1.296170	-1.584923	-2.270514
C	0.883449	-0.365250	-2.483267
C	0.778869	0.033747	-3.907468
H	0.524576	1.095362	-3.986076
H	-0.000181	-0.578420	-4.387190
H	1.727866	-0.179321	-4.419684
C	0.301750	-1.446250	3.474688
C	1.139914	-0.657251	2.522464
C	1.088230	0.758889	2.481934
C	1.924661	1.539205	1.620078
C	2.755120	0.908216	0.687882
C	2.753330	-0.530556	0.657428
C	1.999200	-1.293676	1.573222
C	3.583316	1.656145	-0.326670
C	5.024658	1.748982	0.181357
C	3.021066	3.022005	-0.693309
H	3.611877	3.459367	-1.511339
H	1.974907	2.948316	-1.026810
H	3.066698	3.723209	0.154463
H	3.587918	1.032654	-1.237412
H	5.664846	2.220926	-0.578907
H	5.439325	0.755198	0.408152
H	5.073597	2.357835	1.097912
H	1.823424	2.624495	1.628151
H	0.358159	1.271888	3.112040
H	3.346913	-1.044923	-0.102389

H	2.019633	-2.383158	1.506157
H	0.020431	-2.417537	3.046203
H	-0.610486	-0.896688	3.743470
H	0.872783	-1.634087	4.397873
C	-6.162676	2.388759	-0.551638
H	-7.238240	2.340250	-0.755064
H	-5.660129	2.971650	-1.333706
H	-5.988320	2.838989	0.433559
Cl	-3.232153	3.033314	0.044014

**C**			
C	1.581788	-0.443010	0.680494
C	3.011224	-0.488464	0.844621
C	3.579557	-1.565188	1.485722
C	2.704255	-2.710034	2.012779
C	1.244831	-2.618160	1.815145
C	0.729029	-1.494632	1.159857
C	-0.638910	-1.299114	0.916970
C	-1.501033	-2.308846	1.376592
C	-1.000722	-3.438174	2.024374
C	0.371862	-3.605300	2.251459
O	4.825388	-1.824626	1.752620
O	1.016026	0.520886	0.103947
O	3.241263	-3.639247	2.566304
H	-2.580934	-2.219529	1.235352
H	-1.696972	-4.208165	2.365222
H	0.759363	-4.488627	2.762095
C	5.948044	-1.005799	1.428042
Ru	-1.035936	0.400521	-0.112484
C	-4.334454	-0.475079	-0.597258
C	-3.024341	-0.004928	-1.134665
C	-2.057657	-0.899791	-1.641760
C	-0.830322	-0.422691	-2.151901

C	-0.578836	0.978445	-2.331187
C	-1.547624	1.875389	-1.842322
C	-2.704443	1.385359	-1.192771
C	0.674806	1.478241	-3.013876
C	1.729376	0.402507	-3.245249
C	1.282794	2.707967	-2.341491
H	2.124709	3.070833	-2.947953
H	0.569755	3.536958	-2.241465
H	1.660505	2.453852	-1.341883
H	0.297024	1.793187	-4.006702
H	2.579674	0.841482	-3.786081
H	1.354247	-0.434282	-3.851591
H	2.110765	0.006932	-2.292340
H	-0.075731	-1.152695	-2.441788
H	-2.219855	-1.976730	-1.561858
H	-1.397988	2.952634	-1.911819
H	-3.395713	2.104560	-0.750746
H	-5.072187	-0.459711	-1.415487
H	-4.271771	-1.503999	-0.220662
H	-4.706926	0.184470	0.197247
S	-1.070660	3.343021	1.273101
C	-0.981526	1.627037	1.602489
C	-2.150994	0.845875	1.684212
H	-3.116505	1.301658	1.452432
H	-2.178345	0.016829	2.393930
H	-0.099501	1.413795	2.215732
O	0.185523	3.849682	0.771733
O	-2.326686	3.724904	0.662947
F	-1.162387	3.877562	2.794110
H	6.035761	-0.884329	0.341256
H	6.815131	-1.551575	1.815673
H	5.869136	-0.028415	1.920034
Cl	3.860966	0.868306	0.194457

TS2 (imaginary frequency: -261.24 cm^{-1})			
C	1.707317	-0.905276	0.813515
C	3.030252	-1.455965	0.980085
C	3.193850	-2.628539	1.680121
C	1.982780	-3.343949	2.280690
C	0.660339	-2.693985	2.168233
C	0.564845	-1.484473	1.479715
C	-0.645999	-0.760267	1.387984
C	-1.776396	-1.339056	1.993974
C	-1.692891	-2.568399	2.638958
C	-0.476519	-3.251196	2.745210
O	4.268597	-3.316232	1.933590
O	1.500348	0.087797	0.074252
O	2.149224	-4.402398	2.837330
H	-2.726318	-0.799314	1.983930
H	-2.591699	-2.993922	3.090839
H	-0.402301	-4.200398	3.278375
C	5.607332	-2.933837	1.620709
Ru	-0.508913	0.490016	-0.345069
C	-2.969910	-1.947304	-0.809905
C	-2.127087	-0.821427	-1.298113
C	-0.877312	-1.068385	-1.909125
C	-0.117850	0.002852	-2.442001
C	-0.574804	1.341713	-2.432550
C	-1.791542	1.585900	-1.732087
C	-2.547170	0.533719	-1.161167
C	0.242789	2.421043	-3.115018
C	1.501519	2.774968	-2.323764
C	-0.579301	3.657734	-3.456382
H	0.019711	4.334108	-4.083441
H	-1.490872	3.397926	-4.015973
H	-0.867540	4.211049	-2.551525
H	0.566462	1.960690	-4.064661
H	2.127225	3.468867	-2.904491

H	2.102840	1.883183	-2.094186
H	1.230554	3.265198	-1.377889
H	0.875568	-0.208926	-2.845704
H	-0.472069	-2.081444	-1.933777
H	-2.157351	2.606464	-1.621449
H	-3.462940	0.766451	-0.616068
H	-3.545321	-2.325765	-1.671071
H	-2.360229	-2.774372	-0.424700
H	-3.687266	-1.624118	-0.046240
S	-1.214860	3.452572	0.825578
C	-0.154169	2.078396	0.943458
C	-0.591608	1.036246	1.863084
H	-1.604949	1.154158	2.257769
H	0.139759	0.810676	2.645124
H	0.885186	2.421815	0.987469
O	-0.762825	4.404918	-0.164791
O	-2.611250	3.074721	0.907630
F	-0.895152	4.155251	2.246779
H	5.761712	-2.920009	0.534801
H	6.234608	-3.706621	2.078280
H	5.839842	-1.953753	2.055724
Cl	4.279796	-0.588963	0.162993

**D**			
C	1.579000	-0.260254	1.056108
C	2.691697	-1.134951	0.729229
C	2.669509	-2.469194	1.048153
C	1.402703	-3.078016	1.610336
C	0.287465	-2.178509	1.963580
C	0.399185	-0.775846	1.763904
C	-0.634820	0.090736	2.230255
C	-1.800171	-0.515798	2.771837
C	-1.910992	-1.880752	2.918024

C	-0.847062	-2.715792	2.538126
O	3.576024	-3.390247	0.886412
O	1.578250	0.948113	0.685730
O	1.341665	-4.278103	1.755241
H	-2.601410	0.140008	3.116095
H	-2.809708	-2.312517	3.362459
H	-0.895307	-3.795597	2.691969
C	4.947977	-3.165167	0.563934
Ru	-0.322443	0.411488	-0.167140
C	-2.229304	-2.359369	-0.557794
C	-1.481021	-1.213845	-1.146682
C	-0.134579	-1.351929	-1.582637
C	0.570003	-0.236502	-2.063305
C	-0.062992	1.036219	-2.263875
C	-1.433536	1.128634	-1.921590
C	-2.121067	0.047463	-1.319478
C	0.706823	2.193164	-2.864697
C	1.979679	2.523128	-2.088302
C	-0.143909	3.439011	-3.068869
H	0.459497	4.218100	-3.555909
H	-1.017698	3.245026	-3.708444
H	-0.501794	3.842181	-2.108583
H	1.007803	1.818761	-3.861173
H	2.561413	3.275408	-2.639995
H	2.622946	1.646523	-1.933305
H	1.729267	2.936039	-1.100144
H	1.639692	-0.336648	-2.261498
H	0.388574	-2.298775	-1.432664
H	-1.946430	2.085391	-2.005155
H	-3.145145	0.184420	-0.968871
H	-2.824506	-2.824664	-1.360520
H	-1.556943	-3.123545	-0.149930
H	-2.927522	-2.022808	0.219766
S	-2.380478	2.738448	0.812309

C	-0.747359	2.141876	0.964644
C	-0.482158	1.594253	2.356677
H	-1.165303	1.974922	3.128645
H	0.536403	1.855098	2.667644
H	-0.101613	2.970597	0.644362
O	-2.588847	3.497065	-0.405898
O	-3.370608	1.767818	1.238943
F	-2.383026	3.859871	1.987639
H	5.058132	-2.878757	-0.489120
H	5.440763	-4.127348	0.742352
H	5.377285	-2.396396	1.218981
Cl	3.966752	-0.353011	-0.130927

**E**			
C	1.082949	-1.157321	1.070617
C	2.449366	-1.632667	1.268747
C	2.837147	-2.287248	2.406828
C	1.805090	-2.570799	3.471038
C	0.423402	-2.079089	3.268198
C	0.064454	-1.347484	2.118948
C	-1.259993	-0.873804	2.003868
C	-2.181344	-1.182024	3.011048
C	-1.815908	-1.914891	4.133673
C	-0.505098	-2.358745	4.266062
O	3.995088	-2.763076	2.770191
O	0.845858	-0.628591	-0.028828
O	2.132869	-3.185861	4.458997
H	-3.207788	-0.821708	2.912958
H	-2.554220	-2.132128	4.908077
H	-0.180149	-2.928216	5.138061
C	5.239610	-2.571467	2.100934
Ru	-0.688111	0.182869	-1.117454
C	-2.875910	-1.734251	-2.828534

C	-1.822257	-0.677298	-2.824247
C	-0.456440	-0.994585	-3.004669
C	0.522466	0.027010	-2.910614
C	0.196669	1.398585	-2.733240
C	-1.186702	1.705689	-2.574408
C	-2.172040	0.692997	-2.601238
C	1.294511	2.443157	-2.672905
C	2.067797	2.383698	-1.355134
C	0.781533	3.851325	-2.947900
H	1.632565	4.541922	-3.037364
H	0.204903	3.901213	-3.884020
H	0.146205	4.212006	-2.125144
H	1.990953	2.168382	-3.483917
H	2.925662	3.071668	-1.392205
H	2.447634	1.374057	-1.143155
H	1.417000	2.688900	-0.522000
H	1.576855	-0.261103	-2.909453
H	-0.146422	-2.035141	-3.110257
H	-1.494787	2.730574	-2.366350
H	-3.209803	0.955762	-2.390963
H	-3.302412	-1.803180	-3.842364
H	-2.460034	-2.714873	-2.563684
H	-3.693039	-1.483241	-2.138969
S	-1.925551	2.699677	0.480878
C	-0.992489	1.242354	0.610769
C	-1.759986	-0.007203	0.887930
H	-1.784962	-0.747301	-0.063814
H	-2.842524	0.159188	0.971579
H	-0.113287	1.446880	1.233915
O	-1.100191	3.814566	0.069039
O	-3.214533	2.463568	-0.138053
F	-2.261465	2.985916	2.038356
H	5.253890	-3.105733	1.142960
H	5.989557	-2.997005	2.776937

H	5.436564	-1.502893	1.947853
Cl	3.497560	-1.336784	-0.072394

TS3 (imaginary frequency: -449.39 cm^{-1})

C	1.133058	-1.156695	1.082003
C	2.503379	-1.590823	1.324146
C	2.853367	-2.282220	2.453331
C	1.774996	-2.672682	3.436089
C	0.396255	-2.189408	3.199607
C	0.088416	-1.382044	2.087862
C	-1.223390	-0.884716	1.942720
C	-2.198172	-1.274837	2.871371
C	-1.889161	-2.097771	3.947039
C	-0.582696	-2.545219	4.121417
O	4.010977	-2.727687	2.856011
O	0.906485	-0.636544	-0.026709
O	2.066969	-3.357441	4.389024
H	-3.217771	-0.902399	2.749931
H	-2.667597	-2.379617	4.658867
H	-0.303308	-3.175950	4.966723
C	5.280424	-2.391145	2.300266
Ru	-0.700732	0.113335	-1.012457
C	-2.991376	-1.683075	-2.781997
C	-1.917349	-0.648093	-2.741323
C	-0.551638	-0.991175	-2.945104
C	0.452819	0.007307	-2.938483
C	0.160642	1.375069	-2.741175
C	-1.200422	1.693050	-2.462949
C	-2.224698	0.714545	-2.477666
C	1.267541	2.409018	-2.761968
C	2.067992	2.407990	-1.458148
C	0.758911	3.806213	-3.097595
H	1.613335	4.481023	-3.251804

H	0.152430	3.811391	-4.016043
H	0.155353	4.220735	-2.276303
H	1.944745	2.087354	-3.571486
H	2.926074	3.091206	-1.545068
H	2.449728	1.407001	-1.210896
H	1.436693	2.754050	-0.625986
H	1.496701	-0.301466	-3.036222
H	-0.269709	-2.036885	-3.075689
H	-1.474617	2.723636	-2.236463
H	-3.249576	1.009636	-2.250172
H	-3.387320	-1.732188	-3.809161
H	-2.602070	-2.674655	-2.517908
H	-3.821110	-1.422089	-2.112436
S	-1.745680	2.786908	0.533185
C	-0.872346	1.293388	0.694541
C	-1.647952	0.094259	0.909842
H	-1.569763	-1.030712	-0.350851
H	-2.735410	0.209056	0.856608
H	0.056617	1.468019	1.249729
O	-0.889791	3.858394	0.072634
O	-3.059222	2.587174	-0.042987
F	-2.016916	3.113281	2.091542
H	5.417696	-2.869017	1.322485
H	6.012977	-2.784689	3.013790
H	5.388404	-1.302343	2.213928
Cl	3.598884	-1.229102	0.038472

**F**			
C	1.151678	-1.136344	1.089041
C	2.526238	-1.542798	1.351540
C	2.866247	-2.246347	2.476409
C	1.775221	-2.675004	3.430024
C	0.394325	-2.207565	3.176816

C	0.097267	-1.379236	2.077170
C	-1.214911	-0.883933	1.919955
C	-2.202789	-1.311121	2.820135
C	-1.906857	-2.162282	3.876702
C	-0.598236	-2.598388	4.068729
O	4.023129	-2.679193	2.893884
O	0.930929	-0.625683	-0.027151
O	2.060576	-3.375928	4.373154
H	-3.222794	-0.942624	2.690500
H	-2.696583	-2.472964	4.563743
H	-0.329099	-3.246562	4.904073
C	5.296981	-2.317803	2.364602
Ru	-0.708514	0.061780	-0.992179
C	-3.053306	-1.646779	-2.772711
C	-1.966109	-0.626231	-2.721120
C	-0.600699	-0.984626	-2.956622
C	0.415752	-0.006490	-2.973404
C	0.145416	1.359895	-2.734121
C	-1.205967	1.691424	-2.433778
C	-2.249636	0.733482	-2.447841
C	1.262855	2.381424	-2.769976
C	2.074555	2.377827	-1.473101
C	0.767432	3.782403	-3.110262
H	1.628702	4.446244	-3.273652
H	0.155418	3.789121	-4.025017
H	0.174287	4.208852	-2.287613
H	1.929494	2.046957	-3.582970
H	2.942711	3.046130	-1.573864
H	2.441207	1.372689	-1.220280
H	1.457334	2.742579	-0.638447
H	1.450908	-0.326411	-3.117257
H	-0.339010	-2.032676	-3.109908
H	-1.461026	2.724668	-2.197373
H	-3.267653	1.045650	-2.212714

H	-3.451101	-1.678068	-3.799813
H	-2.676089	-2.646408	-2.521764
H	-3.877513	-1.383180	-2.097599
S	-1.691395	2.806774	0.531705
C	-0.836723	1.297935	0.687920
C	-1.624568	0.120812	0.914147
H	-1.395655	-1.265319	-0.531287
H	-2.710014	0.237622	0.840828
H	0.109165	1.467192	1.215606
O	-0.829322	3.868670	0.060896
O	-3.016018	2.626378	-0.023990
F	-1.929518	3.127060	2.095505
H	5.457035	-2.779925	1.382730
H	6.022710	-2.711852	3.084782
H	5.391868	-1.226579	2.295435
Cl	3.638193	-1.146674	0.090283

DFT mechanistic data for compound 1f

Table S18. Computed free energies (G_{tot}) of all species involved in the C–H alkenylation reaction pathway of 2-chloro-1,4-naphthoquinone (**1f**). G_{tot} was obtained as the sum of the thermal correction to Gibbs free energy (G_{corr}) at the PBE0-D3(BJ)/bs1+CPCM(DCE) level; the electronic energy (E) at the ωB2PLYP/def2-TZVPP+CPCM(DCE) level; and the concentration correction (G_{conc}). Herein, bs1 = def2-TZVP for Ru, def2-SVP for other elements; DCE = 1,2-dichloroethane.

Species	G_{corr} (Eh)	E (Eh)	G_{conc} (Eh)	G_{tot} (Eh)
**AcOH**	0.035039	-228.813171	0.003012	-228.775120
**$C_2H_3SO_2F$**	0.025247	-725.905016	0.003012	-725.876757
**A**	0.339545	-1705.423773	0.003012	-1705.081216
**TS1**	0.336622	-1705.390382	0.003012	-1705.050749
**B**	0.341128	-1705.421644	0.003012	-1705.077504
**C**	0.336530	-2202.522302	0.003012	-2202.182761
**TS2**	0.336483	-2202.494426	0.003012	-2202.154931
**D**	0.338196	-2202.521084	0.003012	-2202.179877
**E**	0.336352	-2202.529139	0.003012	-2202.189775
**TS3**	0.333541	-2202.520770	0.003012	-2202.184218
**F**	0.334671	-2202.523072	0.003012	-2202.185389

Cartesian coordinates for the Reaction Pathway of Compound 1f

All values are in Ångstrom

**AcOH**			
H	0.535739	-0.008153	-2.653144
O	0.535743	-0.494303	-1.811370
O	0.535743	1.594731	-1.029815
C	0.535751	0.404673	-0.823347
C	0.535744	-0.243430	0.525903
H	1.421457	-0.888024	0.627310
H	0.535741	0.522529	1.308784
H	-0.349969	-0.888024	0.627303

**$C_2H_3SO_2F$**			
C	-1.148281	-1.327974	-1.152267

C	-0.569929	-0.158549	-1.409948
H	0.396424	-0.005222	-1.897992
H	-2.119013	-1.385254	-0.651904
H	-0.652938	-2.259832	-1.437821
S	-1.348911	1.321627	-0.908869
O	-2.601467	1.067464	-0.237289
O	-0.379698	2.251473	-0.379569
F	-1.740750	1.903609	-2.360824

		**A**	
C	-1.714848	S	-0.445647
C	-2.626215	1.253048	-0.616293
C	-3.900382	1.191486	-0.183766
C	-4.469845	-0.040721	0.452051
C	-3.573308	-1.222821	0.496191
C	-2.242919	-1.157718	0.033465
C	-1.461323	-2.315307	0.017712
C	-1.983734	-3.517018	0.492113
C	-3.285905	-3.569829	0.986799
C	-4.081922	-2.424812	0.982367
Cl	-4.958982	2.520487	-0.301513
O	-0.520183	0.334901	-0.703732
O	-5.601216	-0.056605	0.880094
H	-0.452708	-2.284263	-0.395674
H	-1.367820	-4.418561	0.470813
H	-3.690721	-4.511077	1.365093
H	-5.111045	-2.450965	1.345337
Ru	1.425343	-0.269348	-0.271103
O	1.710235	0.312969	-2.288705
O	1.187209	-1.714900	-1.799493
C	1.398526	-0.842945	-2.700023
C	1.240565	-1.156974	-4.142737
H	1.791102	-0.435353	-4.757877

H	0.169630	-1.093581	-4.393122
H	1.577076	-2.181521	-4.347079
C	1.733449	-2.986840	1.616027
C	2.022032	-1.539800	1.394933
C	1.153885	-0.527591	1.860355
C	1.406337	0.848322	1.578145
C	2.523115	1.250257	0.816710
C	3.404600	0.220837	0.345386
C	3.161196	-1.138480	0.623220
C	2.774957	2.683775	0.421119
C	3.772062	3.296392	1.407704
C	1.506069	3.516794	0.302462
H	1.750340	4.504858	-0.113560
H	0.769662	3.038426	-0.361487
H	1.032603	3.683194	1.282685
H	3.256158	2.646367	-0.570743
H	4.024299	4.322351	1.101030
H	4.704554	2.714097	1.455064
H	3.341018	3.336784	2.420519
H	0.648229	1.583219	1.851359
H	0.223791	-0.809194	2.358209
H	4.212379	0.487497	-0.339838
H	3.787611	-1.901852	0.157522
H	1.935512	-3.573630	0.708985
H	0.694339	-3.146115	1.929863
H	2.395486	-3.363575	2.412086
H	-2.209433	2.163221	-1.051021

TS1 (imaginary frequency: -582.25 cm^{-1})

C	-2.118862	0.511212	-0.226816
C	-3.405175	1.178886	-0.255969
C	-4.522999	0.465065	-0.000326
C	-4.505445	-1.015825	0.286223

C	-3.182390	-1.683753	0.195471
C	-2.040781	-0.931263	-0.101105
C	-0.768929	-1.520539	-0.251375
C	-0.685208	-2.912731	-0.083133
C	-1.811363	-3.664160	0.249958
C	-3.063135	-3.058742	0.384816
O	-1.055521	1.163736	-0.273146
O	-5.523421	-1.608496	0.556477
H	-0.024961	-1.209383	-1.228016
H	0.269540	-3.418768	-0.245498
H	-1.717422	-4.743898	0.385344
H	-3.951844	-3.645485	0.624587
Ru	0.703774	0.092304	0.115667
O	1.162030	0.660069	-1.805947
O	0.392571	-1.240692	-2.637135
C	0.953526	-0.138610	-2.780835
C	1.455868	0.302338	-4.121872
H	1.314384	1.383586	-4.246196
H	0.956305	-0.252715	-4.924143
H	2.537043	0.096878	-4.166167
C	-0.187472	-1.377649	3.041212
C	0.791480	-0.597856	2.227457
C	0.737298	0.808511	2.160399
C	1.672997	1.564836	1.387227
C	2.686902	0.921053	0.646588
C	2.710464	-0.509627	0.650048
C	1.782376	-1.241954	1.418508
C	3.658392	1.663086	-0.233232
C	5.029962	1.652744	0.448753
C	3.217092	3.071962	-0.597544
H	3.926914	3.504627	-1.317332
H	2.220051	3.071095	-1.061312
H	3.197422	3.734184	0.282190
H	3.729249	1.073650	-1.162317

H	5.774251	2.130361	-0.205340
H	5.371632	0.629216	0.663935
H	4.998732	2.211238	1.397434
H	1.547918	2.645305	1.320462
H	-0.084835	1.332249	2.653675
H	3.409693	-1.040834	0.001764
H	1.781194	-2.331134	1.343845
H	-0.364399	-2.372560	2.613074
H	-1.144803	-0.845227	3.122551
H	0.215858	-1.514113	4.057075
H	-3.429838	2.256011	-0.429545
Cl	-6.059422	1.194008	0.047017

**B**			
C	-2.144519	0.632788	0.041663
C	-3.390170	1.336456	-0.202441
C	-4.502598	0.623271	-0.481660
C	-4.522975	-0.884094	-0.557235
C	-3.227939	-1.564019	-0.317240
C	-2.080383	-0.803114	-0.043404
C	-0.805967	-1.358886	0.182851
C	-0.738699	-2.764126	0.131380
C	-1.872343	-3.536965	-0.123698
C	-3.121896	-2.948932	-0.354539
O	-1.097496	1.249384	0.343964
O	-5.549478	-1.477604	-0.800649
H	1.264985	-1.741903	-1.304328
H	0.212193	-3.281525	0.287483
H	-1.781431	-4.625801	-0.148581
H	-4.005055	-3.556167	-0.560950
Ru	0.618827	0.061197	0.495917
O	0.591249	0.416332	-1.583192
O	1.280035	-1.584022	-2.271049

C	0.885886	-0.360729	-2.496135
C	0.804594	0.031283	-3.923492
H	0.579693	1.098820	-4.011497
H	0.011772	-0.561960	-4.404700
H	1.750506	-0.213147	-4.426998
C	0.213277	-1.380960	3.468313
C	1.066260	-0.605538	2.518237
C	1.021519	0.810501	2.461273
C	1.876627	1.577624	1.605832
C	2.717730	0.932966	0.693365
C	2.705688	-0.506240	0.675756
C	1.935538	-1.256374	1.588493
C	3.569897	1.664317	-0.313035
C	5.007405	1.729278	0.210465
C	3.038556	3.040520	-0.686056
H	3.648543	3.466769	-1.495766
H	1.995784	2.987576	-1.033882
H	3.086109	3.740048	0.162996
H	3.572383	1.039820	-1.223135
H	5.664255	2.188307	-0.543377
H	5.400425	0.727719	0.441550
H	5.058323	2.337396	1.127336
H	1.783820	2.663710	1.603782
H	0.286037	1.333645	3.076538
H	3.305758	-1.031627	-0.071392
H	1.951574	-2.346499	1.533015
H	-0.068742	-2.354743	3.046081
H	-0.698309	-0.824211	3.724013
H	0.774566	-1.563639	4.398497
H	-3.399358	2.426143	-0.144192
Cl	-6.001803	1.383028	-0.769547

C

C	1.675971	-0.257883	0.798846
C	3.107071	-0.242670	1.014187
C	3.673358	-1.285600	1.661898
C	2.888712	-2.469987	2.182400
C	1.426095	-2.436331	1.944998
C	0.868158	-1.345793	1.258974
C	-0.502985	-1.218396	0.983044
C	-1.328682	-2.254664	1.450150
C	-0.788573	-3.347202	2.126795
C	0.587145	-3.451917	2.379579
O	1.088355	0.679501	0.205143
O	3.447690	-3.376951	2.754589
H	-2.408756	-2.215902	1.293514
H	-1.456085	-4.140220	2.472494
H	1.000418	-4.310013	2.912706
Ru	-0.945670	0.434468	-0.100038
C	-4.060176	-0.899685	-0.651846
C	-2.820034	-0.242421	-1.159711
C	-1.733316	-0.983897	-1.681860
C	-0.586258	-0.328473	-2.164907
C	-0.539106	1.099039	-2.337917
C	-1.629009	1.836475	-1.852392
C	-2.703454	1.178155	-1.203526
C	0.610260	1.743927	-3.078107
C	1.995742	1.249465	-2.662951
C	0.554077	3.265664	-3.074986
H	1.401596	3.664026	-3.650361
H	-0.367130	3.650955	-3.535547
H	0.623680	3.662941	-2.050095
H	0.440182	1.399072	-4.117327
H	2.735045	1.606299	-3.393709
H	2.070142	0.153474	-2.626108
H	2.267888	1.644770	-1.675528
H	0.268518	-0.935705	-2.466097

H	-1.740601	-2.074238	-1.622117
H	-1.642203	2.923474	-1.914524
H	-3.496880	1.787195	-0.767279
H	-4.809511	-0.898854	-1.459432
H	-3.877993	-1.942701	-0.364427
H	-4.489031	-0.353940	0.198945
S	-1.234957	3.426869	1.104005
C	-1.015976	1.747500	1.546682
C	-2.115111	0.875680	1.667088
H	-3.115053	1.231559	1.407898
H	-2.075651	0.088353	2.422122
H	-0.125295	1.643659	2.175111
O	-0.028333	3.979965	0.532216
O	-2.527907	3.675573	0.502445
F	-1.328390	4.059046	2.584517
H	3.691285	0.603212	0.648567
Cl	5.349497	-1.350109	1.949841

**TS2 (imaginary frequency: -253.53 cm^{-1})**			
C	1.724601	-0.932396	0.880297
C	3.035318	-1.514328	1.083710
C	3.137684	-2.671374	1.774577
C	1.950285	-3.377981	2.380139
C	0.640238	-2.694884	2.234804
C	0.567684	-1.487613	1.529407
C	-0.633973	-0.748443	1.409583
C	-1.782796	-1.308257	2.000748
C	-1.722352	-2.531071	2.658439
C	-0.515022	-3.227825	2.795310
O	1.556980	0.048074	0.115127
O	2.082334	-4.432015	2.957574
H	-2.726615	-0.758429	1.968673
H	-2.633601	-2.943476	3.097517

H	-0.465307	-4.172495	3.339460
Ru	-0.447626	0.474897	-0.334561
C	-2.839331	-2.033120	-0.829593
C	-2.027278	-0.880203	-1.305742
C	-0.768003	-1.083348	-1.908736
C	-0.038338	0.014046	-2.435679
C	-0.537627	1.335775	-2.425749
C	-1.758067	1.541154	-1.717279
C	-2.481360	0.463705	-1.152506
C	0.239006	2.440105	-3.114967
C	1.503395	2.822481	-2.346052
C	-0.621623	3.655955	-3.435704
H	-0.052414	4.349317	-4.071832
H	-1.536335	3.373920	-3.979085
H	-0.906629	4.199028	-2.523780
H	0.556675	1.992195	-4.072623
H	2.099056	3.535234	-2.935378
H	2.132843	1.945470	-2.135140
H	1.238977	3.300891	-1.392562
H	0.960049	-0.167638	-2.841502
H	-0.331546	-2.083340	-1.937196
H	-2.155066	2.548972	-1.598936
H	-3.402766	0.666379	-0.604616
H	-3.399078	-2.422985	-1.695988
H	-2.207892	-2.844445	-0.445477
H	-3.569239	-1.735305	-0.067745
S	-1.148796	3.458690	0.816728
C	-0.100411	2.073176	0.940945
C	-0.561841	1.040691	1.864075
H	-1.578890	1.177826	2.242969
H	0.155911	0.821192	2.660711
H	0.940306	2.410310	1.002814
O	-0.671116	4.417014	-0.154989
O	-2.549303	3.091373	0.871710

F	-0.847275	4.141404	2.251123
H	3.901011	-1.030548	0.628087
Cl	4.634996	-3.448395	2.000005

**D**			
C	1.544484	-0.328996	0.856115
C	2.567569	-1.241305	0.359361
C	2.485792	-2.568270	0.576199
C	1.330543	-3.190834	1.289976
C	0.294849	-2.258427	1.798873
C	0.428774	-0.843891	1.657488
C	-0.553925	0.022204	2.223919
C	-1.688977	-0.577948	2.831837
C	-1.813928	-1.944396	2.941196
C	-0.803900	-2.787491	2.444230
O	1.609172	0.908447	0.580965
O	1.248863	-4.391911	1.427380
H	-2.449113	0.080229	3.255386
H	-2.684099	-2.375927	3.439576
H	-0.873122	-3.871513	2.554850
Ru	-0.322505	0.385161	-0.197935
C	-2.415512	-2.255933	-0.551388
C	-1.630474	-1.147076	-1.160085
C	-0.317837	-1.351086	-1.663371
C	0.429627	-0.268902	-2.159020
C	-0.137768	1.039421	-2.313022
C	-1.476312	1.207864	-1.892797
C	-2.193085	0.157763	-1.265998
C	0.669811	2.158583	-2.932729
C	1.977245	2.421506	-2.188298
C	-0.125031	3.444456	-3.110214
H	0.497289	4.192361	-3.621466
H	-1.030320	3.291713	-3.716120

H	-0.425557	3.866570	-2.138363
H	0.925437	1.771882	-3.937011
H	2.582628	3.143268	-2.754925
H	2.578221	1.511789	-2.050331
H	1.771737	2.844548	-1.194262
H	1.477344	-0.429351	-2.424449
H	0.153307	-2.330973	-1.561183
H	-1.936648	2.193482	-1.936976
H	-3.188491	0.347130	-0.861180
H	-3.081810	-2.666914	-1.327471
H	-1.770705	-3.067988	-0.194909
H	-3.047949	-1.891920	0.269007
S	-2.315585	2.710986	0.941190
C	-0.680708	2.091866	0.998774
C	-0.361652	1.518251	2.368324
H	-1.000197	1.903164	3.175373
H	0.675441	1.752212	2.635503
H	-0.041872	2.919119	0.661340
O	-2.581146	3.482969	-0.256712
O	-3.290769	1.748119	1.415645
F	-2.230273	3.819119	2.123207
H	3.394361	-0.794185	-0.195591
Cl	3.674179	-3.650245	0.007656

**E**			
C	1.094097	-1.167575	1.091350
C	2.453168	-1.647236	1.305679
C	2.806834	-2.316665	2.418119
C	1.820654	-2.611295	3.496002
C	0.438375	-2.101682	3.280913
C	0.073956	-1.362750	2.130976
C	-1.251381	-0.889864	2.019038
C	-2.175059	-1.202944	3.020943

C	-1.806688	-1.939115	4.140666
C	-0.494818	-2.382055	4.273840
O	0.892002	-0.639178	-0.018983
O	2.137661	-3.228379	4.487634
H	-3.201858	-0.843528	2.923614
H	-2.544377	-2.160643	4.914540
H	-0.173665	-2.954674	5.145330
Ru	-0.651960	0.166763	-1.092603
C	-2.863909	-1.737192	-2.795744
C	-1.805303	-0.685472	-2.795763
C	-0.442563	-1.007779	-2.988767
C	0.540841	0.009591	-2.901466
C	0.221812	1.382766	-2.722061
C	-1.158131	1.694831	-2.549796
C	-2.146994	0.685750	-2.566385
C	1.324445	2.422166	-2.676900
C	2.141585	2.327499	-1.388011
C	0.811562	3.838480	-2.904012
H	1.663754	4.526553	-3.000981
H	0.210516	3.912934	-3.822944
H	0.200534	4.182883	-2.056361
H	1.991480	2.161185	-3.516902
H	3.002246	3.010887	-1.437698
H	2.522408	1.310340	-1.216145
H	1.521850	2.614534	-0.525069
H	1.594080	-0.282318	-2.916395
H	-0.137859	-2.049152	-3.102090
H	-1.460405	2.721079	-2.340033
H	-3.181848	0.952378	-2.346937
H	-3.297182	-1.799794	-3.807138
H	-2.451266	-2.720919	-2.537649
H	-3.675330	-1.484343	-2.100202
S	-1.882006	2.692395	0.506186
C	-0.958991	1.226990	0.633799

C	-1.737174	-0.016963	0.903425
H	-1.752973	-0.760807	-0.049750
H	-2.819989	0.154193	0.973231
H	-0.082280	1.424383	1.262986
O	-1.046470	3.802580	0.103552
O	-3.169617	2.467047	-0.119077
F	-2.220701	2.970605	2.063679
H	3.167278	-1.443762	0.506126
Cl	4.391654	-2.885534	2.672195

TS3 (imaginary frequency: -453.18 cm^{-1})

C	1.154639	-1.110207	1.145477
C	2.522495	-1.523044	1.422514
C	2.837189	-2.223255	2.528672
C	1.800362	-2.622214	3.524472
C	0.414650	-2.153131	3.253279
C	0.099388	-1.357488	2.128146
C	-1.222757	-0.893955	1.961325
C	-2.204061	-1.303629	2.872993
C	-1.888936	-2.111408	3.958422
C	-0.573779	-2.523916	4.158506
O	0.965028	-0.599586	0.022061
O	2.083585	-3.285707	4.496392
H	-3.230708	-0.958078	2.733127
H	-2.670622	-2.409992	4.659874
H	-0.296165	-3.142984	5.013091
Ru	-0.646434	0.105099	-0.984215
C	-2.913219	-1.722432	-2.756249
C	-1.848910	-0.677594	-2.717260
C	-0.479146	-1.009206	-2.915353
C	0.515111	-0.002237	-2.907738
C	0.208958	1.365193	-2.720129
C	-1.155689	1.672682	-2.453527

C	-2.169562	0.683412	-2.464119
C	1.306962	2.407893	-2.742448
C	2.132140	2.382161	-1.454338
C	0.785067	3.808684	-3.038186
H	1.633196	4.491883	-3.189738
H	0.165339	3.830936	-3.947451
H	0.191091	4.200061	-2.198884
H	1.970271	2.106582	-3.571322
H	2.984693	3.072050	-1.540715
H	2.523944	1.377165	-1.240221
H	1.514632	2.702929	-0.601652
H	1.561967	-0.301919	-3.002565
H	-0.187400	-2.052898	-3.040918
H	-1.441552	2.702307	-2.237307
H	-3.198064	0.969772	-2.241932
H	-3.304319	-1.780038	-3.784840
H	-2.516094	-2.709133	-2.485754
H	-3.748048	-1.465287	-2.091577
S	-1.766191	2.767352	0.530658
C	-0.878928	1.283965	0.715002
C	-1.640884	0.073984	0.917251
H	-1.500942	-1.055396	-0.332199
H	-2.728520	0.168969	0.834189
H	0.034100	1.472815	1.291912
O	-0.912201	3.844517	0.080976
O	-3.066122	2.549712	-0.068917
F	-2.068069	3.095751	2.082130
H	3.272778	-1.258497	0.675634
Cl	4.429614	-2.728252	2.860943

**F**			
C	1.183495	-0.998961	1.172722
C	2.565740	-1.323293	1.494120

C	2.883388	-2.025384	2.598086
C	1.837142	-2.514986	3.543650
C	0.435711	-2.126433	3.230275
C	0.114594	-1.317230	2.115799
C	-1.224857	-0.917389	1.913400
C	-2.213005	-1.418654	2.772918
C	-1.891896	-2.248482	3.838868
C	-0.561846	-2.587262	4.081554
O	0.999348	-0.503642	0.039883
O	2.126833	-3.184358	4.509522
H	-3.251589	-1.124700	2.605933
H	-2.680659	-2.619491	4.496554
H	-0.280767	-3.217338	4.926881
Ru	-0.657814	0.061135	-0.970375
C	-3.019181	-1.634676	-2.706376
C	-1.920693	-0.624958	-2.683611
C	-0.560853	-1.001050	-2.940625
C	0.465569	-0.040647	-2.951912
C	0.211173	1.335888	-2.716066
C	-1.136362	1.692291	-2.449127
C	-2.187735	0.742053	-2.441040
C	1.344821	2.339194	-2.746872
C	2.202914	2.251996	-1.483653
C	0.868948	3.765204	-2.995161
H	1.738764	4.420543	-3.146312
H	0.232574	3.834316	-3.890440
H	0.306106	4.155865	-2.134089
H	1.973624	2.033277	-3.600788
H	3.076778	2.913431	-1.577494
H	2.562595	1.229121	-1.301842
H	1.620821	2.573163	-0.606248
H	1.496832	-0.374239	-3.092763
H	-0.313830	-2.053304	-3.090836
H	-1.380412	2.732124	-2.229912

H	-3.201674	1.067185	-2.206540
H	-3.423437	-1.688724	-3.729893
H	-2.651315	-2.631469	-2.431070
H	-3.836249	-1.345137	-2.033190
S	-1.816568	2.760178	0.509480
C	-0.917781	1.280861	0.700824
C	-1.653852	0.065813	0.897289
H	-1.224288	-1.330435	-0.531719
H	-2.740044	0.121891	0.776705
H	0.000075	1.489962	1.263105
O	-0.968683	3.843597	0.063882
O	-3.115069	2.536380	-0.089380
F	-2.115178	3.076505	2.062655
H	3.323961	-0.997336	0.780309
Cl	4.491275	-2.435052	2.982265

Table S19. Computed Gibbs Free Energies (in kcal/mol) for the C–H Alkenylation Pathway of Compounds **3a**, **3b**, **3c**, **3d**, **3e** and **3f**.

Species	Compound					
	**3a**	**3b**	**3c**	**3d**	**3e**	**3f**
**TS1**	18.25	18.01	18.51	18.81	18.24	19.12
**B**	2.31	2.12	2.61	2.97	2.1	2.33
**C**	-3.88	-1.09	-1.91	-1.94	-2.33	0.06
**TS2**	16.53	15.94	16.91	17.17	16.77	17.52
**D**	1.32	0.93	1.92	1.38	2.07	1.87
**E**	-5.17	-5.03	-4.21	-3.91	-4.08	-4.34
**TS3**	-1.54	-1.65	-0.56	-0.12	-0.37	-0.86
**F**	-2.27	-2.13	-1.38	-0.92	-1.07	-1.59

Comparison of global and local energy barriers

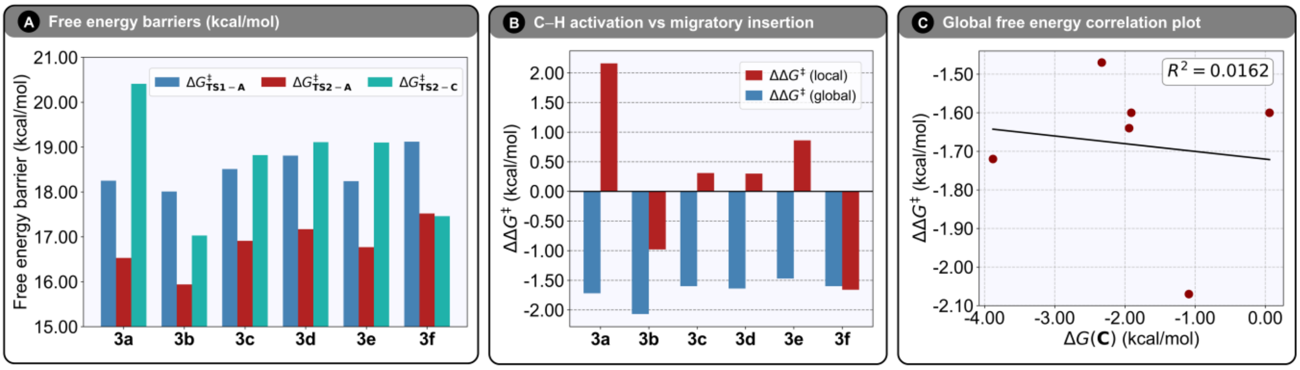

Figure S8. **(A)** Comparison of global and local free energy barriers (in kcal/mol) for the C–H activation and olefin insertion steps, as a function of the substituent on the B-ring of 1,4-naphthoquinone. **(B)** Comparison of global and local free energy differences ($\Delta\Delta G^{\ddagger}$(global) $= \Delta G^{\ddagger}_{\mathbf{TS2-A}} - \Delta G^{\ddagger}_{\mathbf{TS1-A}}$; $\Delta\Delta G^{\ddagger}$(local) $= \Delta G^{\ddagger}_{\mathbf{TS2-C}} - \Delta G^{\ddagger}_{\mathbf{TS1-A}}$) as a function of the B-ring substituent. **(E)** Correlation between $\Delta\Delta G^{\ddagger}$(global) and the computed Gibbs free energy of intermediate **C**.

r^2SCAN-3c mechanistic data for compound 1a

Table S20. Computed free energies (G_{tot}) of all species involved in the C–H alkenylation reaction pathway of menadione (**1a**). G_{tot} was obtained as the sum of the thermal correction to Gibbs free energy (G_{corr}) at the r^2SCAN-3c/CPCM(DCE) level; the electronic energy (E) at the CCSD(T)/def2-TZVPP+CPCM(DCE) level; and the concentration correction (G_{conc}). Herein, DCE = 1,2-dichloroethane.

Species	G_{corr} (Eh)	E (Eh)	G_{conc} (Eh)	G_{tot} (Eh)
**AcOH**	0.033687	-228.766529	0.003012	-228.729830
**$C_2H_3SO_2F$**	0.023936	-725.571464	0.003012	-725.544516
**A**	0.370161	-1285.047521	0.003012	-1284.674348
**TS1**	0.367199	-1285.013689	0.003012	-1284.643478
**B**	0.371341	-1285.045191	0.003012	-1284.670838
**C**	0.365394	-1781.867549	0.003012	-1781.499143
**TS2**	0.365976	-1781.832223	0.003012	-1781.463235
**D**	0.367486	-1781.859314	0.003012	-1781.488816
**E**	0.365831	-1781.862590	0.003012	-1781.493747
**TS3**	0.363337	-1781.855109	0.003012	-1781.488760
**F**	0.364617	-1781.860911	0.003012	-1781.493283

Cartesian Coordinates of the r^2SCAN-3c Optimized Reaction Pathway for Compound 1a

All values are in Ångstrom

**AcOH**			
H	0.535743	-0.018156	-2.662381
O	0.535743	-0.499869	-1.817514
O	0.535744	1.606810	-1.025341
C	0.535744	0.410271	-0.817808
C	0.535744	-0.243812	0.529142
H	1.417465	-0.884994	0.627262
H	0.535743	0.514743	1.311003
H	-0.345977	-0.884994	0.627261

**$C_2H_3SO_2F$**			
C	-1.143425	-1.338643	-1.147533

C	-0.574119	-0.174486	-1.421717
H	0.379372	-0.020529	-1.920213
H	-2.098119	-1.406677	-0.634860
H	-0.644773	-2.259428	-1.435917
S	-1.352639	1.327006	-0.901847
O	-2.625443	1.087112	-0.238895
O	-0.368383	2.255290	-0.362782
F	-1.737035	1.937697	-2.372719

**A**			
C	-1.751121	0.311496	-0.417503
C	-2.647261	1.411154	-0.725731
C	-3.943008	1.426664	-0.347797
C	-4.505762	0.258131	0.386803
C	-3.638480	-0.929627	0.584110
C	-2.292830	-0.919832	0.163004
C	-1.525178	-2.079288	0.284975
C	-2.077463	-3.226855	0.842987
C	-3.396714	-3.224257	1.289275
C	-4.176723	-2.079848	1.154157
C	-4.851711	2.575554	-0.600093
O	-0.536573	0.501765	-0.672844
O	-5.660988	0.287007	0.794624
H	-2.197461	2.261950	-1.231924
H	-0.504657	-2.095727	-0.080178
H	-1.475776	-4.126972	0.925293
H	-3.823712	-4.120098	1.729868
H	-5.212634	-2.071720	1.477657
H	-5.729248	2.243945	-1.166917
H	-5.231854	2.971879	0.348440
H	-4.340870	3.369285	-1.147584
Ru	1.379096	-0.132309	-0.171514
O	1.719760	0.387763	-2.222592

O	1.161928	-1.636891	-1.677777
C	1.428501	-0.794010	-2.602983
C	1.428002	-1.183877	-4.037987
H	0.695730	-1.973640	-4.217888
H	2.421594	-1.574610	-4.288128
H	1.229189	-0.316310	-4.669764
C	1.569551	-2.740054	1.932112
C	1.916887	-1.323234	1.609620
C	1.092697	-0.244022	1.999651
C	1.413623	1.101257	1.635357
C	2.556594	1.396887	0.860879
C	3.395223	0.298430	0.462910
C	3.079826	-1.028366	0.821201
C	2.890085	2.787064	0.380879
C	3.903145	3.406990	1.358364
C	1.668066	3.685695	0.198786
H	1.974953	4.622464	-0.275365
H	0.912860	3.210259	-0.436823
H	1.207043	3.939228	1.159543
H	3.383786	2.669966	-0.593461
H	4.207572	4.394145	0.997087
H	4.798251	2.784377	1.456904
H	3.452775	3.525616	2.350270
H	0.693677	1.882030	1.855605
H	0.152574	-0.447497	2.503634
H	4.218727	0.483433	-0.221083
H	3.665738	-1.846194	0.412040
H	1.690644	-3.381260	1.053330
H	0.548728	-2.824775	2.309041
H	2.256677	-3.105694	2.704150

**TS1 (imaginary frequency: -492.39 cm^{-1})**			
C	-2.083780	0.480439	-0.292412

C	-3.346230	1.172187	-0.366426
C	-4.503996	0.523916	-0.096141
C	-4.492559	-0.935616	0.253539
C	-3.194598	-1.656155	0.220603
C	-2.034037	-0.945203	-0.094236
C	-0.769349	-1.565466	-0.202386
C	-0.718910	-2.952886	0.022653
C	-1.865621	-3.658963	0.372618
C	-3.105454	-3.023408	0.466680
C	-5.827804	1.198233	-0.103363
O	-0.989159	1.111834	-0.376168
O	-5.537188	-1.505137	0.540826
H	-3.328370	2.235734	-0.591220
H	-0.088969	-1.301803	-1.232445
H	0.220344	-3.484209	-0.106041
H	-1.798938	-4.727848	0.553460
H	-4.000331	-3.584493	0.717075
H	-5.731542	2.252310	-0.368556
H	-6.498805	0.700353	-0.813127
H	-6.303828	1.109153	0.879950
Ru	0.736341	0.012886	0.083676
O	1.244557	0.500870	-1.875784
O	0.231277	-1.321181	-2.683570
C	0.905998	-0.274938	-2.842072
C	1.384978	0.108244	-4.213736
H	2.204344	-0.565490	-4.487991
H	1.745642	1.136930	-4.232875
H	0.579965	-0.033579	-4.938458
C	0.009765	-1.579843	3.031466
C	0.911482	-0.711779	2.215542
C	0.739931	0.685176	2.174857
C	1.620622	1.526340	1.416629
C	2.683760	0.977781	0.671958
C	2.807275	-0.451312	0.637144

C	1.940026	-1.271770	1.385751
C	3.617051	1.811068	-0.166121
C	4.941853	1.956678	0.605576
C	3.053842	3.173806	-0.562052
H	3.743376	3.659922	-1.258359
H	2.081041	3.076970	-1.054523
H	2.946112	3.831852	0.307152
H	3.816239	1.235172	-1.079798
H	5.662591	2.507079	-0.006984
H	5.373598	0.981814	0.853748
H	4.782396	2.512891	1.535921
H	1.415750	2.589961	1.373443
H	-0.108482	1.132022	2.685408
H	3.538534	-0.908480	-0.022411
H	2.022337	-2.350599	1.296116
H	-0.004401	-2.604655	2.655860
H	-1.010004	-1.185032	3.046797
H	0.381328	-1.601775	4.062799

**B**			
C	-1.749526	-0.072129	-1.195542
C	-2.844523	-0.485670	-2.039279
C	-4.122732	-0.400433	-1.599912
C	-4.422355	0.126630	-0.226566
C	-3.284679	0.541181	0.628244
C	-1.978950	0.408020	0.132743
C	-0.823109	0.749651	0.864175
C	-1.048216	1.264391	2.153924
C	-2.340733	1.420871	2.652827
C	-3.465094	1.056937	1.904799
C	-5.289542	-0.806789	-2.427754
O	-0.549340	-0.103962	-1.608926
O	-5.584974	0.204819	0.156923

H	-2.609221	-0.855779	-3.034319
H	0.868873	-0.615081	2.270252
H	-0.213621	1.547497	2.791693
H	-2.476859	1.830132	3.650406
H	-4.465768	1.174939	2.308566
H	-4.970864	-1.170035	-3.406373
H	-5.861783	-1.589362	-1.916215
H	-5.975702	0.038124	-2.556503
Ru	0.890764	0.358920	-0.159542
O	0.594004	-1.707221	0.262298
O	0.764099	-1.564564	2.499206
C	0.587529	-2.243204	1.382103
C	0.371150	-3.700239	1.561712
H	0.347840	-4.199990	0.594668
H	-0.580610	-3.849614	2.083803
H	1.161595	-4.113773	2.194787
C	0.810013	3.693891	0.109943
C	1.600403	2.443096	-0.107419
C	1.780712	1.892991	-1.404049
C	2.612529	0.748346	-1.646673
C	3.164914	0.044998	-0.575758
C	2.885251	0.525921	0.754888
C	2.171592	1.721294	0.986547
C	3.959222	-1.227923	-0.728343
C	5.455332	-0.898145	-0.589267
C	3.674481	-1.981824	-2.025253
H	4.189509	-2.946818	-2.006728
H	2.602289	-2.165978	-2.152886
H	4.040015	-1.430371	-2.898378
H	3.676206	-1.872858	0.116580
H	6.044000	-1.819845	-0.632356
H	5.670889	-0.395981	0.359460
H	5.776820	-0.243833	-1.407323
H	2.715557	0.383650	-2.662247

H	1.266761	2.355786	-2.241811
H	3.246919	-0.046005	1.605859
H	2.004361	2.059348	2.004520
H	0.320686	3.686625	1.087097
H	0.053315	3.818929	-0.668720
H	1.484467	4.557751	0.074084

**C**			
C	1.658572	-0.636814	0.783655
C	3.082663	-0.780777	0.943750
C	3.593741	-1.913128	1.484304
C	2.688113	-3.033187	1.921516
C	1.225794	-2.859813	1.749526
C	0.756765	-1.663686	1.185044
C	-0.604396	-1.389262	0.972772
C	-1.513656	-2.382250	1.364966
C	-1.060035	-3.577049	1.919352
C	0.303968	-3.827743	2.117565
C	5.051061	-2.125637	1.679413
O	1.150705	0.415736	0.278298
O	3.169183	-4.051197	2.403091
H	3.721912	0.037767	0.622713
H	-2.582848	-2.230939	1.244012
H	-1.784877	-4.332774	2.209374
H	0.639099	-4.763105	2.554508
H	5.265952	-2.310312	2.738360
H	5.376583	-3.023813	1.141792
H	5.625344	-1.264341	1.334408
Ru	-0.927270	0.397307	0.066185
C	-4.257385	-0.339774	-0.589986
C	-2.922575	0.141641	-1.058860
C	-1.975390	-0.733125	-1.633828
C	-0.728689	-0.244767	-2.072803

C	-0.439723	1.163244	-2.103627
C	-1.396954	2.045445	-1.579990
C	-2.573652	1.527323	-0.989661
C	0.877333	1.613332	-2.674165
C	1.331089	2.986967	-2.188652
C	0.766786	1.568934	-4.211267
H	1.732042	1.840115	-4.649215
H	0.492083	0.572038	-4.569791
H	0.014223	2.284639	-4.559503
H	1.620949	0.864376	-2.368547
H	2.342493	3.182011	-2.556422
H	1.344071	3.042893	-1.096094
H	0.682125	3.781941	-2.572210
H	0.026109	-0.946914	-2.415296
H	-2.169898	-1.801343	-1.652104
H	-1.212876	3.112805	-1.546335
H	-3.260174	2.212017	-0.501333
H	-4.978832	-0.198621	-1.404125
H	-4.234264	-1.402258	-0.339914
H	-4.613212	0.233658	0.268914
S	-0.783326	3.217875	1.744873
C	-0.837464	1.453196	1.912628
C	-2.074249	0.775018	1.879765
H	-2.980965	1.333508	1.667345
H	-2.194378	-0.107949	2.499170
H	-0.003578	1.110704	2.526038
O	0.546170	3.672421	1.361509
O	-1.981528	3.758173	1.113900
F	-0.923681	3.627244	3.331153

**TS2 (imaginary frequency: -242.21 cm^{-1})**			
C	1.721622	-0.925169	0.904874
C	3.025648	-1.504478	1.104806

C	3.162433	-2.673906	1.774409
C	1.960083	-3.364894	2.346444
C	0.639017	-2.697097	2.221647
C	0.566547	-1.472235	1.551953
C	-0.639533	-0.731862	1.453218
C	-1.788480	-1.308140	2.026548
C	-1.725488	-2.549700	2.643383
C	-0.517631	-3.244160	2.766040
C	4.472286	-3.350664	1.959900
O	1.556377	0.066743	0.127238
O	2.083773	-4.444200	2.911426
H	3.877936	-1.004564	0.651289
H	-2.727271	-0.760760	2.012451
H	-2.631581	-2.975514	3.064459
H	-0.473776	-4.196597	3.284259
H	4.672423	-3.495496	3.027936
H	4.446768	-4.352415	1.515779
H	5.281975	-2.772906	1.511273
Ru	-0.457270	0.481041	-0.332029
C	-2.897341	-2.010094	-0.845203
C	-2.067196	-0.871644	-1.331234
C	-0.813380	-1.100510	-1.932763
C	-0.058212	-0.012903	-2.446449
C	-0.544512	1.316774	-2.454307
C	-1.768844	1.544300	-1.758892
C	-2.508404	0.478878	-1.188956
C	0.252456	2.402766	-3.151338
C	1.531516	2.759742	-2.382114
C	-0.578977	3.647593	-3.461313
H	0.005213	4.319102	-4.097474
H	-1.501629	3.393657	-3.994022
H	-0.838132	4.194510	-2.550182
H	0.555817	1.953538	-4.108348
H	2.126235	3.471042	-2.964094

H	2.146433	1.875206	-2.189185
H	1.277216	3.222526	-1.423495
H	0.936673	-0.208389	-2.839053
H	-0.396018	-2.101954	-1.955947
H	-2.150354	2.553629	-1.652722
H	-3.427188	0.692416	-0.652659
H	-3.471985	-2.387956	-1.700695
H	-2.278373	-2.828291	-0.469623
H	-3.608369	-1.698637	-0.077991
S	-1.173006	3.473628	0.890001
C	-0.111345	2.063511	0.980079
C	-0.555809	1.022191	1.922934
H	-1.558919	1.166844	2.317966
H	0.179355	0.832371	2.703477
H	0.923620	2.404498	1.038304
O	-0.670652	4.463795	-0.054194
O	-2.586949	3.114859	0.929560
F	-0.874799	4.129975	2.372633

**D**			
C	1.557540	-0.200389	1.079417
C	2.671537	-1.041676	0.690080
C	2.692129	-2.374407	0.906794
C	1.518570	-3.034342	1.536951
C	0.375759	-2.189835	1.961037
C	0.422994	-0.771021	1.814150
C	-0.651769	0.032079	2.312236
C	-1.775760	-0.637892	2.854100
C	-1.817979	-2.009280	2.960014
C	-0.728163	-2.784392	2.537586
C	3.829927	-3.245102	0.508846
O	1.546159	1.040789	0.753040
O	1.504931	-4.252029	1.691656

H	3.495314	-0.533304	0.194161
H	-2.593955	-0.035771	3.235986
H	-2.682538	-2.492592	3.404161
H	-0.739540	-3.863551	2.657151
H	4.213133	-3.786149	1.381411
H	3.490224	-4.008663	-0.200337
H	4.634142	-2.660982	0.058360
Ru	-0.333602	0.412759	-0.092262
C	-2.483088	-2.197031	-0.510143
C	-1.629186	-1.133905	-1.111047
C	-0.292503	-1.400281	-1.516973
C	0.518317	-0.370705	-2.018178
C	-0.002559	0.944302	-2.274013
C	-1.350735	1.180605	-1.930833
C	-2.143034	0.179413	-1.306653
C	0.869311	1.988979	-2.936562
C	2.162740	2.262271	-2.158326
C	0.136836	3.295719	-3.233910
H	0.809901	3.969067	-3.771884
H	-0.749910	3.139485	-3.856503
H	-0.167301	3.798097	-2.307735
H	1.148114	1.525031	-3.897103
H	2.810841	2.909022	-2.756875
H	2.716581	1.348902	-1.925356
H	1.934617	2.771317	-1.217078
H	1.565272	-0.575876	-2.222119
H	0.134508	-2.381878	-1.331957
H	-1.772002	2.169686	-2.053514
H	-3.155331	0.407902	-0.988936
H	-3.063270	-2.662684	-1.317455
H	-1.882808	-2.976462	-0.036415
H	-3.189752	-1.779265	0.211095
S	-2.500604	2.683859	0.937270
C	-0.825253	2.117395	1.087155

C	-0.559482	1.542770	2.474044
H	-1.269283	1.872005	3.238763
H	0.438090	1.834744	2.807303
H	-0.196545	2.960724	0.785806
O	-2.748136	3.394565	-0.314398
O	-3.468289	1.710133	1.437450
F	-2.494160	3.876096	2.084370

	**E**		
C	1.082512	-1.117212	1.140064
C	2.444406	-1.547070	1.383087
C	2.837836	-2.187863	2.503289
C	1.830211	-2.486478	3.545572
C	0.423448	-2.061460	3.312287
C	0.052311	-1.328016	2.159924
C	-1.291218	-0.916600	2.024184
C	-2.224777	-1.286244	2.993561
C	-1.847548	-2.014451	4.113701
C	-0.521600	-2.391109	4.277598
C	4.238143	-2.622988	2.749696
O	0.890877	-0.614105	-0.004578
O	2.156658	-3.068079	4.575238
H	3.149540	-1.326375	0.585420
H	-3.259308	-0.976702	2.876073
H	-2.588065	-2.277810	4.862420
H	-0.203471	-2.951288	5.150467
H	4.618091	-2.165940	3.670614
H	4.275534	-3.706931	2.906925
H	4.887280	-2.353411	1.914846
Ru	-0.661512	0.164472	-1.083418
C	-2.896274	-1.703108	-2.859889
C	-1.817058	-0.670187	-2.841439
C	-0.459275	-1.018514	-3.021249

C	0.541838	-0.020708	-2.917345
C	0.241700	1.361981	-2.752530
C	-1.133297	1.699407	-2.593362
C	-2.140409	0.706065	-2.613464
C	1.365348	2.379538	-2.722237
C	2.250542	2.206580	-1.479347
C	0.876489	3.821433	-2.848802
H	1.739919	4.485425	-2.949121
H	0.239911	3.958808	-3.729165
H	0.318222	4.133011	-1.959687
H	1.979787	2.147442	-3.604985
H	3.098256	2.896773	-1.531421
H	2.641747	1.188513	-1.394423
H	1.672984	2.428675	-0.574796
H	1.584305	-0.328748	-2.925495
H	-0.175520	-2.059248	-3.134474
H	-1.419196	2.726632	-2.394203
H	-3.169623	0.986366	-2.415397
H	-3.317294	-1.746435	-3.872043
H	-2.505548	-2.691196	-2.607960
H	-3.705442	-1.439741	-2.174171
S	-1.959330	2.699791	0.556666
C	-1.013162	1.214461	0.657176
C	-1.788142	-0.040500	0.915755
H	-1.788464	-0.772148	-0.051315
H	-2.868629	0.118274	0.975270
H	-0.148258	1.404017	1.297186
O	-1.107712	3.841274	0.247567
O	-3.232883	2.499031	-0.126329
F	-2.368014	2.898954	2.142517

TS3 (imaginary frequency: -585.43 cm^{-1})

C	1.125341	-1.093462	1.176154

C	2.489493	-1.487106	1.454919
C	2.852779	-2.161003	2.567072
C	1.811919	-2.533210	3.552917
C	0.407400	-2.124332	3.285529
C	0.073723	-1.341515	2.156000
C	-1.263192	-0.924514	1.984319
C	-2.236521	-1.358821	2.888400
C	-1.899326	-2.147289	3.979820
C	-0.575672	-2.515143	4.187769
C	4.253006	-2.572034	2.853285
O	0.939031	-0.593715	0.026890
O	2.111407	-3.159092	4.565149
H	3.219432	-1.224238	0.693156
H	-3.267362	-1.048479	2.743270
H	-2.669588	-2.463190	4.676462
H	-0.291116	-3.117113	5.044354
H	4.583311	-2.145140	3.807280
H	4.312760	-3.660400	2.966872
H	4.928032	-2.251133	2.058051
Ru	-0.665009	0.120905	-0.988764
C	-2.952828	-1.674632	-2.819456
C	-1.867208	-0.649200	-2.773979
C	-0.506444	-1.009588	-2.960013
C	0.506430	-0.021344	-2.925779
C	0.220285	1.355863	-2.758067
C	-1.139077	1.692995	-2.507812
C	-2.168380	0.717643	-2.521405
C	1.343893	2.372125	-2.787124
C	2.236223	2.252028	-1.542001
C	0.852869	3.807378	-2.972660
H	1.714499	4.464469	-3.121737
H	0.197676	3.902769	-3.844993
H	0.313895	4.162715	-2.088110
H	1.953767	2.101633	-3.661438

H	3.081658	2.942121	-1.625268
H	2.629434	1.238154	-1.421454
H	1.663316	2.508570	-0.643549
H	1.543461	-0.338305	-3.002821
H	-0.236147	-2.052925	-3.082471
H	-1.408120	2.722595	-2.297401
H	-3.190793	1.016264	-2.317077
H	-3.333069	-1.727121	-3.846990
H	-2.577887	-2.662417	-2.543295
H	-3.784206	-1.398724	-2.166816
S	-1.864684	2.758321	0.592007
C	-0.949295	1.258588	0.743444
C	-1.705549	0.033123	0.941116
H	-1.591075	-0.976042	-0.270626
H	-2.789788	0.120164	0.869662
H	-0.051393	1.438349	1.337744
O	-0.998581	3.869941	0.222166
O	-3.154331	2.552935	-0.056716
F	-2.228400	3.021578	2.174014

	**F**		
C	1.150621	-1.044588	1.199359
C	2.524394	-1.370103	1.519999
C	2.876085	-2.057302	2.627288
C	1.814517	-2.517167	3.555663
C	0.405408	-2.151376	3.254521
C	0.083309	-1.338877	2.142520
C	-1.256544	-0.939387	1.941594
C	-2.245068	-1.444047	2.795534
C	-1.922293	-2.276371	3.857294
C	-0.594677	-2.611866	4.102030
C	4.282060	-2.408725	2.959670
O	0.973531	-0.562571	0.039940

O	2.104730	-3.176429	4.548746
H	3.268208	-1.054305	0.792588
H	-3.278155	-1.149778	2.633127
H	-2.706576	-2.647888	4.509708
H	-0.322666	-3.240056	4.943785
H	4.549751	-1.999826	3.940840
H	4.392143	-3.496108	3.039800
H	4.972247	-2.026658	2.205569
Ru	-0.684531	0.037536	-0.952753
C	-3.070257	-1.591783	-2.785700
C	-1.956545	-0.596820	-2.732985
C	-0.600429	-0.992227	-2.979504
C	0.439642	-0.046794	-2.983861
C	0.200384	1.330688	-2.746788
C	-1.139352	1.702748	-2.463297
C	-2.205946	0.768098	-2.469761
C	1.350213	2.315756	-2.796619
C	2.258227	2.170859	-1.565390
C	0.896872	3.764343	-2.976855
H	1.774295	4.395642	-3.144038
H	0.228202	3.875961	-3.836990
H	0.385659	4.137992	-2.083528
H	1.938768	2.027368	-3.679631
H	3.122671	2.834699	-1.665003
H	2.621245	1.145408	-1.448659
H	1.709480	2.448240	-0.658067
H	1.459649	-0.390843	-3.135025
H	-0.372166	-2.041545	-3.135666
H	-1.368153	2.738488	-2.235289
H	-3.213409	1.102593	-2.248018
H	-3.451157	-1.629319	-3.813494
H	-2.722473	-2.590842	-2.514314
H	-3.891864	-1.296863	-2.129429
S	-1.807558	2.779032	0.594519

C	-0.917507	1.259688	0.748720
C	-1.680555	0.060960	0.946365
H	-1.275203	-1.362371	-0.564820
H	-2.759913	0.130064	0.824165
H	0.005240	1.437253	1.304156
O	-0.929883	3.875856	0.208477
O	-3.112971	2.596871	-0.027907
F	-2.128563	3.040427	2.182963

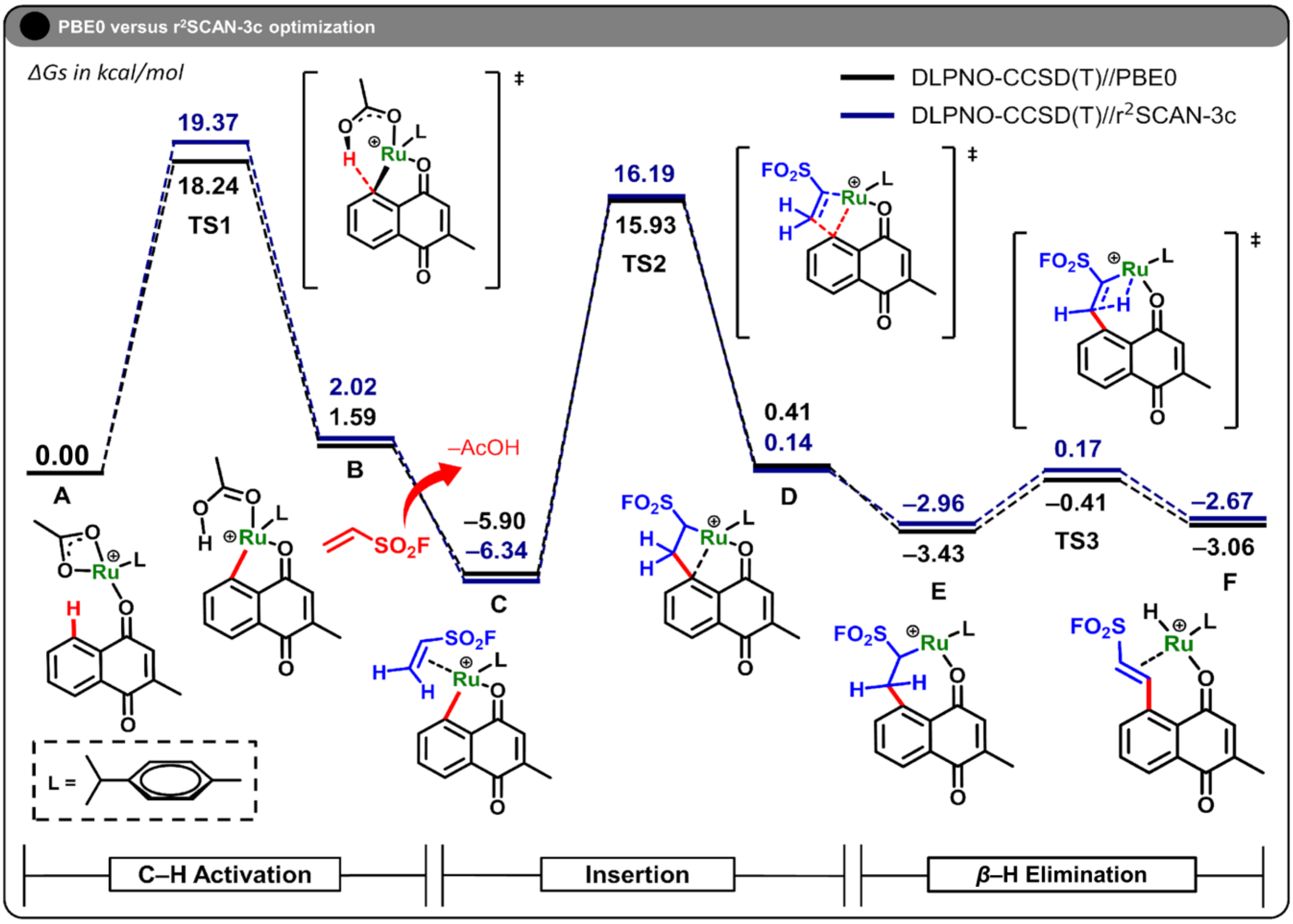

Figure S9. Computed Gibbs free energies in (kcal/mol) calculated for the first step for the C–H alkenylation of the menadione (**1a**). Energies were obtained with CCSD(T) method, from optimized structures at r^{2}SCAN-3c/CPCM(DCE) (blue line) and PBE0-D3(BJ)/bs1+CPCM(DCE) (black line). Herein, bs1 = def2-TZVP for Ru, def2-SVP for other elements; DCE = 1,2-dichloroethane.
